# Co-dependent excitatory and inhibitory plasticity accounts for quick, stable and long-lasting memories in biological networks

**DOI:** 10.1038/s41593-024-01597-4

**Published:** 2024-03-20

**Authors:** Everton J. Agnes, Tim P. Vogels

**Affiliations:** 1 Centre for Neural Circuits and Behaviour, University of Oxford, Oxford, UK; 2 Biozentrum, University of Basel, Basel, Switzerland; 3 Institute of Science and Technology Austria, Klosterneuburg, Austria

**Keywords:** Synaptic plasticity, Learning algorithms, Network models

## Abstract

The brain’s functionality is developed and maintained through synaptic plasticity. As synapses undergo plasticity, they also affect each other. The nature of such ‘co-dependency’ is difficult to disentangle experimentally, because multiple synapses must be monitored simultaneously. To help understand the experimentally observed phenomena, we introduce a framework that formalizes synaptic co-dependency between different connection types. The resulting model explains how inhibition can gate excitatory plasticity while neighboring excitatory–excitatory interactions determine the strength of long-term potentiation. Furthermore, we show how the interplay between excitatory and inhibitory synapses can account for the quick rise and long-term stability of a variety of synaptic weight profiles, such as orientation tuning and dendritic clustering of co-active synapses. In recurrent neuronal networks, co-dependent plasticity produces rich and stable motor cortex-like dynamics with high input sensitivity. Our results suggest an essential role for the neighborly synaptic interaction during learning, connecting micro-level physiology with network-wide phenomena.

## Abstract (web summary)

How do multiple synapses interact to modulate learning? Agnes and Vogels postulate models of ‘co-dependent’ synaptic plasticity that promote rapid, multi-synaptic attainment of stable receptive fields, dendritic patterns and plausible neural dynamics.

## Main

Synaptic plasticity is thought to be the brain’s fundamental mechanism for learning^1–3^. Based on Hebb’s postulate and early experimental data, theories have focused on the idea that synapses change based solely on the activity of their presynaptic and postsynaptic counterparts^4–10^, defining synaptic plasticity as predominantly a synapse-specific process. However, experimental evidence^11–20^ has pointed toward learning mechanisms that act locally at the mesoscale, taking into account the activity of multiple synapses and synapse types nearby. For example, excitatory synaptic plasticity (ESP) has long been known to rely on intersynaptic cooperativity by way of elevated calcium concentrations from multiple presynaptically active excitatory synapses^15–18^. Interestingly, GABAergic, inhibitory synaptic plasticity (ISP) has also been shown to depend on the activation of neighboring excitatory synapses: ISP is blocked when nearby excitatory synapses are deactivated^11,12^, and the magnitude of the changes depends on the ratio between local excitatory and inhibitory currents (EI balance)^11^. Moreover, the absence of inhibitory currents can either flip the direction^13,14^ or maximize ESP^21–23^. The amplitude of long-term potentiation (LTP) at excitatory synapses also depends on the history of nearby excitatory LTP induction, revealing temporal and distance-dependent effects^24^. Finally, Hebbian LTP can also trigger long-term depression (LTD) at neighboring synapses^19^ through a heterosynaptic plasticity mechanism—that is, without the need of presynaptic activation. There is currently no unifying framework to incorporate these experimentally observed interdependencies at the mesoscopic level of synaptic plasticity.

Existing models typically aim to explain, for example, how cell assemblies are formed and maintained^9,25^. In these studies, synapse-specific plasticity rules are typically complemented with global processes, such as normalization of excitatory synapses^25^ or modulation of inhibitory synaptic plasticity by the average network activity^9^, for stability. Moreover, intricate spatiotemporal dynamics, such as the activity patterns observed in motor cortex during reaching movements^26^, can be reproduced only when inhibitory connections are optimized (that is, hand tuned) by iteratively changing the eigenvalues of the connectivity matrix toward stable values^27,28^ or learned by non-local supervised algorithms, such as FORCE^29,30^. However, models that rely on connectivity changes triggered by non-local quantities are usually based on the optimization of network dynamics^27–30^ and often do not reflect biologically relevant mechanisms (but see ref. ^31^).

To fill the theoretical gap in mesoscopic, yet local, synaptic plasticity rules, we introduce a new model of ‘co-dependent’ synaptic plasticity that includes the direct interaction between different neighboring synapses. Our model accounts for a wide range of experimental data on excitatory plasticity and receptive field plasticity of excitatory and inhibitory synapses and makes predictions for future experiments involving multiple synaptic stimulation. Furthermore, it provides a mechanistic explanation for experimentally observed synaptic clustering and for how dendritic morphology can facilitate the emergence of single (clustered) or mixed (scattered) feature selectivity. Finally, we show how naive recurrent networks can grow into strongly connected, stable and input-sensitive circuits showing amplifying dynamics.

## Results

**Fig. 1:**
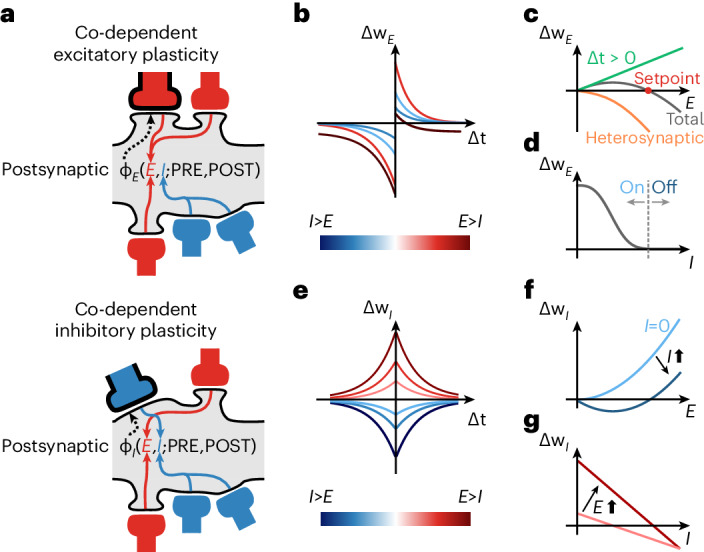
Co-dependent synaptic plasticity model. **a**, Co-dependent excitatory (top) and inhibitory (bottom) plasticity. Plasticity of a synapse (highlighted with black contour) depends on the activation of its neighboring excitatory (red) and inhibitory (blue) synapses, together with its synapse-specific presynaptic and postsynaptic activity—that is, spike times, indicated by PRE and POST, respectively. Variables *E* and *I* integrate NMDA and GABAergic currents (low-pass filters), respectively. **b**, Excitatory weight change, Δ*w*_*E*_, as a function of the time interval between postsynaptic and presynaptic spikes, Δ*t*, and neighboring synaptic inputs, *E* and *I*. $\Delta t={t}_{{{{\rm{post}}}}}-{t}_{{{{\rm{pre}}}}}$, where *t*_post_,and ${t}_{{{{\rm{pre}}}}}$ are spike times of postsynaptic and presynaptic neurons, respectively, so that Δ*t* > 0 for pre-before-post and Δ*t* < 0 for post-before-pre spike patterns. **c**, Excitatory inputs, *E*, control Hebbian LTP (green line; Δ*t* > 0) and heterosynaptic plasticity (orange line), which combined (gray line) create a common setpoint for the total excitatory input (red dot). **d**, Inhibitory inputs, *I*, gate excitatory plasticity (‘ON’ versus ‘OFF’). **e**, Inhibitory weight change, Δ*w*_*I*_, is a function of Δ*t* and neighboring synaptic inputs (as in **b**). **f**,**g**, Synaptic changes in inhibitory synapses as a function of excitatory (**f**) and inhibitory (**g**) inputs.

We developed a general theoretical framework for synaptic plasticity rules that accounts for the interplay between different synapse types during learning. In our framework, excitatory and inhibitory synapses change according to the functions *ϕ*_E_(*E*, *I*; PRE, POST) and *ϕ*_I_(*E*, *I*; PRE, POST), respectively (Fig. 1a). The signature of the co-dependency between neighboring synapses—that is, synapses that are within each others’ realm of physical influence—is given by *E* and *I*, which describe the recent postsynaptic activation of nearby excitatory and inhibitory synapses. The activity of the synapses’ own presynaptic and postsynaptic neurons—that is, the local synapse-specific activity—is described by the variables PRE and POST. We modeled *E* and *I* as variables that integrate neighboring synaptic currents: calcium influx through *N*-methyl-d-aspartate (NMDA) channels for *E* and chloride influx through *γ*-aminobutyric acid type A (GABA_A_) channels for *I*. The implementation of excitatory and inhibitory plasticity rules varies slightly, as follows below.

### Co-dependent excitatory plasticity model

The rule *ϕ*_E_(*E*, *I*; PRE, POST) by which excitatory synaptic efficacy change is constructed similarly to classic spike-timing-dependent plasticity (STDP) models^15,32^: pre-before-post spike patterns may elicit potentiation (details below), whereas post-before-pre elicits depression (Fig. 1b). Synaptic changes are also modulated by ‘neighboring’ excitatory and inhibitory activity (Fig. 1a). Initially, we defined an explicit distance-dependent term so that the influence between two neighboring synapses decays with their separation (Methods). In later models, we assumed, for simplicity, that all synapses onto a dendritic compartment or postsynaptic neuron contribute equally to the variables *E* and *I*, such that all synapses onto a dendritic compartment or postsynaptic neuron are neighbors with each other.

In addition to the STDP component, the learning rate for potentiation increases linearly with the magnitude of neighboring (including the synapse’s own) NMDA currents^15,16,18^ (Fig. 1c, green line). This destabilizing positive feedback, in which potentiation leads to bigger excitatory currents, which, in turn, leads to more potentiation, is counterbalanced by introducing a heterosynaptic term^9^ that weakens a synapse via a quadratic dependency on its neighboring (including the synapse’s own) NMDA currents (Fig. 1c, orange line). This term is based on experimentally observed heterosynaptic weakening of excitatory synapses neighboring other synapses undergoing LTP^19^. Together, potentiation and heterosynaptic weakening form a fixed point in the dynamics of synaptic weights. As a result, weak to intermediate excitatory currents elicit strengthening, whereas strong currents induce weakening (Fig. 1c, gray line). In addition to neighboring excitatory–excitatory effects, we constructed the model such that elevated inhibition blocks excitatory plasticity: only when synapses are disinhibited can excitatory plasticity change their efficacies (Fig. 1d). Inhibition thus directly modulates excitatory plasticity in our model, complementing the indirect influence of inhibition on excitatory plasticity via the direct influence of inhibition on the postsynaptic neurons’ membrane potential and spike times. This direct control of inhibition over excitatory plasticity allows for rapid, one-shot-like learning^33^ during periods of disinhibition^34^ in behavioral timescales—that is, when multiple presynaptic excitatory spikes coincidentally activate a postsynaptic neuron, because the effective learning rate can vary wildly (through rapid intermittent disinhibition) without compromising the stability of the network. At all other times—when inhibition is strong enough to effectively block excitatory plasticity—excitatory weights cannot drift due to ongoing presynaptic and postsynaptic activity.

Changes in a given excitatory synapse, *w*_E_, denoted by Δ*w*_E_, are expressed in a simplified way as:

1 $$\begin{array}{l} \Delta {w_{\rm{E}}} = {\phi_{\rm{E}}}(E,I;PRE,POST)\\ \kern 2pc = \left[A_{\rm{LTP}}\left(PRE_{\rm{LTP}}\right)\left(POST_{\rm{spike}}\right)E \right. \kern 2.5pc {\mbox{LTP (pre-before-post)}}\\ \kern 3pc-A_{\rm{het}}\left(POST_{\rm{het}}\right)\left(POST_{\rm{spike}}\right)E^2 \kern 2.1pc{\mbox{heterosynaptic}}\\ \kern 3pc-A_{\rm{LTD}}\left.\left(POST_{\rm{LTD}}\right)\left(PRE_{\rm{spike}}\right)w_{\rm{E}}\right] \kern 1.2pc {\mbox{LTD (post-before-pre)}}\\ \kern 3pc\times\exp\left[-\left(\frac{I}{I^*}\right)^\gamma\right] \kern 8.2pc{\mbox{inhibitory control}},\end{array}$$

where *A*_LTP_, *A*_het_ and *A*_LTD_ are the (strictly positive) learning rates for the LTP, heterosynaptic and LTD plasticity terms, respectively (see Methods for the detailed implementation). The terms $\left(PR{E}_{{{{\rm{LTP}}}}}\right)$, $\left(POS{T}_{{{{\rm{het}}}}}\right)$ and $\left(POS{T}_{{{{\rm{LTD}}}}}\right)$ represent the filtered spike trains (that is, firing rate estimates) of presynaptic and postsynaptic neurons. Spike times of presynaptic and postsynaptic neurons are represented by $\left(PR{E}_{{{{\rm{spike}}}}}\right)$ and $\left(POS{T}_{{{{\rm{spike}}}}}\right)$, respectively, which trigger synaptic weight changes. The parameters I* and *γ* define the inhibitory control over excitatory plasticity. The amplitude of excitatory-to-excitatory plasticity is maximum when inhibition is blocked, decreasing monotonically with the magnitude of local inhibitory currents. Interestingly, both weight-dependent STDP^32,35^ and triplet learning rules^5^ can be recovered from equation (1) under certain approximations and simplifications (see the Supplementary Modeling Note for details).

### Co-dependent inhibitory plasticity model

Inhibitory synapses change according to a function *ϕ*_I_(*E*, *I*; PRE, POST) that follows a symmetric STDP curve^3,11,36^ (Fig. 1e)—synaptic changes are scaled according to the temporal proximity of presynaptic and postsynaptic spikes. Similar to excitatory plasticity, the learning rate of inhibitory plasticity is modulated by neighboring excitatory and inhibitory activity (Fig. 1f,g). In this case, when *E* and *I* (that is, NMDA and GABAergic currents) are equal (*E* = *I*), or when NMDA currents vanish (*E* = 0), there is no change in the efficacy of inhibitory synapses: they remain constant. LTP is induced when excitatory currents are stronger than inhibitory ones and vice versa for LTD. As a consequence, spike times and neighboring synaptic currents act together but at different timescales. These co-dependent components of ISP are based on the abolition of either LTP^12^ or both LTP and LTD^11^ when postsynaptic NMDA currents are blocked as well as evidence of increase in amplitude of changes for larger EI ratios^11^.

Changes in a given inhibitory synapse, *w*_I_, denoted by Δ*w*_I_, are expressed in a simplified way as:

2 $$\begin{array}{lll} \Delta w_{\rm{I}} = \phi_{\rm{I}}(E,I;PRE,POST)\\ \kern 1.8pc = A_{\rm{ISP}} E\left(E-\alpha I\right) \kern 3.9pc{\mbox{codependency}}\\ \kern 2.5pc\times\left[\left(PRE_{\rm{inh}}\right)\left(POST_{\rm{spike}}\right)\right. \kern 0.8pc {\mbox{pre-before-post}}\\ \kern 2.5pc+\left.\left(POST_{\rm{inh}}\right)\left(PRE_{\rm{spike}}\right)\right] \kern 0.8pc{\mbox{post-before-pre}},\end{array}$$

where *A*_ISP_ is the (strictly positive) learning rate for the co-dependent inhibitory synaptic plasticity rule, and *α* is the EI balance setpoint imposed by the learning rule, such that *E* / *I* = *α* (see Methods for the detailed implementation). The terms $\left(PR{E}_{{{{\rm{inh}}}}}\right)$ and $\left(POS{T}_{{{{\rm{inh}}}}}\right)$ represent the filtered spike trains (that is, firing rate estimates) of presynaptic and postsynaptic neurons. Spike times of presynaptic and postsynaptic neurons are represented by $\left(PR{E}_{{{{\rm{spike}}}}}\right)$ and $\left(POS{T}_{{{{\rm{spike}}}}}\right)$, respectively, which trigger synaptic weight changes. Applying specific simplifications to equation (2), we can recover a previously proposed spiked-based learning rule^7^, similarly to the above case for excitatory synapses (see the Supplementary Modeling Note for details).

### Stability of excitatory currents

We implemented the above rules in a single leaky integrate-and-fire (LIF) neuron with plastic excitatory synapses that emulate *α*-amino-3-hydroxy-5-methyl-4-isoxazolepropionic acid (AMPA) and NMDA receptors as well as inhibitory (GABA_A_) synapses (Methods). We initially assessed the properties of co-dependent excitatory plasticity with regard to previous experimental^15,16,24^ and modeling studies^5,6,8,37,38^, as described below.

**Fig. 2:**
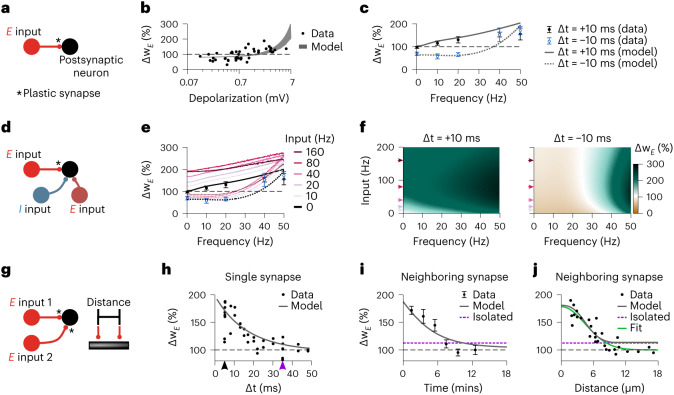
Co-dependent excitatory synaptic plasticity: influence of voltage, firing frequency and synaptic distance. **a**, Schematic of the protocol used in **b** and **c**: two connected excitatory neurons. **b**, Simulation of 10-ms pre-before-post STDP protocol as a function of depolarization, capturing observed voltage influence of excitatory plasticity^16^. **c**, Simulation of pre-before-post (+10 ms) and post-before-pre (−10 ms) STDP protocols at various frequencies, capturing observed firing frequency influence of STDP^15^. **d**, Schematic of the protocol used in **e** and **f**: one excitatory postsynaptic neuron receiving one plastic excitatory synapse and two static (inhibitory and excitatory) neighboring synapses. **e**, Same as **c** for different firing rates of neighboring synapses (color coded). **f**, Weight change as a function of neighboring synapsesʼ input frequency (*y* axis) and frequency of spike pairs (*x* axis). Arrows indicate external frequencies used in **e**. **g**, Schematic of the protocol used in **h**–**i**: two presynaptic excitatory neurons connected to a single postsynaptic neuron via plastic excitatory synapses. The two synapses are separated by a given distance explicitly simulated in the plasticity model (Methods). **h**, Weight change of a single synapse as a function of the timing between the presynaptic spike and the first postsynaptic spike of a three-spike burst^24^. Black and purple arrowheads indicate the two pairings used for inducing strong and weak LTP, respectively, at neighboring synapses in **i** and **j**. **i**, Weight change of the synapse undergoing weak LTP induction as a function of the timing between its induction and a prior strong LTP induction at a neighboring synapse 3 μm apart. **j**, Weight change of the synapse undergoing weak LTP induction 90 s after strong LTP induction at a neighboring synapse as a function of their distance. Purple lines in **i** and **j** show changes of an isolated synapse (from **h**). Error bars indicate s.e.m. Experimental data in **b**,**c**,**e**,**h–j**, were adapted with permission from the following references: **b** from ref. ^16^, **c** and **e** from ref. ^15^ and **h–j** from ref. ^24^ (we refer to ref. ^15^ and ref. ^24^ for information about sample sizes and statistical analysis).

**Extended Data Fig. 1:**
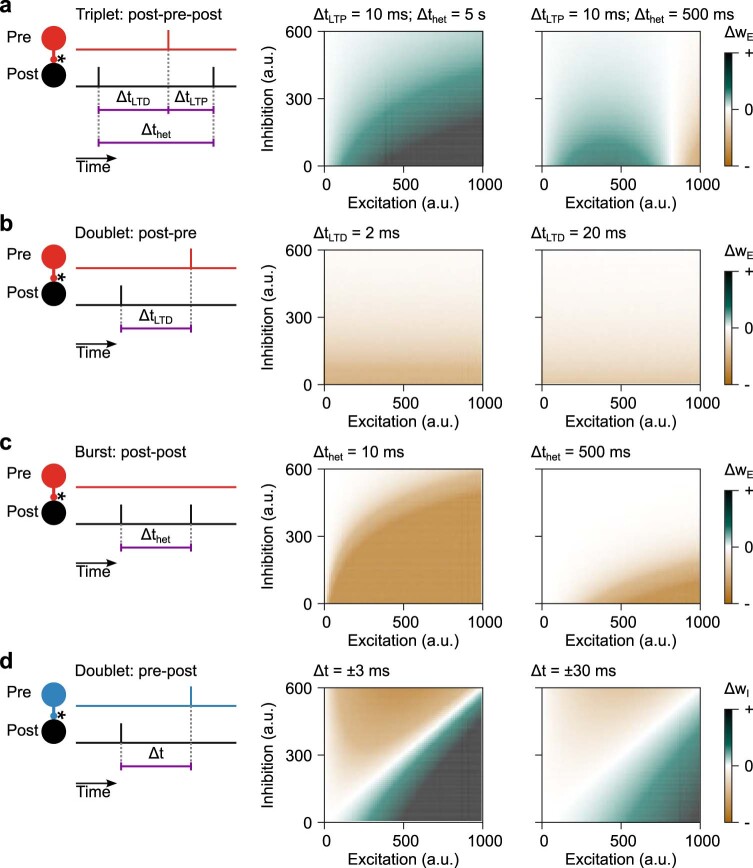
Contribution of spike times, excitation, and inhibition to weight changes for the codependent synaptic plasticity model. **a-c**, Schematics of the sequence of spikes (left), and the resulting weight change for two different spike patterns (middle and left) for codependent excitatory synaptic plasticity model as a function of the levels of excitation and inhibition during plasticity. **a**, Spike triplet: post-pre-post sequence with fixed pre-before-post spike interval, Δ*t*_LTD_, and two examples for intervals between two consecutive postsynaptic spikes, Δ*t*_het_. **b**, Doublet: post-before-pre spike pattern with two different intervals, Δ*t*_LTD_. **c**, Postsynaptic burst with two spikes at different interspike intervals, Δ*t*_het_. **d**, Same as panel b for codependent inhibitory synaptic plasticity model.

First, we considered two otherwise isolated excitatory neurons, so that there was no influence of other presynaptic partners over synaptic changes aside from the synapse that we investigated (Fig. 2a). We found that our model—in agreement with previous models^6,38,39^—could capture the influence of membrane potential depolarization due to strong initial excitatory weight, current clamp or backpropagating action potential (Supplementary Fig. 1) on synaptic efficacy changes. As a result, an LTD-inducing pre-before-post spike protocol became LTP inducing when accompanied by large postsynaptic depolarization^15,16^ (Fig. 2b). In our model, the switch from LTD to LTP was due to an increase in the magnitude of the presynaptic excitatory current through NMDA channels for depolarized states, eliciting stronger LTP (Fig. 1c and Extended Data Fig. 1a).

**Extended Data Fig. 2:**
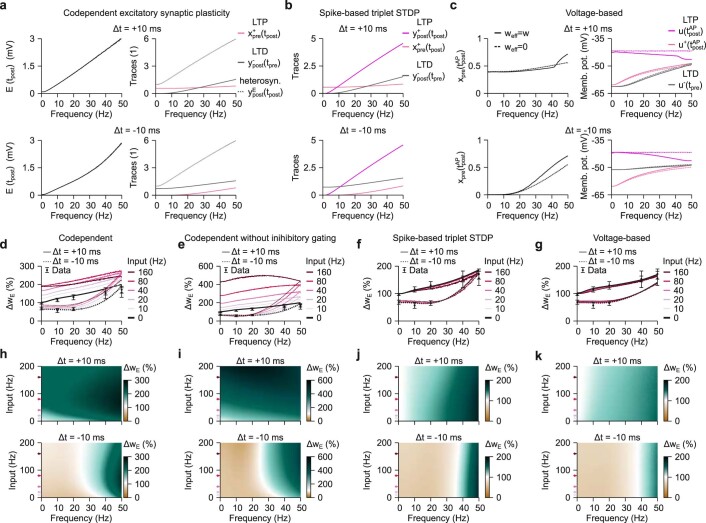
Comparison between synaptic plasticity models using the frequency-dependent STDP protocol. **a-c**, Relevant variables at the moment of synaptic plasticity induction as a function of the frequency of spike pairs with pre-before-post (top; Δ*t* = + 10 ms) and post-before-pre (bottom; Δ*t* = − 10 ms). Synaptic plasticity is induced at the moment of either a postsynaptic spike, *t*_post_ (or ${t}_{{{{\rm{post}}}}}^{{{{\rm{AP}}}}}$), or a presynaptic spike, ${t}_{{{{\rm{pre}}}}}$. **a**, Average of the traces of NMDA currents (left; *E*(*t*_post_)), presynaptic spikes (right; ${x}_{{{{\rm{pre}}}}}^{+}$ (*t*_post_)), and postsynaptic spikes (right; ${y}_{{{{\rm{post}}}}}^{{{{\rm{E}}}}}$ (*t*_post_) and ${t}_{{{{\rm{post}}}}}^{-}$ (${t}_{{{{\rm{pre}}}}}$)). **b**, Same as panel a, right, for the spike-based triplet STDP model. **c**, Average of the traces of presynaptic spikes (left; ${t}_{{{{\rm{pre}}}}}$ (${t}_{{{{\rm{post}}}}}^{{{{\rm{AP}}}}}$)) and postsynaptic membrane potential (right; *u*(${t}_{{{{\rm{post}}}}}^{{{{\rm{AP}}}}}$), *u*^+^ (${t}_{{{{\rm{post}}}}}^{{{{\rm{AP}}}}}$), and *u*^+^ (${t}_{{{{\rm{pre}}}}}$)). Dashed and continuous lines show averages for zero (*w*_eff_ = 0) and non-zero (*w*_eff_ = *w*) synaptic weights. **d-g**, Plasticity inducing protocol for different models with pairs of pre-before-post (Δ*t* = + 10 ms) and post-before-pre (Δ*t* = − 10 ms) for varying spiking frequencies, and different firing-rates of neighbouring excitatory and inhibitory afferents (colour coded). Plots show changes in synaptic weight of a single connection while the other two (excitatory and inhibitory) are kept fixed. Spike-based triplet spike-timing-dependent plasticity model from ref. ^5^ and voltage-based plasticity model from ref. ^6^. **h-k**, Weight change as a function of neighbouring synapses’ input frequency (y-axis), and frequency of spike pairs (x-axis). Arrows indicate external frequencies used in panels d-g. Plots from panels d and h are also shown in Fig. 2e,f. Error bars indicate SEM. Experimental data in panels d-g was adapted with permission from ref. ^15^ (we refer to ref. ^15^ for information about sample sizes and statistical analysis).

Similarly, the interaction of presynaptic and postsynaptic spikes could also account for efficacy changes based on the frequency of spike pair presentations (Fig. 2c). Notably, in our model, high frequency of presynaptic and postsynaptic spike pairs elicited increased LTP (Fig. 2c) due to a direct elevation in NMDA currents (Extended Data Fig. 2a and Fig. 1c). Spike-based^5,9^ or voltage-based^6^ models imitate the influence of spike frequency on LTP amplitudes by reacting to an increase in the postsynaptic firing frequency and the consequent increase in spike triplets (post-pre-post; Extended Data Fig. 2b,c). Our model thus varies in the locus of its mechanism: elevated excitatory currents—that is, a presynaptic-driven effect—instead of elevated postsynaptic activity.

In our model, plasticity could be affected by excitatory and inhibitory currents, altering amplitude and direction of synaptic change (Extended Data Fig. 1a–c). To highlight this co-dependent effect, we simulated the classic frequency-dependent protocol^15^ with a pair of neighboring synapses (one excitatory and one inhibitory with static weights) simultaneously activated (Fig. 2d). An increase in neighboring firing rate amplified LTP, which was induced by the synapse-specific pre-before-post spike pattern (Fig. 2e, full lines, and Fig. 2f, left). The same increase in neighbring firing rate reduced LTD, lowering the pairing frequency for which LTD becomes LTP for synapse-specific post-before-pre spike patterns (Fig. 2e, dashed lines, and Fig. 2f, right). These effects arose from elevated NMDA currents from the neighboring excitatory synapse (Extended Data Fig. 1a) and are magnified without inhibitory control (Extended Data Fig. 2e,i). In contrast, in the traditional spike-based^5,9^ or voltage-based^6^ learning rules, neighboring activation does not affect plasticity as long as it does not influence presynaptic and postsynaptic spike patterns or the mean postsynaptic membrane potential^37^ (Extended Data Fig. 2d–k)—that is, due to balanced excitatory and inhibitory currents (Supplementary Fig. 2).

To further investigate the distance and temporal effects of multiple presynaptic activation, we simulated a single postsynaptic neuron connected with two presynaptic excitatory synapses separated by a defined electrotonic distance (Fig. 2g). Similar to experiments in mice cortical slices^24^, the activation of a single synapse, when followed by a three-spike burst of the postsynaptic neuron with a time lag Δ*t*, induced a STDP-like change in efficacy (Fig. 2h). Repeating the same protocol with a time lag of Δ*t* = 5 ms between presynaptic and postsynaptic spikes to induce ‘strong’ LTP (black arrowhead in Fig. 2h) followed by a second, ‘weak’ LTP at a neighboring synapse with a time lag of Δ*t* = 35 ms (purple arrowhead in Fig. 2h), shortly after, reproduced the experimentally reported temporal (Fig. 2i) and spatial (Fig. 2j) dependencies of excitatory synaptic plasticity^24^ in our model.

**Fig. 3:**
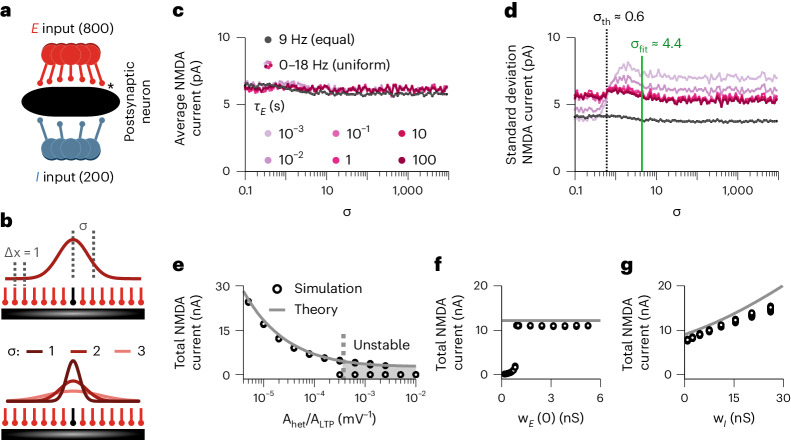
Effect of distance dependence for excitatory current stability. **a,b**, Schematics of the simulation. **a**, A single postsynaptic neuron receives 800 plastic excitatory (*) and 200 static inhibitory synapses. **b**, Top, all excitatory synapses are assumed to form a one-dimensional (1D) (line) connectivity pattern, with two consecutive synapses being separated by a unitary distance (normalized distance; Δ*x* = 1). The effect of neighboring activation is weighted by a Gaussian curve centered at the synapse undergoing plasticity (black synapse) defined by a standard deviation, *σ*. Bottom, three examples for different *σ* values (*σ* = 1, 2 and 3). To compare different values of *σ* (**c** and **d**), the peak of the distance dependent interaction was normalized by the area under the curve. **c**,**d**, Average (**c**) and standard deviation (**d**) of the excitatory NMDA currents per synapse after learning as a function of the standard deviation, which defined the distance-dependent effect, *σ*. Gray dots represent simulations in which all presynaptic neuronsʼ firing rates are equal. Colored dots represent simulations in which individual excitatory presynaptic neuronsʼ firing rates are uniformly distributed between 0 Hz and 18 Hz. Each color indicates a different characteristic time for the excitatory current filter, *E* (equation (1)). All inhibitory neurons have a constant firing rate of 18 Hz. *σ*_th_ ≈ 0.6 defines the transition from effectively non-interacting (*σ* < *σ*_th_) to interacting (*σ* > *σ*_th_) synapses, whose steady-state distributions of synapse-specific NMDA currents differ (Extended Data Fig. 3). *σ*_fit_ ≈ 4.4 is the value fitted to the experimental curve (green curve in Fig. 2j; *σ* = 4.4 μm) assuming an average distance of 1 μm between neighboring synapses. **e**–**g**, Total excitatory NMDA current after learning as a function of the ratio between heterosynaptic and LTP learning rates (**e**), initial excitatory weights (**f**) and inhibitory weights (**g**). Continuous lines indicate a simplified analytical solution (Methods). The dashed line in **e** indicates the threshold for which the heterosynaptic plasticity term may induce vanishing of weights (shaded region; Methods).

We extended the above protocol and simulated a single postsynaptic neuron receiving homogeneous Poisson excitatory and inhibitory spike trains from synapses with spatial organization (Fig. 3a,b and Methods). For simplicity, we modeled excitatory synapses as equally spaced along a single-compartment neuron with equal, unitary distance between immediate neighbors (Fig. 3b, top). The influence of a given synapse onto another was implemented according to their assumed electrotonic distance as a normalized current following a Gaussian-shaped decay with standard deviation *σ* (Fig. 3b). *σ* thus characterized the topology of spatial interactions. It means that the maximum influence on a synapse was its own NMDA current influx (center of the Gaussian). Other synapses also contributed to the efficacy change, with the amplitude of their effect normalized by the length of interactions, *σ*, and number of neighboring synapses (Fig. 3b, bottom, and Methods). After the system reached equilibrium, we found that the mean excitatory current influx through NMDA channels was independent of the length constant, *σ* (Fig. 3c), as a result of the combination of the Hebbian LTP and heterosynaptic terms, which produces a setpoint for the total NMDA currents (Methods and Fig. 1c, red circle).

**Extended Data Fig. 3:**
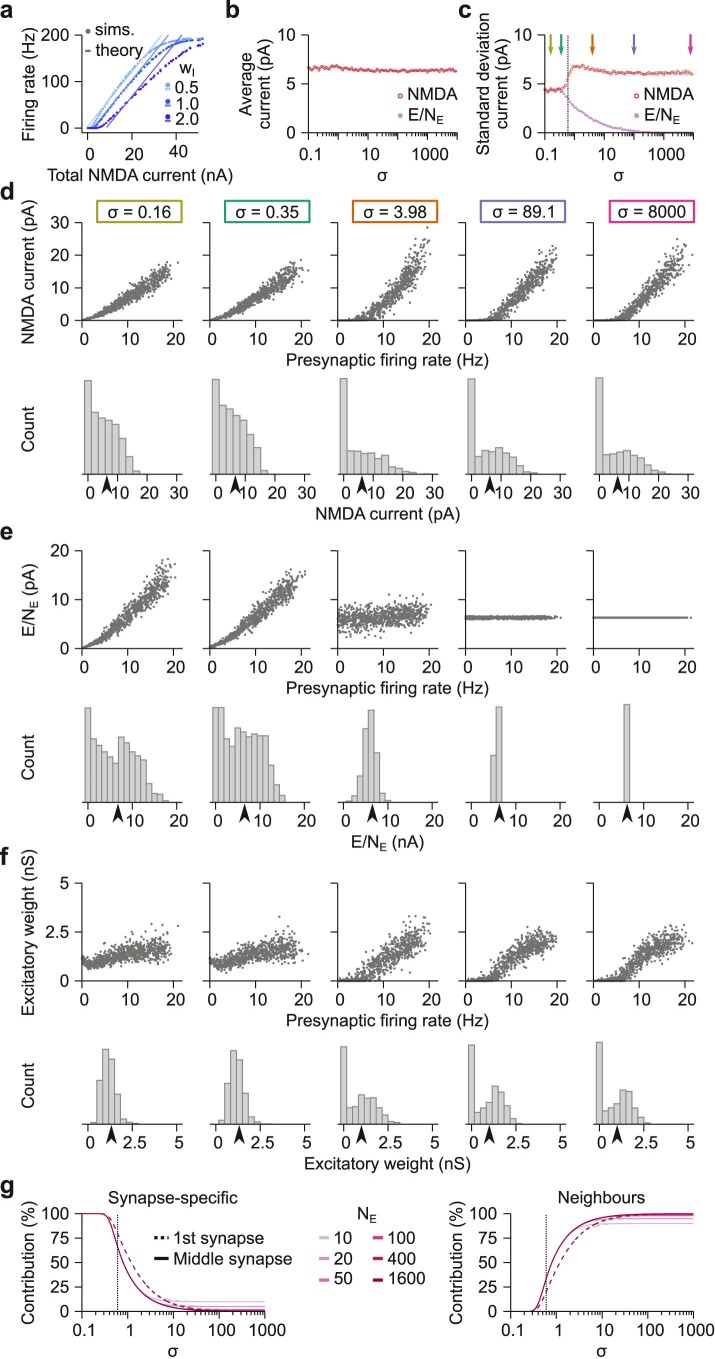
Effect of distance dependence for excitatory current and weight stability. **a**, Firing-rate of a single postsynaptic neuron as a function of the total NMDA current for three different inhibitory weights. Points from simulations and lines from fitting the points to Eq. (36). **b**, Average NMDA currents (red open circles; same plot from Fig. 3b) and average filtered NMDA currents (variable *E*) divided by the number of excitatory synapses, *N*_E_ (pink filled circles) for *τ*_E_ =10 ms. **c**, Standard deviation of the NMDA currents (red open circles; same plot from Fig. 3b) and standard deviation of the filtered NMDA currents (variable *E*) divided by the number of excitatory synapses, *N*_E_ (pink filled circles) for *τ*_E_ =10 ms. Arrows indicate which values were used in the plots from panels d to f. **d**, Top: Temporal average of the NMDA currents after learning for each excitatory synapse as a function of the presynaptic firing-rate for distinct values of *σ* (see arrows in panel b, right). Bottom: Distribution of the NMDA currents after learning (from plots above). Arrowheads indicate the mean. **e**, Same as panel d for the filtered NMDA currents (variable *E*) divided by the number of excitatory synapses, *N*_E_. Notice that for large *σ*, *E*/*N*_E_ becomes independent of the input firing-rate. **f**, Same as panel d for the synaptic weights. **g**, Influence of synapses on the excitatory synaptic plasticity. Dashed and continuous lines correspond to the first and middle synapse in our 1D line implementation (see Fig. 3b). Each colour corresponds to a different number of excitatory synapses, *N*_E_ (legend). Left: Percentage of influence of the synapse undergoing plasticity (synapse’s own NMDA current contribution) to its plasticity. Right: Percentage of influence from the neighbouring synapses (contribution of neighbouring NMDA currents only, without accounting for the synapse’s own NMDA currents).

However, the shape of the distribution of synaptic currents depended on *σ* (Fig. 3d and Extended Data Fig. 3) such that, for small *σ* (that is, only weak spatial coupling of synapses), synapse-specific NMDA currents and weights were proportional to the presynaptic neuronsʼ firing rates (Extended Data Fig. 3d,f). For larger *σ* (that is, when more distant synapses could affect each other), synapses with low presynaptic firing rates were deleted (Extended Data Fig. 3f), as competitive heterosynaptic plasticity disadvantaged these synapses. Although deleted synapses did not generate synapse-specific NMDA currents (Extended Data Fig. 3d), their synapse-specific co-dependent variable *E* (filtered neighboring NMDA currents) did not vanish, becoming independent of the presynaptic neuron’s firing rate and *σ* (Extended Data Fig. 3e). The transition to competition between synapses happened at *σ* = *σ*_th_ ≈ 0.6 (Fig. 3d and Extended Data Fig. 3c–f), which is at 60% of the distance between two immediately neighboring synapses in our unitary distance formulation, meaning that the transition to competition occurs when any two synapses could interact in a substantial way (Extended Data Fig. 3g), in line with the experimental results^24^ (Fig. 3d, *σ*_fit_; Fig. 2j, green line). For the sake of simplicity, we can thus consider all presynaptic synapses onto a single compartment model to affect each other equally, until we introduce dendritic compartments further below.

For a fixed *σ*, the setpoint for the total NMDA current is determined by the learning rates of the three mechanisms involved in the learning rule: LTP, LTD and heterosynaptic plasticity (equation (1); Methods). This setpoint decreases with the increase in the learning rate of heterosynaptic plasticity (Fig. 3e), being independent of initial excitatory weights (Fig. 3f), and slightly dependent on inhibitory input strength (Fig. 3g) due to its effect on the postsynaptic firing rate (Extended Data Fig. 3a). Collectively, these results highlight the excitatory co-dependent plasticity model’s versatility in incorporating effects of spike times, voltage, distance and temporal activation of neighboring synapses in a stable manner.

### EI balance and firing rate setpoint

**Fig. 4:**
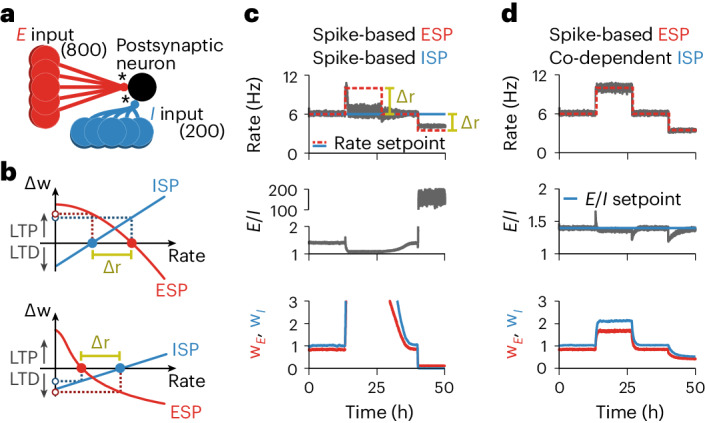
Co-dependent inhibitory synaptic plasticity: EI balance without firing rate setpoint. **a**, Schematic of the simulations used in **c** and **d**. A postsynaptic neuron receives 800 excitatory and 200 inhibitory synapses that undergo plasticity (*). **b**, Schematic of changes in synaptic weight, Δ*w*, as a function of the postsynaptic neuron’s firing rate for spike-based models with stable setpoints. Top, firing rate setpoint from ESP is higher than the one from ISP. Bottom, firing rate setpoint from ISP is higher than the one from ESP. The interval between the setpoint is defined as Δ*r*. **c**, Combination of excitatory^9^ and inhibitory^7^ spike-based rules. Top, firing rate of a postsynaptic neuron receiving excitatory and inhibitory inputs. Red and blue lines indicate the firing rate setpoints imposed by the excitatory^9^ and inhibitory^7^ spike-based learning rules, respectively. The parameters of the learning rules were chosen so that the setpoints coincide during the first and third quarters of the simulation. During the second and fourth quarters of the simulation, the setpoint imposed by the excitatory spike-based learning rule is increased and decreased, respectively. Middle, ratio between excitatory and inhibitory currents. Bottom, average excitatory (red) and inhibitory (blue) synaptic weights of input neurons normalized by their initial value. **d**, Same as **c** for the combination of excitatory spike-based^9^ and co-dependent inhibitory synaptic learning rules. The blue line in the middle panel indicates the balance setpoint imposed by the co-dependent inhibitory synaptic plasticity rule.

The dynamics of traditional spike-based plasticity rules can be approximated by the firing rate of presynaptic and postsynaptic neurons^7,9^. In these types of models, stable postsynaptic activity may be achieved if synaptic weights change toward a firing rate setpoint^7,9^ that controls the dynamics such that excitatory weights increase when the postsynaptic firing rate is lower than the setpoint and decrease otherwise^9^. In the same vein, inhibitory weights decrease for low postsynaptic firing rates (below the setpoint) and increase for high firing rates^7,40^. When both excitatory and inhibitory synapses are plastic (Fig. 4a), the fixed points from both rules must match to avoid a competition between synapses due to the asymmetric nature of excitatory and inhibitory plasticity with firing rate setpoints^41^ (Fig. 4b) that would result in synaptic weights to either diverge or vanish (Fig. 4c). Co-dependent inhibitory plasticity does not have such a problem because there is no firing rate setpoint. Instead, it modifies inhibitory synapses based on an explicit setpoint for excitatory and inhibitory currents (*α* in equation (2)), allowing various stable activity regimes for a postsynaptic neuron while avoiding competition with excitatory plasticity and maintaining a state of balance between excitation and inhibition (Fig. 4d).

### Receptive field plasticity

Sensory neurons have been shown to respond more strongly to some features of stimuli than others, which is thought to facilitate recognition, classification and discrimination of stimuli. The shape of a neuron’s response profile—that is, its receptive field—is a result of its input connectivity^21^. Receptive fields are susceptible to change when an animal learns^42^, with strong evidence supporting receptive field changes as a direct consequence of synaptic plasticity^43^.

**Fig. 5:**
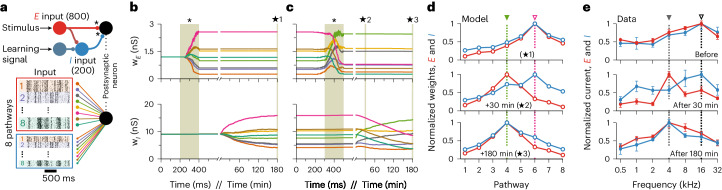
Receptive field plasticity. **a**, External stimulus (for example, sound) activates a set of correlated excitatory and inhibitory afferents (simulated as inhomogeneous Poisson point processes) that feed forward onto a postsynaptic neuron with plastic synapses (*). Eight group pathways, consisting of 100 excitatory and 25 inhibitory afferents each, have correlated spike trains. The responsiveness of inhibitory afferents can be modulated by an additional learning signal. **b**, Timecourse of the mean excitatory (top) and inhibitory (bottom) weights of each group (color coded by groups). During a ‘learning window’, indicated by the shaded area (*), all inhibitory afferents are downregulated. The activation of excitatory input groups (Extended Data Fig. 4a) in the absence of inhibition establishes a receptive field profile. **c**, Continued simulation from **b**. Weights are stable until inhibition is downregulated for a 200-ms window (*), during which the green pathway (4) has the strongest activation (Extended Data Fig. 4b). Consequently, the preferred input pathway switches from 6 (pink) to 4 (green). **d**, Snapshots of the average synaptic weights for the different pathways before (top), immediately after plasticity induction (middle) and at the end of the simulation as indicated by the ⋆ symbols in **b** and **c**. **e**, Experimental data^21^ show receptive field profiles of excitatory and inhibitory inputs before (top) as well as 30 minutes (middle) and 180 minutes (bottom) after pairing of non-preferred tone and nucleus basalis activation. Error bars indicate s.e.m. Experimental data were adapted from ref. ^21^ with permission (we refer to ref. ^21^ for information about sample sizes and statistical analysis).

**Extended Data Fig. 4:**
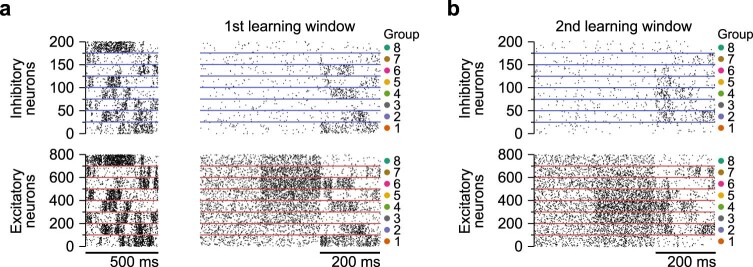
Raster plot of inhibitory (top) and excitatory (bottom) neurons used in the receptive field plasticity simulation (Fig. 5 and Extended Data Fig. 5a). **a**, Input spike patterns before (left) and during (right) learning of the initial receptive field profile (Fig. 5b and Extended Data Fig. 5a,d). **b**, Sequence of input spikes for the modification of the initial receptive field profile (Fig. 5c and Extended Data Fig. 5b,e).

To assess the functional consequence of co-dependent plasticity, we studied its performance in receptive field formation for both excitatory and inhibitory synapses jointly. We simulated a postsynaptic LIF neuron receiving inputs from eight pathways (Methods) that represent, for example, different sound frequencies^21^ (Fig. 5a). In this scenario, inhibitory activity acted as a gating mechanism for excitatory plasticity, by keeping the learning rate at a minimum when inhibitory currents were high^23^ (Fig. 1d). Excitatory input weights could, thus, change only during periods of presynaptic disinhibition—that is, the learning window (Extended Data Fig. 4)—and were otherwise stable (Fig. 5b,c). In our simulations, we initially set all excitatory weights to the same strength. A receptive field profile emerged at excitatory synapses after a period of strong stimulation of pathways during the first learning window. The acquired excitatory receptive profile remained stable (static) after the learning period (Fig. 5b, top). Inhibitory synapses changed on a slower timescale (Fig. 5b, bottom) and, due to the spike timing dependence of co-dependent ISP, developed a co-tuned field with the excitatory receptive field (Fig. 5d, top). Inspired by experimental work^21^, we then briefly activated a non-preferred pathway during a period of disinhibition (Fig. 5c, top), altering the tuning of excitatory weights and making the previously non-preferred pathway ‘preferred’ (Fig. 5d, middle). This change in tuning happened thanks to the Hebbian component of the co-dependent excitatory plasticity rule that induced LTP in the active pathway and the heterosynaptic plasticity component triggering LTD in pathways that were inactive during the learning window, similar to receptive field plasticity reported in mice visual cortex in vivo^19^. As before, inhibitory weights were reshaped by co-dependent ISP to a co-tuned field with the most recent excitatory receptive field (Fig. 5c, bottom), reaching a state of detailed balance, in which excitatory and inhibitory weights are co-tuned based on their input preference^3^ (Fig. 5d, bottom). Plasticity of both excitatory and inhibitory inputs, thus, mimicked results from rat auditory cortex^21^ (Fig. 5e).

**Extended Data Fig. 5:**
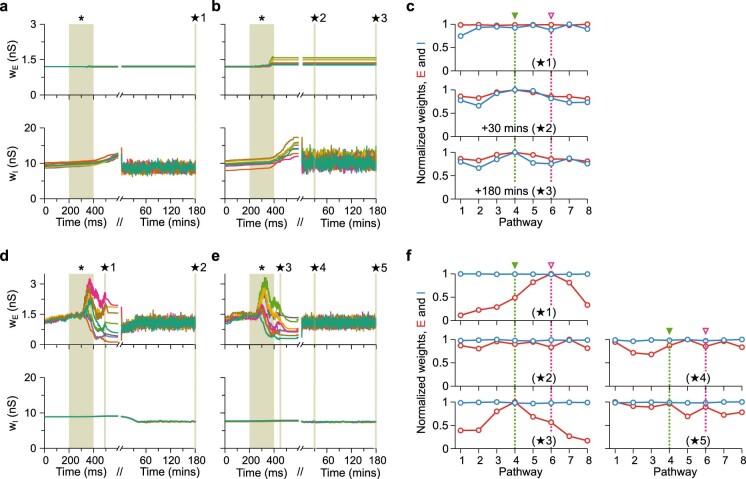
Fast inhibitory plasticity or weak inhibitory control over excitatory plasticity prevents the stable formation of receptive fields. **a** and **b**, Same simulation protocol used in Fig. 5a,b, but with larger learning rate of inhibitory plasticity (increased by 50-fold). Evolution of excitatory (top) and inhibitory (bottom) weights. The shaded area (*) indicates the learning window, when all inhibitory afferents are down-regulated. Excitatory input groups are activated for receptive-field formation during the learning window (Extended Data Fig. 4). **c**, Snapshots of the average synaptic weights for the different pathways at the moments indicated by the ⋆ symbols in panels a and b. **d** and **e**, Same as panels a and b, but with weak inhibitory control over excitatory plasticity rather than fast inhibitory plasticity. **f**, Snapshots of the average synaptic weights for the different pathways at the moments indicated by the ⋆ symbols in panels d and e.

Receptive field formation followed by a reshaping of stimulus-tuned excitation and co-tuned inhibition was successful only when the learning rules were co-dependent (see Supplementary Fig. 3 for a comparison with spike-based and voltage-based models). Moreover, either fast inhibitory plasticity or weak inhibitory control over excitatory plasticity disrupted the formation or stability of receptive fields (Extended Data Fig. 5). When excitatory and inhibitory plasticity operated at similar timescales, inhibitory plasticity prevented excitatory weights to change during disinhibition, because any externally induced decrease in inhibition was quickly compensated for by inhibitory plasticity (Extended Data Fig. 5a–c). With reduced inhibitory control, excitatory weights fluctuated wildly (Extended Data Fig. 5d,e). Although a preferred input signal could be momentarily established, the new preference was soon lost because baseline levels of inhibition were not blocking ongoing excitatory plasticity (Extended Data Fig. 5f).

### Dendritic clustering with single or mixed feature selectivity

**Fig. 6:**
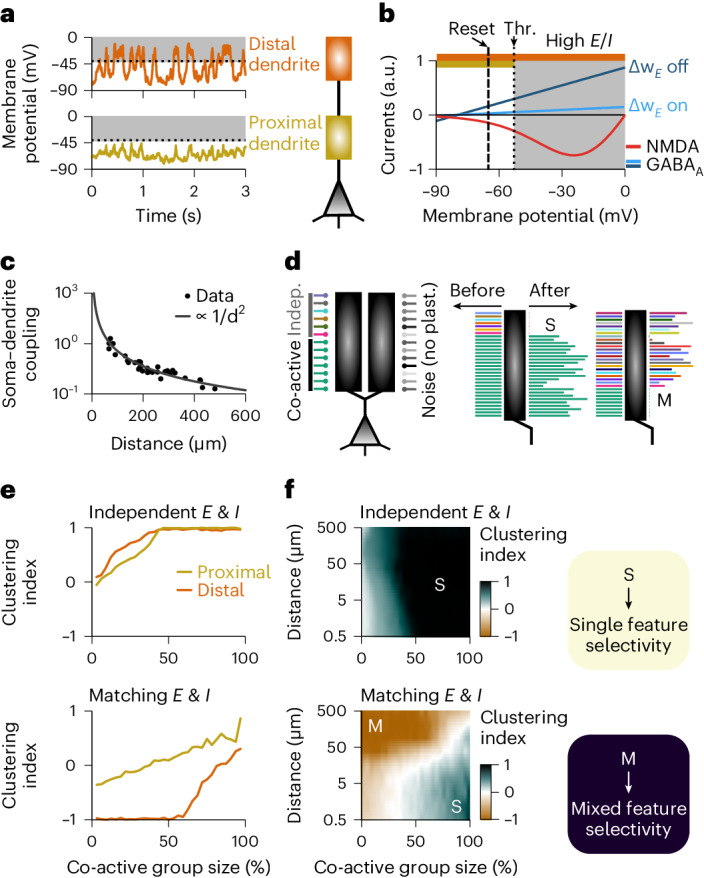
Mixed and single feature selectivity on dendrites depend on presynaptic correlations and distance between soma and dendrite. **a**, Membrane potential fluctuations at distal (top) and proximal (bottom) dendrites during ongoing stimulation. Dashed line shows the spiking threshold at the soma. **b**, NMDA (red) and GABA_A_ (blue) currents as a function of membrane potential. Spiking threshold and reset are indicated by dotted and dashed lines, respectively. **c**, Coupling strength between soma and dendritic branch as a function of electrotonic distance fitted to experimental data adapted from ref. ^45^ with permission. **d**, Schematic of the synaptic organization onto two dendrites (left). In our simulations, both dendrites are connected with the same coupling strength to the soma. The synapses onto one dendrite are plastic for us to assess the effect of co-dependent plasticity, whereas the synapses onto the other dendrite are not plastic to provide background noise mimicking all other dendrites. Each line represents a synapse, with co-active synapses bearing the same color. Examples of clustering of co-active (middle) or independent (right) synapses resulting in single or mixed feature selectivity, respectively, at the level of a single dendrite. Line length indicates synaptic weight in arbitrary units. **e**, Clustering index as a function of the size of the co-active input group for distal (orange) and proximal (yellow) dendrites with independent (top) and matching (bottom) excitatory and inhibitory inputs. Clustering index is equal to 1 (−1) when only co-active (independent) synapses connected onto a given dendritic branch survived and 0 when all synapses survived (Methods). **f**, Clustering index (color coded) as a function of the size of co-active input group (*x* axis) and the distance from the dendrite to the soma (*y* axis) for independent (top) and matching (bottom) excitatory and inhibitory inputs. Dark green indicates single feature selectivity, whereas brown indicates mixed feature selectivity. Indep., independent; no plast., not plastic; Thr., threshold.

**Extended Data Fig. 6:**
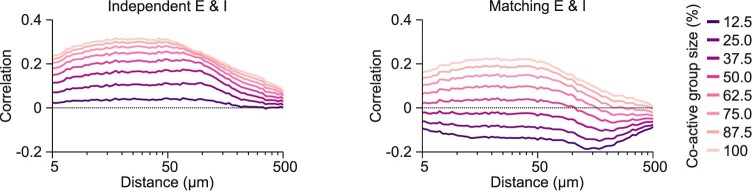
Correlation between postsynaptic spike times and the main input pathway connected to a dendritic compartment. Pearson correlation between filtered postsynaptic spike times (low-pass filter with a 100-ms time constant) and main input pathway as a function of electroctonic distance between the dendritic compartment and the soma. Each colour indicates the co-active group size (see legend) for independent (left) and matching (right) excitatory and inhibitory inputs.

The dendritic tree of neurons is an intricate spatial structure enabling complex neuronal processing that is impossible to achieve in single-compartment neuron models^44^. To assess how our learning rules affected the dendritic organization of synapses, we attached passive dendritic compartments to the soma of our model. Dendritic membrane potentials could be depolarized to values well above the somatic spiking threshold depending on their proximity—that is, electrotonic distance—to the soma (Fig. 6a). These super-threshold membrane potential fluctuations gave rise to larger NMDA and GABA_A_ current fluctuations in distal dendrites (Fig. 6b). Like in the single compartmental models, when excitation and inhibition were unbalanced (that is, when receiving uncorrelated inputs), distal dendrites could undergo fast changes due to the current-induced high learning rates for excitatory plasticity (Fig. 6b, thick red line). However, when currents were balanced (that is, when receiving correlated excitatory and inhibitory inputs), larger inhibitory currents gated excitatory plasticity ‘off’ despite strong excitation (Fig. 6b, thick blue line). Additionally, the larger the distance of a dendrite to the soma and, consequently, weaker passive coupling^45^ (Fig. 6c), the smaller the influence on the initiation of postsynaptic spikes (Extended Data Fig. 6).

**Extended Data Fig. 7:**
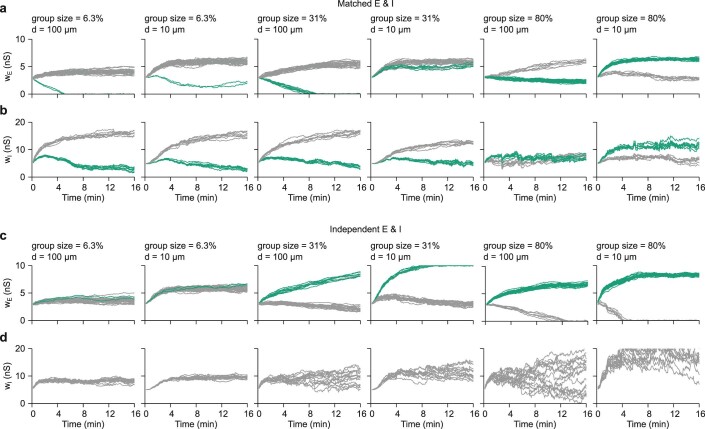
Evolution of synaptic weights connected to dendritic compartments for matched and independent E & I. **a**, Weights of co-active (green) and uncorrelated (grey) excitatory inputs with size of co-active excitatory group and distance of dendritic compartment from the soma indicated by ‘group size’ and ‘d’, respectively. **b**, Weights of co-active (green; same activity pattern as co-active excitatory group) and uncorrelated (grey) inhibitory inputs. Size of the co-active inhibitory group was kept fixed at half of the inhibitory population. **c**, Same as panel a, but when inhibitory inputs are independent of excitatory ones. **d**, Same as in panel b, but with no correlation between excitatory and inhibitory inputs.

Synapses thus developed differently according to the activity of their neighboring inputs and according to somatic proximity (Fig. 6d). When most excitatory inputs onto a dendritic compartment were co-active—that is, originated from the same source (for example, stimulus feature)—their co-active synapses were strengthened, creating a cluster of similarly tuned inputs onto the compartment (Fig. 6d, middle). Uncorrelated, independently active excitatory synapses weakened and eventually faded away (Fig. 6d, middle). In contrast, when more than a certain number of excitatory inputs were independent, co-active synapses decreased in weight and faded, whereas independently active excitatory synapses strengthened (Fig. 6d, right). The number of co-active excitatory synapses necessary for a dendritic compartment to develop single feature tuning varied with somatic proximity and whether excitation and inhibition were matched (Fig. 6e,f and Extended Data Fig. 7). Notably, in the balanced state, substantially more co-active excitatory synapses were necessary to create clusters at distal than at proximal dendrites (Fig. 6e), because only large groups of co-active excitatory synapses could initiate LTP-inducing pre-before-post spike pairs (Extended Data Fig. 6). Thus, single feature or mixed selectivity emerged in our model depending on the branch architecture of the dendritic host structure (Fig. 6f). The resulting connectivity of our simulations, for initially uncorrelated (and, thus, unbalanced) excitatory and inhibitory inputs (Fig. 6f, top), reflects experimental evidence of local dendritic clusters of neighboring excitatory synapses connected onto pyramidal neurons in layer 2/3 of ferretsʼ visual cortex^46^. Moreover, our results were in line with observations in CA3 pyramidal neurons of rats where a larger proportion of clusters of excitatory connections was found in proximal regions of apical dendrites^47^(Fig. 6f, bottom).

### Transient amplification in recurrent spiking networks

**Fig. 7:**
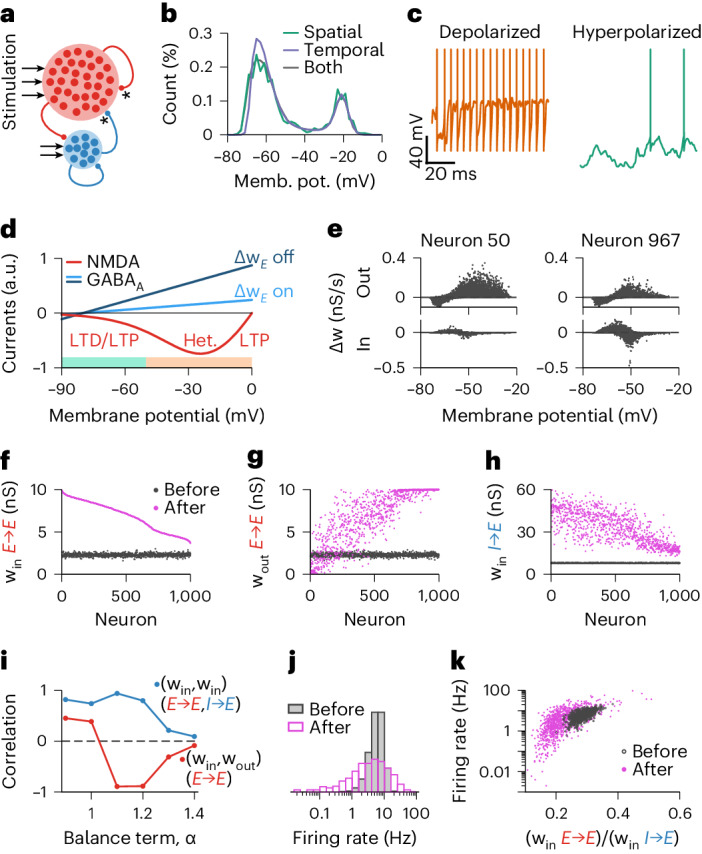
Recurrent network of spiking neurons develops an asymmetric connectivity pattern after learning period with co-dependent synaptic plasticity. **a**, Network of 1,000 excitatory and 250 inhibitory neurons. Connections between excitatory neurons and from inhibitory to excitatory neurons are plastic (indicated by *). **b**, Histogram of the membrane potential during the learning period. Spatial: instantaneous (at a given timestep of the simulation), taking into account all excitatory neurons. Temporal: a single excitatory neuron over 300 s. Both: all excitatory neurons over 300 s. **c**, Examples of membrane potential dynamics during periods of depolarization (left) and hyperpolarization (right). **d**, NMDA (red) and GABA_A_ (blue) currents as a function of membrane potential (as in Fig. 6b), highlighting the possible excitatory weight change during periods of hyperpolarization (green bar) and depolarization (yellow bar). **e**, Sum of excitatory weight changes per second as a function of the membrane potential of the presynaptic (top) and the postsynaptic (bottom) neuron of the connection. Left and right show examples of two distinct neurons of the network. Dots show the amount of change in consecutive 1-s bins given the average membrane potential during that bin. **f**–**h**, Mean excitatory input (**f**) and output (**g**) connection and inhibitory input connection (**h**) received per excitatory neuron before (gray) and after (pink) learning. Neurons are ordered from strongest to weakest mean excitatory input connection after a learning period of 10 h. **i**, Pearson correlation between mean excitatory input and output connections (red) and between mean excitatory and inhibitory input connections (blue) as a function of the balance term used in the co-dependent inhibitory plasticity model. **j**,**k**, Firing rate distribution (**j**) and as a function of the ratio between input excitatory and inhibitory synapses (**k**), before (gray) and after (pink) the learning period. Mem. pot., membrane potential.

**Extended Data Fig. 8:**
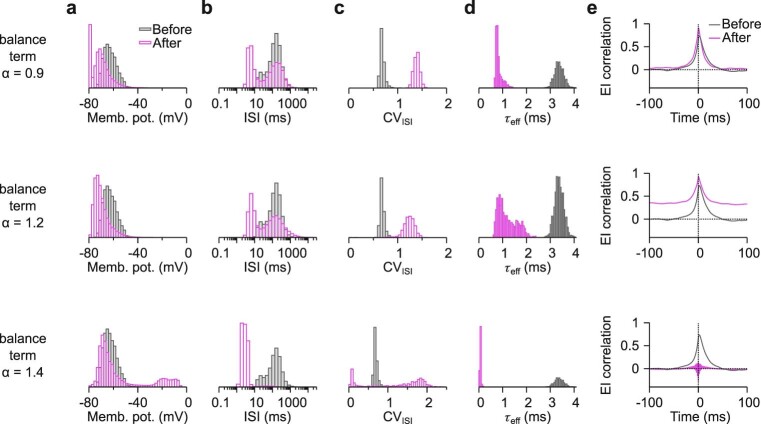
Characterisation of the recurrent network dynamics before and after learning. **a**, Histogram of the membrane potential of all excitatory neurons. **b**, Histogram of the inter-spike-interval (ISI) of all excitatory neurons. **c**, Histogram of the coefficient of variation (standard deviation divided by the mean) of the inter-spike-intervals (from panel b) for all excitatory neurons. **d**, Histogram of the average effective membrane time constant for all excitatory neurons. Effective membrane time constant of a neuron is defined as the neuron’s membrane time constant divided by the neuron’s total conductance. **e**, Pearson correlation between excitatory and inhibitory inputs onto an example excitatory neuron of the network.

**Extended Data Fig. 9:**
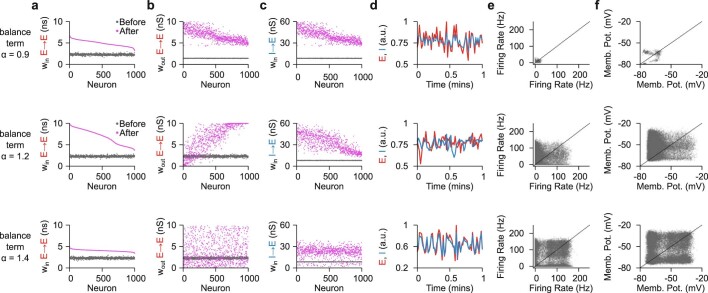
Development of the recurrent connectivity structure for different balancing parameters. **a**, Sum of input excitatory connections onto each excitatory neuron of the network, ordered from the strongest to the weakest connection sum. **b**, Sum of output excitatory connections per excitatory neuron, following the same order from panel a. **c**, Sum of input inhibitory connections onto each excitatory neuron of the network, following the same order from panel a. **d**, Total excitatory (red) and inhibitory (blue) currents onto a given excitatory neuron of the recurrent network during the learning period. **e**, Firing-rate of two excitatory neurons in the recurrent network at different time bins (of size 1 second). **f**, Average membrane potential (calculated in a 1-second time bin) of the two neurons from panel b. Each row shows plots of simulations with a different balancing term, *α* (Eq. (2)). Panels a-c in the middle row (*α* = 1.2) are the same as in Fig. 7f–h.

Up to here, we explored the effects of co-dependent synaptic plasticity in a single postsynaptic neuron. However, recurrent neuronal circuits typically amplify instabilities of any synaptic plasticity rules at play^9,35^. We thus investigated co-dependent plasticity in a recurrent neuronal network of spiking neurons with plastic excitatory-to-excitatory (E-E) and inhibitory-to-excitatory (I-E) synapses (Methods and Fig. 7a). Naive network activity was approximately asynchronous and irregular, with unimodal membrane potential distribution (Extended Data Fig. 8). During learning, neurons began to alternate between hyperpolarized and depolarized states (Fig. 7b,c). Excitatory neurons with longer periods of depolarization developed strong (E-E) output synapses and weak (E-E) input synapses. Vice versa, neurons with longer periods of hyperpolarization developed weak output synapses but strong excitatory input synapses (Fig. 7d,e). The network eventually stabilized in a high conductance state^48^ that was driven mainly by the excitatory current setpoint set by the co-dependent excitatory plasticity model (Extended Data Fig. 8). The final connectivity matrix featured opposing strengths of input and output E-E connections—that is, excitatory neurons with strong (E-E) output synapses developed weak (E-E) input synapses and vice versa (Fig. 7f,g)—with I-E connections that were correlated to the E-E input weights of each neuron (Fig. 7h). Notably, this structure in the learned connectivity matrix depended on the balancing setpoint term of the co-dependent inhibitory plasticity model (Fig. 7i and Extended Data Fig. 9a–c; *α* in equation (2)). For a setpoint *α* = *E* / *I* < 1, strong inhibitory currents effectively matched excitatory inputs, not allowing any weight asymmetry to emerge (Extended Data Fig. 9, top row). For *α* > 1.2, periods of network-wide high and low firing rates due to synchronized hyperpolarized and depolarized states (Extended Data Fig. 9, bottom row) led to symmetric connections. For 1 < *α* < 1.2, a strong asymmetry of weights emerged (Fig. 7i and Extended Data Fig. 9, middle row) that resulted in a wide distribution of baseline firing rates in the same network (Fig. 7j,k), similar to what has been observed in cortical recordings in vivo^49^.

**Fig. 8:**
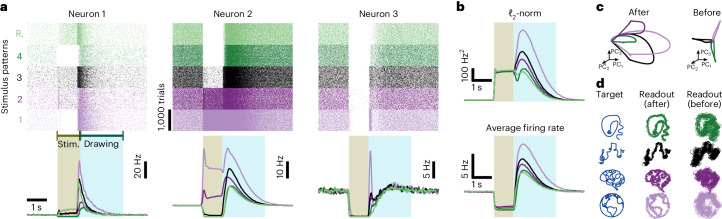
Self-sustained activity and transient amplification in recurrent networks. **a**, Raster plot (top) and mean firing rate (bottom) of three excitatory neurons for five different stimulation patterns. **b**, Norm—that is, *ℓ*_2_-norm of firing rate deviations from baseline (top)—and average firing rate (bottom) of excitatory neurons for the five stimulation patterns in **a**. **c**, First three principal components of the activity of excitatory neurons from the recurrent network after (left) and before (right) learning with co-dependent synaptic plasticity. Each line is the average of 1,000 trials. **d**, Output of two readout neurons trained to draw complex patterns on a 2D plane (‘Target’) using the input from the excitatory neurons from the recurrent network after (left) and before (right) learning with co-dependent synaptic plasticity. Stim., stimulus.

**Extended Data Fig. 10:**
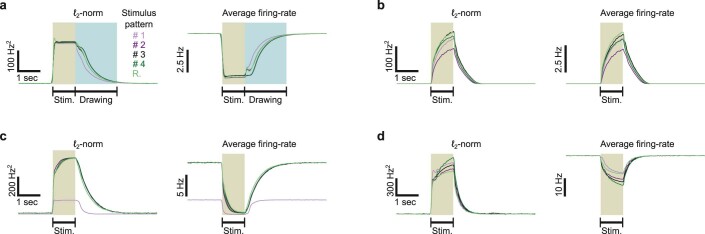
Recurrent network response to external inputs before and after learning with different EI balance set-points. **a**, Dynamics of the naïve network (before learning). Norm, i.e., *ℓ*_2_-norm of the firing-rate deviations from baseline (left), and average firing-rate (right) of excitatory neurons for the five stimulation patterns. Dynamics used in Fig. 8c,d (‘before’). **b**, Same as panel a, but for the naïve network (before learning) without background input (baseline firing-rate is zero for all neurons). **c**, Same as panels a and b, but for a network after learning with *α* = 0.9. **d**, Same as panels a and b, but for a network after learning with *α* = 1.4.

To investigate the network’s response to perturbations, we delivered various stimulus patterns to the network (Methods). Before the external stimulation, network neurons were in a state of self-sustained activity, not receiving any external input. During a 1-s stimulation, used to perturb the network’s dynamics, each of the neurons received external excitatory spikes with a constant, pattern-specific and neuron-specific firing rate (Methods). Randomly selected stimulus patterns (uniformly distributed firing rates) resulted in relatively muted responses (Fig. 8a,b, ‘stimulus R.’) similar to the naive network responses (Extended Data Fig. 10a,b). To identify specific patterns that affected the firing rate dynamics more greatly, we calculated a hypothetical impact of a neuron on the network dynamics, defined as its baseline firing rate (in the self-sustained state) multiplied by its total output weights (according to Fig. 7g,j), giving us a measure of how much a variation in firing rate of a particular neuron would affect the network. To quantify observed network responses, we calculated the *ℓ*_2_-norm of the firing rate deviations from baseline, which takes into account both positive and negative deviations from baseline equally (that is, it is the sum of the square of the individual firing rates minus the baseline; Methods), allowing us to find large transients even when the rate deviations were increased and decreased in equal amounts. The most impactful perturbation stimuli were observed in a network with asymmetric E-E connectivity (Fig. 7f–h). Here, individual neuron responses ranged from small firing rate deflections to large, transient events during or after the delivery of the stimulus that could last several seconds (Fig. 8a,b, ‘stimuli 1–4’), similar to in vivo recordings during sensory activity and movement production^26^ in mammalian systems. The maximum response amplitude resulted from a stimulation pattern in which excitatory neurons with big hypothetical impact and inhibitory neurons with small hypothetical impact received the strong excitatory input currents (Fig. 8a,b, ‘stimulus 1’). Other combinations (for example, shuffling 75% of the ‘stimulus 1’ pattern; Methods) generated intermediate response amplitudes (Fig. 8a,b, ‘stimuli 2–4’). Both naive networks and networks with symmetric connectivity (Fig. 7i, *α* = 0.9 and *α* = 1.4) failed to generate large deviations from baseline after stimulus offset (Extended Data Fig. 10), confirming that co-dependent plasticity shaped the connectivity structure to allow for transient amplification. Finally, the activity of transiently amplified population dynamics could be used to control the activity of a readout network with two output units to draw complex patterns (Fig. 8c,d).

## Discussion

Here we introduce a general framework to describe synaptic plasticity as a function of synapse-specific (presynaptic and postsynaptic) interactions, including the modulatory effects of nearby synapses. We built excitatory and inhibitory plasticity rules according to experimental observations, such that the effect of neighboring synapses could gate, control and even invert the direction of efficacy changes^11–18,24^. Notably, excitatory and inhibitory plasticity rules were constructed such that they strove toward different fixed points (constant levels of excitatory currents for excitatory plasticity and EI balance for inhibitory plasticity), thus collaborating without mutual antagonism.

In our model, inhibition plays an important role in controlling excitatory plasticity, allowing us to make several predictions. First, inhibitory plasticity must be slower than excitatory plasticity. Rapid strengthening of inhibitory weights could compensate for the decreased inhibition during learning periods, effectively blocking excitatory plasticity. Second, inhibitory control over excitatory plasticity has to be relatively strong. That is because the mechanism that allows excitatory weights to quickly reorganize during periods of disinhibition was also responsible for long-term stability of such modifications when inhibitory activity was at baseline. Without strong control, excitatory weights constantly changed due to presynaptic and postsynaptic activity, drifting from the learned weight pattern. Finally, our model also predicts that dendrites on which synaptic contacts of both excitatory and inhibitory presynaptic neurons have correlated activity likely form a connectivity pattern reflecting single feature selectivity. In this scenario, the initial connectivity pattern will determine whether a dendritic region may respond to only a few or many input features, which might, for example, give rise to linear or nonlinear integration of inputs at the soma^44^.

In our model, neighboring excitatory influence on synaptic plasticity was driven by slow, NMDA-like excitatory currents. Consequently, the same pattern of presynaptic and postsynaptic spike times could produce distinct weight dynamics depending on the levels of postsynaptic depolarization (due to an increase in excitatory currents through NMDA channels caused by the release of the magnesium block^50^). However, an increase in excitatory activity can lead to a rise in the amplitude of excitatory currents (thus also eliciting stronger LTP), even without depolarization of the postsynaptic neuron (when, for example, inhibition tightly balances excitation). Postsynaptic membrane potential and presynaptic spike patterns, thus, independently control excitatory plasticity in our model. This is in line with cooperative views on synaptic plasticity^18^ and experimental findings showing that high-frequency stimulation, which usually elicits LTP, produces LTD when NMDA ion channels are blocked^51^. Further experimental data are necessary to disentangle the specific role of excitatory currents and postsynaptic firing frequency in shaping excitatory synaptic plasticity and, thus, unveiling the precise biological form of co-dependent plasticity.

The setpoint dynamics for excitatory currents can be interpreted as a mechanism that normalizes excitatory weights by keeping their total combined weights within a range that guarantees a certain level of excitatory currents, similarly to homeostatic regulation of excitatory bouton size in dendrites^52^. Our rule accomplishes this homeostatic regulation through a local combination of Hebbian LTP and heterosynaptic weakening, similarly to what has been reported in dendrites of visual cortex of mice in vivo^19^. Our results show how such plasticity can develop a stable, balanced network that amplifies particular types of input, generating complex spatiotemporal patterns of activity. These networks developed such that they emulate motor-like outputs for both average and single-trial experiments^26,53^ without specifically being tuned for it. In our simulations, the phenomenon of transient amplification emerged as a result of the network acquiring a stable high conductance state^48^ with asymmetric excitatory–excitatory connectivity. This state was established by an autonomous modification of excitatory weights toward a setpoint for excitatory currents combined with periods of hyperpolarized and depolarized membrane potential. Notably, excitation was balanced by inhibition due to the inhibitory weights self-adjusting toward a regime of precise balance.

Our set of co-dependent synaptic plasticity rules integrates the mathematical formulation of a number of previously proposed rules that rely on spike times^5,7,9^, synaptic current^8,38^ with implicit voltage dependence^6,37^, heterosynaptic weakening^9^ and neighboring synaptic activation^31,38^ in a single theoretical framework. In addition to amplifying correlated input activity by way of controlling the efficacy of a synapse, each of the mechanisms in these previous models may replicate a different facet of learning that was not fully explored with our model and may serve as a starting point for future modifications of the co-dependent plasticity rules that we put forward. For example, spike-based plasticity rules can maintain a set of stable firing rate setpoints^7,9,25^. Rules based on local membrane potentials^6^, on the other hand, are ideal for spatially extended dendritic structure, making it possible to detect localized activity and allowing a spatial redistribution of synaptic weights to improve, for example, associative memory when multiple features are learned by a neural network^37^. Similarly, calcium-influx-related models^8^ are ideal to incorporate information about presynaptic activation, explaining the emergence of binocular matching in dendrites^38^. Neighboring activation models^31^ emulate neurotrophic factors that influence the emergence of clustering of synapses during development.

We unified these disparate approaches in a four-variable model that accounts for the interplay between different synapse types during learning and captures a large range of experimental observations. We focused on only two types of synapses—that is, excitatory-to-excitatory and inhibitory-to-excitatory synapses, in an abstract setting—but the simplicity of our model allows for the adaptation of a larger number of synaptic types, including, for example, modulatory signals present in three-factor learning rules^54^. Faithful modeling of a broader range of influences will require additional experimental work to monitor multi-cell interactions by way of, for example, patterns of excitatory input with glutamate uncaging^55^ or all-optical intervention in vivo^56,57^. Looking at synaptic plasticity from a holistic viewpoint of integrated synaptic machinery, rather than as a set of disconnected mechanisms, may provide a solid basis to understanding learning and memory.

## Methods

### Neuron model

#### Point neuron

In the simulations with a postsynaptic neuron described by a single variable (point neuron), we implemented a LIF neuron with after-hyperpolarization (AHP) current and conductance-based synapses. The postsynaptic neuron’s membrane potential, *u*(*t*), evolved according to a first-order differential equation:

3 $$\begin{array}{rcl}{\tau }_{{{{\rm{m}}}}}\frac{{{\rm{d}}}u(t)}{{{\rm{d}}}t}&=&-[u(t)-{u}_{{{{\rm{rest}}}}}]-{g}_{{{{\rm{AHP}}}}}(t)[u(t)-{E}_{{{{\rm{AHP}}}}}]+R{I}_{{{{\rm{ext}}}}}(t)\\ &&-{g}_{{{{\rm{AMPA}}}}}(t)[u(t)-{E}_{{{{\rm{AMPA}}}}}]-{g}_{{{{{\rm{GABA}}}}}_{{{{\rm{A}}}}}}(t)[u(t)-{E}_{{{{{\rm{GABA}}}}}_{{{{\rm{A}}}}}}]\\ &&-{g}_{{{{\rm{NMDA}}}}}(t){H}_{{{{\rm{NMDA}}}}}(u(t))[u(t)-{E}_{{{{\rm{NMDA}}}}}],\end{array}$$

where *τ*_m_ is the membrane time constant (*τ*_m_ = *RC*; leak resistance × membrane capacitance); *u*_rest_ is the resting membrane potential; *g*_AHP_(*t*) is the conductance of the AHP channel with reversal potential *E*_AHP_; *I*_ext_(*t*) is an external current used to mimic experimental protocols to induce excitatory plasticity; and *g*_*X*_(*t*) and *E*_*X*_ are the conductance and the reversal potential of the synaptic channel *X*, respectively, with *X* = {AMPA, NMDA, GABA_A_}. Excitatory NMDA channels were implemented with a nonlinear function of the membrane potential, caused by a Mg^2+^ block, whose effect was simulated by the function:

4 $${H}_{{{{\rm{NMDA}}}}}(u)={\left(1+{a}_{{{{\rm{NMDA}}}}}\exp [{b}_{{{{\rm{NMDA}}}}}(u-{E}_{{{{\rm{NMDA}}}}})]\right)}^{-1},$$

where *a*_NMDA_ and *b*_NMDA_ are parameters^50^. The AHP conductance was modeled as:

5 $$\frac{{{\rm{d}}}{g}_{{{{\rm{AHP}}}}}(t)}{{{\rm{d}}}t}=-\frac{{g}_{{{{\rm{AHP}}}}}(t)}{{\tau }_{{{{\rm{AHP}}}}}}+{A}_{{{{\rm{AHP}}}}}{S}_{{{{\rm{post}}}}}(t),$$

where *τ*_AHP_ is the characteristic time of the AHP channel; *A*_AHP_ is the amplitude of increase in conductance due to a single postsynaptic spike; and *S*_post_(*t*) is the spike train of the postsynaptic neuron:

6 $${S}_{{{{\rm{post}}}}}(t)=\mathop{\sum}\limits_{k}\delta (t-{t}_{k,{{{\rm{post}}}}}^{* }),$$

where ${t}_{k,{{{\rm{post}}}}}^{* }$ is the time of the k*th* spike of the postsynaptic neuron, and *δ*( ⋅ ) is the Dirac’s delta. The synaptic conductance was modeled as:

7 $$\frac{{{\rm{d}}}{g}_{X}(t)}{{{\rm{d}}}t}=-\frac{{g}_{X}(t)}{{\tau }_{X}}+\mathop{\sum}\limits_{j\in X}{w}_{j}(t){S}_{j}(t),$$

where *τ*_*X*_ is the characteristic time of the neuroreceptor *X*. The sum on the right-hand side of equation (7) corresponds to presynaptic spike trains weighted by the synaptic strength *w*_*j*_(*t*). The presynaptic spike train of neuron *j* was modeled as:

8 $${S}_{j}(t)=\mathop{\sum}\limits_{k}\delta \left(t-{t}_{k,j}^{* }\right),$$

where ${t}_{k,\;j}^{* }$ is the time of the *k*th spike of neuron *j*. The postsynaptic neuron elicited an action potential whenever the membrane potential crossed a spiking threshold from below. We simulated two types of threshold: fixed or adaptive.

- **Fixed spiking threshold.** A fixed spiking threshold was implemented as a parameter, *u*_th_. When the postsynaptic neuron’s membrane potential crossed *u*_th_ from below, a spike was generated, and the postsynaptic neuron’s membrane potential was instantaneously reset to *u*_reset_ and then clamped at this value for the duration of the refractory period, *τ*_ref_. All simulations with a single postsynaptic neuron were implemented with a fixed spiking threshold (Figs. 2–6, Extended Data Figs. 2, 3 and 5–7 and Supplementary Figs. 3 and 4), except the simulations in which the action potential was explicitly implemented (Extended Data Fig. 2c,g,k and Supplementary Figs. 2 and 3d; details in the Supplementary Modeling Note).
- **Adapting spiking threshold.** For the simulations of the recurrent network, we used an adapting spiking threshold, *u*_th_(*t*). When the postsynaptic neuron’s membrane potential crossed *u*_th_(*t*) from below, a spike was generated, and the postsynaptic neuron’s membrane potential was instantaneously reset to *u*_reset_ without any additional clamping of the membrane potential (the refractory period that results from the adapting threshold is calculated below). Upon spike, the adapting spiking threshold, *u*_th_(*t*), was instantaneously set to ${u}_{{{{\rm{th}}}}}^{* }$, decaying back to its baseline according to:
  9 $${\tau}_{{{{\rm{th}}}}}\frac{{{\rm{d}}}{u}_{{{{\rm{th}}}}}(t)}{{{\rm{d}}}t}=-{u}_{{{{\rm{th}}}}}(t)+{u}_{{{{\rm{th}}}}}^{0},$$
  where *τ*_th_ is the decaying time for the spiking threshold variable, and ${u}_{{{{\rm{th}}}}}^{0}$ is the baseline for spike generation. The maximum depolarization of the membrane potential is linked to the reversal potential of NMDA, and, thus, the absolute refractory period can be calculated as:
  10 $${\tau }_{{{{\rm{ref}}}}}={\tau }_{{{{\rm{th}}}}}\ln \left(\frac{{u}_{{{{\rm{th}}}}}^{* }-{u}_{{{{\rm{th}}}}}^{0}}{{E}_{{{{\rm{NMDA}}}}}-{u}_{{{{\rm{th}}}}}^{0}}\right),$$
  which is the time the adapting threshold takes to decay to the same value as the reversal potential of the NMDA channels.

#### Two-layer neuron

The two-layer neuron was simulated as a compartmental model with a spiking soma that receives input from *N*_B_ dendritic branches. The soma was modeled as a LIF neuron and the dendrite as a leaky integrator (without generation of action potentials). Somatic membrane potential evolved according to:

11 $$\begin{array}{rcl}{\tau }_{{{{\rm{m}}}}}\frac{{{\rm{d}}}{u}_{{{{\rm{soma}}}}}(t)}{{{\rm{d}}}t}&=&-[{u}_{{{{\rm{soma}}}}}(t)-{u}_{{{{\rm{rest}}}}}]-{g}_{{{{\rm{AHP}}}}}(t)[{u}_{{{{\rm{soma}}}}}(t)-{E}_{{{{\rm{AHP}}}}}]\\ &&-\mathop{\sum }\limits_{i=1}^{{N}_{{{{\rm{B}}}}}}{J}_{i}[{u}_{{{{\rm{soma}}}}}(t)-{u}_{i}(t)].\end{array}$$

The soma of the two-layer neuron was similar to the point neuron (equation (3)); however, synaptic currents were injected on the dendritic tree, which interacted with the soma passively through the last term on the right-hand side of equation (11), *J*_*i*_ being the conductance that controls the current flow due to connection between the soma and the *i*th dendrite. In equation (11), *u*_*i*_(*t*) is the membrane potential of the dendritic branch *i*. When the somatic membrane potential, *u*_soma_(*t*), crossed the threshold, *u*_th_, from below, the postsynaptic neuron generated an action potential, being instantaneously reset to *u*_reset_ and then clamped at this value for the duration of the refractory period, *τ*_ref_.

Dendritic compartments received presynaptic inputs as well as a sink current from the soma. The membrane potential of the *i*th branch, *u*_*i*_(*t*), evolved according to the following differential equation:

12 $$\begin{array}{rcl}{\tau }_{{{{\rm{m}}}}}\frac{{{\rm{d}}}{u}_{i}(t)}{{{\rm{d}}}t}&=&-[{u}_{i}(t)-{u}_{{{{\rm{rest}}}}}]-{J}_{i}[{u}_{i}(t)-{u}_{{{{\rm{soma}}}}}(t)]\\ &&-{g}_{{{{\rm{AMPA}}}},i}(t)[{u}_{i}(t)-{E}_{{{{\rm{AMPA}}}}}]\\ &&-{g}_{{{{{\rm{GABA}}}}}_{{{{\rm{A}}}}},i}(t)[{u}_{i}(t)-{E}_{{{{{\rm{GABA}}}}}_{{{{\rm{A}}}}}}]\\ &&-{g}_{{{{\rm{NMDA}}}},i}(t){H}_{{{{\rm{NMDA}}}}}({u}_{i}(t))[{u}_{i}(t)-{E}_{{{{\rm{NMDA}}}}}].\end{array}$$

Spikes were not elicited in dendritic compartments, but, due to the gating function *H*_NMDA_(*u*) and the absence of spiking threshold, voltage plateaus occurred naturally when multiple inputs arrived simultaneously on a compartment (Fig. 6a). We simulated two compartments (*N*_B_ = 2) with the same coupling with the soma, *J*_*i*_: one whose synapses changed according to the co-dependent synaptic plasticity model and one with fixed synapses that acted as a noise source.

- **Coupling strength as function of electrotonic distance.** The crucial parameter introduced when including dendritic compartments was the coupling, *J*_*i*_, between soma and the dendritic compartment *i*. Steady changes in membrane potential at the soma are attenuated at dendritic compartments, and this attenuation has been shown to decrease with distance. Without synaptic inputs and steady membrane potential at both soma and dendritic compartments, equations (11) and (12) are equal to zero, which results in:
  13 $${J}_{i}=\frac{{a}_{i}}{1-{a}_{i}},$$
  where *a*_*i*_ is the passive dendritic attenuation of the dendritic compartment *i*,
  14 $${a}_{i}=\frac{{\overline{u}}_{i}-{u}_{{{{\rm{rest}}}}}}{{\overline{u}}_{{{{\rm{soma}}}}}-{u}_{{{{\rm{rest}}}}}},$$
  with ${\overline{u}}_{{{{\rm{soma}}}}}$ being a constant steady state held at the soma and ${\overline{u}}_{i}$ being the resulting steady state at the dendritic compartment *i*. The coupling between soma and the dendritic compartment *i* is a function of distance as follows:
  15 $${J}_{i}={f}_{a}(d)=\frac{{d}_{* }^{2}}{{d}^{2}},$$
  where d_*_ is a parameter that we fitted from experimental data from ref. ^45^ (Fig. 6c). We used this fitted parameter to approximate the distance to the soma in Fig. 6f and Extended Data Figs. 6 and 7 according to the soma–dendrite coupling strength used in our simulations.

### Co-dependent synaptic plasticity model

The co-dependent plasticity model is a function on both spike times and input currents. We first describe how synaptic currents are accounted and then how excitatory and inhibitory plasticity models were implemented. We defined a variable *E*_*j*_(*t*) to represent the process triggered by excitatory currents that influence plasticity at the synapse connecting a presynaptic neuron *j* to the postsynaptic neuron. We considered NMDA currents, which reflect influx of calcium into the postsynaptic cell, as the trigger for biochemical processes that are represented by the state of *E*_*j*_(*t*). Its dynamics are described by the weighted sum (Gaussian envelope) of the synapse-specific filtered NMDA current, ${\widetilde{E}}_{j}(t)$,

16 $${E}_{j}(t)=\mathop{\sum}\limits_{k\in E}{f}_{\Delta x}^{{\;{{\rm{E}}}}}(\;j,k){\widetilde{E}}_{k}(t),$$

where ${f}_{\Delta x}^{{{\;{\rm{E}}}}}(j,k)$ is the function describing the effect of synapse *k* in the plasticity of synapse *j* (based on physical distance considering that both synapses are connected onto the same postsynaptic neuron; details below). The synapse-specific filtered NMDA current dynamics are given by:

17 $${\tau}_{{{{\rm{E}}}}}\frac{{{\rm{d}}}{\widetilde{E}}_{j}(t)}{{{\rm{d}}}t}=-{\widetilde{E}}_{j}(t)-{g}_{{{{\rm{NMDA}}}},j}(t){H}_{{{{\rm{NMDA}}}}}(u(t))\left[u(t)-{E}_{{{{\rm{NMDA}}}}}\right],$$

where *τ*_E_ is the characteristic time of the excitatory trace; *u*(*t*) is the postsynaptic membrane potential (dendritic membrane potential for the two-layer neuron model); and *g*_NMDA,*j*_(*t*) is the conductance of the *j*th excitatory synapse connected onto the postsynaptic neuron, with dynamics given by:

18 $$\frac{{{\rm{d}}}{g}_{{{{\rm{NMDA}}}},j}(t)}{{{\rm{d}}}t}=-\frac{{g}_{{{{\rm{NMDA}}}},j}(t)}{{\tau}_{{{{\rm{NMDA}}}}}}+{w}_{j}(t){S}_{j}(t).$$

Inhibitory inputs contributed to the plasticity model through a variable *I*(*t*). For the inhibitory trace, we used GABA_A_ currents, which reflect influx of chloride, as the trigger of the process described by *I*(*t*). The inhibitory trace evolved as:

19 $${\tau}_{{{{\rm{I}}}}}\frac{{{\rm{d}}}I(t)}{{{\rm{d}}}t}=-I(t)+\mathop{\sum}\limits_{k\in I}{g}_{{{{{\rm{GABA}}}}}_{{{{\rm{A}}}}},k}(t)\left[u(t)-{E}_{{{{{\rm{GABA}}}}}_{{{{\rm{A}}}}}}\right],$$

where *τ*_I_ is the characteristic time of the inhibitory trace, and ${g}_{{{{{\rm{GABA}}}}}_{{{{\rm{A}}}}},k}(t)$ is the conductance of the *k*th inhibitory synapse connected onto the postsynaptic neuron (or dendritic compartment) described as:

20 $$\frac{{{\rm{d}}}{g}_{{{{{\rm{GABA}}}}}_{{{{\rm{A}}}}},k}(t)}{{{\rm{d}}}t}=-\frac{{g}_{{{{{\rm{GABA}}}}}_{{{{\rm{A}}}}},k}(t)}{{\tau }_{{{{{\rm{GABA}}}}}_{{{{\rm{A}}}}}}}+{w}_{k}(t){S}_{k}(t).$$

Notice that both *E*_*j*_(*t*) and *I*(*t*) are in units of voltage because the conductance is unit free in our neuron model implementation (equation (3)).

#### Influence of distance between synapses

To incorporate distance-dependent influence of the activation of a synapse’s neighbors onto excitatory plasticity, we implemented the function ${f}_{\Delta x}^{{{\;{\rm{E}}}}}(i,j)$ in equation (16). For simplicity, we considered that the amplitude of the distance-dependent influence decays with Gaussian-like shaped function of the synapses’ distance:

21 $${f}_{\Delta x}^{{{\;{\rm{E}}}}}(i,j)=\exp \left[-\frac{1}{2}{\left(\frac{\Delta x(i,j)}{\sigma }\right)}^{2}\right]{\left\{\frac{1}{{N}_{{{{\rm{E}}}}}}\mathop{\sum}\limits_{k\in E}\exp \left[-\frac{1}{2}{\left(\frac{\Delta x(i,k)}{\sigma }\right)}^{2}\right]\right\}}^{-1},$$

where *N*_E_ is the number of excitatory synapses; *i* is the index of synapse undergoing plasticity; and *j* is the index of the its neighboring synapse, including *j* = *i* so that the strongest effect is the influx of the excitatory current by the synapse undergoing plasticity. In equation (21), the term Δ*x*(*i*, *j*) is the electrotonic distance between synapses *j* and *i*, and the parameter *σ* is the characteristic distance (that is, standard deviation) of the contribution of excitatory synapses for the variable *E*_*j*_(*t*). The term inside curly brackets on the right-hand side of equation (21) is a normalizing constant.

The sum of the co-dependent variables *E*_*j*_(*t*) for a postsynaptic neuron based on the synapse-specific filtered NMDA currents, ${\widetilde{E}}_{j}(t)$, can be written as:

22 $$\begin{array}{rcl}\mathop{\sum}\limits_{i\in E}{E}_{i}(t)&=&\mathop{\sum}\limits_{i\in E}\mathop{\sum}\limits_{j\in E}{\widetilde{E}}_{j}(t)\exp \left[-\frac{1}{2}{\left(\frac{\Delta x(i,j)}{\sigma }\right)}^{2}\right]{\left\{\frac{1}{{N}_{{{{\rm{E}}}}}}\mathop{\sum}\limits_{k\in E}\exp \left[-\frac{1}{2}{\left(\frac{\Delta x(i,k)}{\sigma }\right)}^{2}\right]\right\}}^{-1}\\ &=&\mathop{\sum}\limits_{j\in E}{\widetilde{E}}_{j}(t)\mathop{\sum}\limits_{i\in E}\exp \left[-\frac{1}{2}{\left(\frac{\Delta x(i,j)}{\sigma }\right)}^{2}\right]{\left\{\frac{1}{{N}_{{{{\rm{E}}}}}}\mathop{\sum}\limits_{k\in E}\exp \left[-\frac{1}{2}{\left(\frac{\Delta x(i,k)}{\sigma }\right)}^{2}\right]\right\}}^{-1}\\ &\approx &{N}_{{{{\rm{E}}}}}\mathop{\sum}\limits_{j\in E}{\widetilde{E}}_{j}(t),\,{{\mbox{for}}}\,\,{N}_{{{{\rm{E}}}}}\gg 1.\end{array}$$

With the normalization used in equation (21), the average of the variable *E*_*j*_(*t*) is approximately equal to the total synapse-specific filtered NMDA currents, ${\widetilde{E}}_{j}(t)$ (equation (16)), which is independent of *σ* for a large number of synapses (*N*_E_ ≫ 1). Notably, for very large *σ* values (*σ* ≫ *N*_E_), all synapses influence each other’s plasticity equally, so that its implementation can be simplified as:

23 $${E}_{j}(t)=\mathop{\sum}\limits_{k\in E}{\widetilde{E}}_{k}(t),\forall j.$$

#### Co-dependent excitatory synaptic plasticity

The co-dependent excitatory synaptic plasticity model is an STDP model regulated by excitatory and inhibitory inputs through *E*_*j*_(*t*) and *I*(*t*). The weight of the *j*th synapse onto the the postsynaptic neuron (or dendritic compartment), *w*_*j*_(*t*), changed according to:

24 $$\begin{array}{rcl}\frac{{{\rm{d}}}{w}_{j}(t)}{{{\rm{d}}}t}&=&{\phi }_{{{{\rm{E}}}}}({E}_{j}(t),I(t);{S}_{j}(t),{S}_{{{{\rm{post}}}}}(t))\\ &=&\left\{\left[{A}_{{{{\rm{LTP}}}}}{x}_{j}^{+}(t){E}_{j}(t)-{A}_{{{{\rm{het}}}}}{y}_{{{{\rm{post}}}}}^{E}(t){\left({E}_{j}(t)\right)}^{2}\right]{S}_{{{{\rm{post}}}}}(t)\right.\\ &&\left.-{A}_{{{{\rm{LTD}}}}}{y}_{{{{\rm{post}}}}}^{-}(t){S}_{j}(t){w}_{j}(t)\right\}\exp \left[-{\left(\frac{I(t)}{{I}^{* }}\right)}^{\gamma }\right],\end{array}$$

where *A*_LTP_, *A*_het_ and *A*_LTD_ are the learning rates of long-term potentiation, heterosynaptic plasticity and long-term depression, respectively. The additional parameter I* defines the level of control that inhibitory activity imposes onto excitatory synapses, with parameter *γ* defining the shape of the control. Variables *S*_post_(*t*) and *S*_*j*_(*t*) represent the postsynaptic and presynaptic spike trains, respectively, as described above for the neuron model (equations (6) and (8)). The trace of the presynaptic spike train is represented by ${x}_{j}^{+}(t)$, and the traces of the postsynaptic spike train (with different timescales) are represented by ${y}_{{{{\rm{post}}}}}^{E}(t)$ and ${y}_{{{{\rm{post}}}}}^{-}(t)$. They evolve in time according to:

25 $$\frac{{{\rm{d}}}{x}_{j}^{+}(t)}{{{\rm{d}}}t}=-\frac{{x}_{j}^{+}(t)}{{\tau }_{+}}+{S}_{j}(t),$$

26 $$\frac{{{\rm{d}}}{y}_{{{{\rm{post}}}}}^{E}(t)}{{{\rm{d}}}t}=-\frac{{y}_{{{{\rm{post}}}}}^{E}(t)}{{\tau}_{y{{{\rm{post}}}}}}+{S}_{{{{\rm{post}}}}}(t),$$

and

27 $$\frac{{{\rm{d}}}{y}_{{{{\rm{post}}}}}^{-}(t)}{{{\rm{d}}}t}=-\frac{{y}_{{{{\rm{post}}}}}^{-}(t)}{{\tau }_{-}}+{S}_{{{{\rm{post}}}}}(t).$$

For values of inhibitory trace larger than a threshold, *I*(*t*) > *I*_th_, we effectively blocked excitatory plasticity to mimic complete shunting of backpropagating action potentials^58^ or additional blocking mechanisms that depend on inhibition^23^. We implemented maximum and minimum allowed values for excitatory weights, ${w}_{\max }^{{{{\rm{E}}}}}=10$ nS and ${w}_{\min }^{{{{\rm{E}}}}}=1{0}^{-5}$ nS, respectively.

#### Co-dependent inhibitory synaptic plasticity

Similar to the excitatory learning rule, the co-dependent inhibitory synaptic plasticity is a function of spike times and synaptic currents. The weight of the *j*th inhibitory synapse onto the postsynaptic neuron (or dendritic compartment), *w*_*j*_(*t*), changed over time according to a differential equation given by:

28 $$\begin{array}{rcl}\frac{{{\rm{d}}}{w}_{j}(t)}{{{\rm{d}}}t}&=&{\phi }_{{{{\rm{I}}}}}({E}_{j}(t),I(t);{S}_{j}(t),{S}_{{{{\rm{post}}}}}(t))\\ &=&{A}_{{{{\rm{ISP}}}}}{E}_{j}(t)\left[{E}_{j}(t)-\alpha I(t)\right]\left[{y}_{{{{\rm{post}}}}}(t){S}_{j}(t)+{x}_{j}(t){S}_{{{{\rm{post}}}}}(t)\right].\end{array}$$

Parameters *A*_ISP_ and *α* control the learning rate and the balance of excitatory and inhibitory currents, respectively. Variables *x*_*j*_(*t*) and *y*_post_(*t*) are traces of presynaptic and postsynaptic spike trains, respectively, that create a symmetric STDP-like curve, with dynamics given by:

29 $$\frac{{{\rm{d}}}{y}_{{{{\rm{post}}}}}(t)}{{{\rm{d}}}t}=-\frac{{y}_{{{{\rm{post}}}}}(t)}{{\tau}_{{{{\rm{iSTDP}}}}}}+{S}_{{{{\rm{post}}}}}(t)$$

and

30 $$\frac{{{\rm{d}}}{x}_{j}(t)}{{{\rm{d}}}t}=-\frac{{x}_{j}(t)}{{\tau }_{{{{\rm{iSTDP}}}}}}+{S}_{j}(t).$$

The STDP window is characterized by the time constant *τ*_iSTDP_. The variable *E*_*j*_(*t*) is given by equation (23). We implemented maximum and minimum allowed values for inhibitory weights, ${w}_{\max }^{{{{\rm{I}}}}}=70$ nS and ${w}_{\min }^{{{{\rm{I}}}}}=1{0}^{-5}$ nS, respectively.

#### Experimental protocols: Fig. 2b,c,e,f,h–j and Extended Data Fig. 2d–k

We fitted three datasets with the co-dependent excitatory synaptic plasticity model to asses its dependency on voltage—that is, membrane potential (Fig. 2b)—on the frequency of presynaptic and postsynaptic spikes (Fig. 2c) and on the effect of co-induction of LTP at neighboring synapses (Fig. 2h–i).

- **Voltage-dependent STDP protocol**. Following the original experiments^16^, we simulated five presynaptic and five postsynaptic spikes at 50 Hz, with 10 ms between presynaptic and postsynaptic spike times (pre-before-post; Δ*t* = +10 ms), repeated 15 times with an interval of 10 s in between each pairing (Fig. 2b). The more depolarized the membrane potential, the bigger the effect of the NMDA currents, and, therefore, more LTP was induced. We combined three different ways to depolarize the postsynaptic neuron’s membrane potential: strength of synapse, current clamp and backpropagating action potential (see the Supplementary Modeling Note for details). Postsynaptic spike times were directly implemented in the co-dependent plasticity rule—that is, manually setting the spike times in equation (6), spike times that were also used to generate backpropagating action potentials (Supplementary Fig. 1; see the Supplementary Modeling Note for details). We implemented a parameter sweep on these three quantities (see the Supplementary Modeling Note for details), measuring the average depolarization during the pre-before-post interval of the simulation (200-ms interval starting at the first presynaptic spike in each burst). Due to the multiple ways to depolarize the postsynaptic membrane potential, we plotted a region (instead of a single line) in Fig. 2b indicating the possible weight changes for the same depolarization with the different depolarization methods.
- **Frequency-dependent STDP protocol**. Following the protocol from the original experiments^15^, we simulated 60 presynaptic and postsynaptic spikes with either Δ*t* = +10 ms (pre-before-post) or Δ*t* = −10 ms interval (post-before-pre) with firing rates between 0.1 Hz and 50 Hz. In the simulations of the frequency-dependent protocol (Fig. 2c), postsynaptic spikes were induced by the injection of a current pulse, *I*_ext_(*t*) = 3 nA, for the duration of 2 ms. For a smooth curve, we incremented presynaptic and postsynaptic firing rates in steps of 0.1 Hz (500 simulations per pairing in total). The increase in presynaptic firing rate caused a bigger accumulation in NMDA currents, which increased LTP (Extended Data Fig. 2a). In the simulations with extra presynaptic partners (Fig. 2e,f and Extended Data Fig. 2d–k), we calculated the average synaptic change over 10 trials to account for the trial-to-trial variability due to the added external Poisson spike trains.
- **Distance-dependent STDP protocol**. In the simulations of the distance-dependent protocol (Fig. 2h–i), postsynaptic spikes were induced by the injection of a current pulse, *I*_ext_(*t*) = 3 nA, for the duration of 2 ms. We simulated 60 presynaptic spikes with inter-spike interval of 500 ms, each followed by three postsynaptic spikes with inter-spike interval of 20 ms. For Fig. 2h, we varied the interval between the presynaptic spike and the first postsynaptic spike in a three-spike burst, defined as Δ*t*. For Fig. 2i, we simulated the above protocol (pre-before-burst) with an interval Δ*t* = 5 ms (‘strong LTP’) in a given synapse, followed by the same protocol with Δ*t* = 35 ms (‘weak LTP’) in a neighboring synapse (Δ*x* = 3 μm and *σ* = 3.16 μm in equation (21)), varying the interval between the strong and weak LTP inductions. For Fig. 2j, we simulated a similar protocol as the one in Fig. 2i, but we fixed the interval between the strong and weak LTP inductions (90 s) and varied the distance between the synapses.
- **Fitting**. Fitting was done with brute force parameter sweep on four parameters for Fig. 2b,c (each fit with different values): *A*_LTP_, *A*_het_, *A*_LTD_ and *τ*_E_. For Fig. 2h–j, a similar brute force parameter sweep on five parameters was performed: *A*_LTP_, *A*_het_, *A*_LTD_, *τ*_E_ and *σ*, with the three plots having the same set of parameters.

#### Stability

The co-dependent plasticity model has a rich dynamics that involves changes in synaptic weights due to presynaptic and postsynaptic spike times as well as synaptic weight and input currents. In this section, we briefly analyze the fixed points for input currents and synaptic weights for general conditions of inputs and outputs.

Considering each synapse individually, we can write the average change in weights (from equation (24), ignoring inhibitory inputs) as:

31 $$\begin{array}{rcl}{\left\langle \frac{{{\rm{d}}}{w}_{j}(t)}{{{\rm{d}}}t}\right\rangle}_{t}&=&\left\langle {S}_{{{{\rm{post}}}}}(t)\left[{A}_{{{{\rm{LTP}}}}}{x}_{j}^{+}(t){E}_{j}(t)-{A}_{{{{\rm{het}}}}}{y}_{{{{\rm{post}}}}}^{E}(t){({E}_{j}(t))}^{2}\right]\right.\\ &&{\left.-{A}_{{{{\rm{LTD}}}}}{y}_{{{{\rm{post}}}}}^{-}(t){S}_{j}(t){w}_{j}(t)\right\rangle }_{t}\\ \end{array}$$

32 $$\begin{array}{rcl}{\left\langle \frac{{{\rm{d}}}{w}_{j}(t)}{{{\rm{d}}}t}\right\rangle}_{t}&=&{A}_{{{{\rm{LTP}}}}}{\left\langle {x}_{j}^{+}(t){E}_{j}(t){S}_{{{{\rm{post}}}}}(t)\right\rangle }_{t}\\ &&-{A}_{{{{\rm{het}}}}}{\left\langle {S}_{{{{\rm{post}}}}}(t){y}_{{{{\rm{post}}}}}^{E}(t){({E}_{j}(t))}^{2}\right\rangle }_{t}\\ &&-{A}_{{{{\rm{LTD}}}}}{\left\langle {S}_{j}(t){y}_{{{{\rm{post}}}}}^{-}(t){w}_{j}(t)\right\rangle }_{t}\\ \end{array}$$

33 $$\begin{array}{rcl}{\left\langle \frac{{{\rm{d}}}{w}_{j}(t)}{{{\rm{d}}}t}\right\rangle}_{t}&=&{A}_{{{{\rm{LTP}}}}}{\left\langle {x}_{j}^{+}(t){E}_{j}(t){S}_{{{{\rm{post}}}}}(t)\right\rangle }_{t}\\ &&-{A}_{{{{\rm{het}}}}}{\left\langle {S}_{{{{\rm{post}}}}}(t)\right\rangle }_{t}{\left\langle {y}_{{{{\rm{post}}}}}^{E}(t)\right\rangle }_{t}{\left\langle {({E}_{j}(t))}^{2}\right\rangle }_{t}\\ &&-{A}_{{{{\rm{LTD}}}}}{\left\langle {S}_{j}(t)\right\rangle }_{t}{\left\langle {y}_{{{{\rm{post}}}}}^{-}(t)\right\rangle }_{t}{\left\langle {w}_{j}(t)\right\rangle }_{t},\end{array}$$

where 〈⋅〉_*t*_ is the average over a time window bigger than the timescale of the quantities involved. In equation (33), we took into consideration that presynaptic spike times are not influenced by postsynaptic activity, and, thus, the average of the products in the last term on the right-hand side of equation (32) is the equal to the product of the averages. Additionally, we assumed no strong correlations between *E*_*j*_(*t*) and *S*_post_(*t*) due to the small fluctuations of the variable *E*_*j*_(*t*). Correlations between presynaptic and postsynaptic spikes govern the LTP term and, thus, cannot be ignored. They also depend on the neuron model and amount of inhibition a neuron (or compartment) receives. We can conclude from equation (33) that the weights from silent presynaptic neurons will vanish due to the heterosynaptic term. In our model, these weights can vanish only in moments of disinhibition, when the inhibitory control over excitatory plasticity is minimum.

For our analysis, we consider that all neurons of the network have nearly stationary firing rates without strong fluctuations. Therefore, the spike trains can be rewritten as average firing rates:

34 $${\langle {S}_{j}(t)\rangle }_{t}={\nu }_{j},$$

and the traces from the spike trains become:

35 $${\langle {x}_{j}^{+}(t)\rangle }_{t}={\tau }_{+}{\nu }_{j},$$

where *ν*_*j*_ is the average firing rate of neuron *j*. The same is valid for the postsynaptic neuron’s firing rate as well as all other traces.

We consider the outcome of the excitatory plasticity rule when LTD is not present, *A*_LTD_ = 0, which informs us on steady state for excitatory currents as a competition between LTP and heterosynaptic plasticity only. Moreover, we assume that the postsynaptic firing rate, *ν*_post_, is proportional to the total NMDA current:

36 $${\nu }_{{{{\rm{post}}}}}={\nu }^{* }\left(\frac{1}{{E}^{* }}\mathop{\sum}\limits_{j\in E}{\widetilde{E}}_{j}+1\right)-\frac{\langle {\nu }_{{{{\rm{I}}}}}\rangle \langle {w}_{{{{\rm{I}}}}}\rangle }{{w}_{{{{\rm{I}}}}}^{* }},$$

where 〈*ν*_I_〉 and 〈*w*_I_〉 are the population average firing rate and weight of inhibitory afferents, respectively, and *ν* *, *E* * and ${w}_{{{{\rm{I}}}}}^{* }$ are parameters that depend on the neuron model (see the Supplementary Modeling Note for details). In this case, the steady state of the system is given by:

37 $$\begin{array}{rcl}{\left.\mathop{\sum}\limits_{j\in E}{\widetilde{E}}_{j}\right\vert }_{{A}_{{{{\rm{LTD}}}}} = 0}&=&\frac{{E}^{* }}{2}\left(1-\frac{\langle {\nu }_{{{{\rm{I}}}}}\rangle \langle {w}_{{{{\rm{I}}}}}\rangle }{{\nu }^{* }{w}_{{{{\rm{I}}}}}^{* }}\right)\\ &&+\sqrt{{\left[\frac{{E}^{* }}{2}\left(1-\frac{\langle {\nu }_{{{{\rm{I}}}}}\rangle \langle {w}_{{{{\rm{I}}}}}\rangle }{{\nu }^{* }{w}_{{{{\rm{I}}}}}^{* }}\right)\right]}^{2}+\frac{{A}_{{{{\rm{LTP}}}}}\langle {\nu }_{j}\rangle {\tau }_{+}{E}^{* }}{{A}_{{{{\rm{het}}}}}{\tau }_{y{{{\rm{post}}}}}{\nu }^{* }}},\end{array}$$

This is also the maximum value for excitatory currents for when LTD is present, as LTD can only decrease synaptic weights. To arrive in equation (37), we set equation (33) to zero and summed over *j* assuming weak correlations between presynaptic and postsynaptic spikes so that ${\langle {x}_{j}^{+}(t){E}_{j}(t){S}_{{{{\rm{post}}}}}(t)\rangle }_{t}={\langle {x}_{j}^{+}(t)\rangle }_{t}{\langle {E}_{j}(t)\rangle }_{t}{\langle {S}_{{{{\rm{post}}}}}(t)\rangle }_{t}$ (see the Supplementary Modeling Note for details). Notice that this fixed point depends on the presynaptic firing rates and the model parameters. For very low postsynaptic firing rates and weak excitatory weights, assuming two consecutive postsynaptic spikes and, thus, setting ${y}_{{{{\rm{post}}}}}^{{{{\rm{E}}}}}=1$ (rather than an average $\langle {y}_{{{{\rm{post}}}}}^{{{{\rm{E}}}}}\rangle ={\nu }_{{{{\rm{post}}}}}{\tau }_{y{{{\rm{post}}}}}\ll 1$), we find a threshold for which the learning rate of heterosynaptic plasticity induces vanishing of synapses:

38 $${A}_{{{{\rm{het}}}}}=\frac{{A}_{{{{\rm{LTP}}}}}{\nu }^{* }\langle {\nu }_{j}\rangle {\tau }_{+}{\tau }_{y{{{\rm{post}}}}}}{{E}^{* }\left[1+{\tau }_{y{{{\rm{post}}}}}\left({\nu }^{* }-\frac{\langle {\nu }_{{{{\rm{I}}}}}\rangle \langle {w}_{{{{\rm{I}}}}}\rangle }{{w}_{{{{\rm{I}}}}}^{* }}\right)\right]}.$$

For a recurrent network, we can assume that *ν*_*j*_ = *ν*_post_ and thus:

39 $${\overline{E}}_{j}^{\max ,{{{\rm{rec}}}}}=\frac{{A}_{{{{\rm{LTP}}}}}{\tau }_{+}}{{A}_{{{{\rm{het}}}}}{\tau }_{y{{{\rm{post}}}}}},\forall j.$$

Notice that the maximum excitatory current onto a neuron embedded in a recurrent network is independent on firing rate of presynaptic and postsynaptic neurons.

In Fig. 3e–g, we simulated the co-dependent excitatory plasticity model with non-zero *A*_LTP_, *A*_het_ and *A*_LTD_ but without inhibitory control. Each excitatory input was simulated with a constant presynaptic firing rate, 0 < *ν*_*j*_ < 18 Hz, uniformly distributed, while the firing rate of all presynaptic inhibitory neurons was set to 18 Hz (details below). For each corresponding value in the *x* axis of Fig. 3e–g, we simulated 40 trials (one point per trial is plotted). We separated these 40 trials into four combinations of the parameters *σ* and *τ*_E_ (10 trials per parameter set) to confirm the independence of the steady state on these parameters: *σ* = 10 and *τ*_E_ = 1,000 ms; *σ* = 1,000 and *τ*_E_ = 10 ms; and *σ* = 1,000 and *τ*_E_ = 1,000 ms. In Fig. 3e–g, we plotted the theory as equation (37). In Fig. 3e, we plotted the learning rate for which weights may vanish as a dashed vertical line (equation (38)). The parameters from equation (36) were fitted by varying excitatory and inhibitory weights without any plasticity (see the Supplementary Modeling Note for details). Extra postsynaptic spikes were manually added to the plasticity rule implementation (equation (6)) at 1 Hz (Poisson process) to enforce plasticity when excitatory inputs were too weak (compared to inhibitory inputs) to elicit postsynaptic response. To test the effect of input firing rate and LTD with weight dependency, we also simulated a similar protocol (as in Fig. 3e) with different levels of excitatory input (all presynaptic neurons with the same firing rate), LTD and inhibitory gating (Supplementary Fig. 4). These simulations show that the excitatory input levels had minimal effect on the fixed point of excitatory currents.

Applying the same idea to the co-dependent inhibitory synaptic plasticity model, we get the following average dynamics for the *j*th inhibitory weight:

40 $${\left\langle \frac{{{\rm{d}}}{w}_{j}(t)}{{{\rm{d}}}t}\right\rangle }_{t}={\left\langle {A}_{{{{\rm{ISP}}}}}{E}_{j}(t)\left[{E}_{j}(t)-\alpha I(t)\right]\left[{y}_{{{{\rm{post}}}}}(t){S}_{j}(t)+{x}_{j}(t){S}_{{{{\rm{post}}}}}(t)\right]\right\rangle}_{t}$$

41 $${\left\langle \frac{{{\rm{d}}}{w}_{j}(t)}{{{\rm{d}}}t}\right\rangle }_{t}\approx {A}_{{{{\rm{ISP}}}}}\overline{E}\left[\overline{E}-\alpha \overline{I}\right]\left[2{\tau }_{{{{\rm{iSTDP}}}}}{\nu }_{j}{\nu }_{{{{\rm{post}}}}}\right],$$

where $\overline{I}={\langle I(t)\rangle }_{t}$, and *E*_*j*_(*t*) is the same for every inhibitory synapse connected onto the postsynaptic neuron (equation (23)) so that $\overline{E}={\langle {E}_{j}(t)\rangle }_{t},{E}_{j}(t)={E}_{k}(t),\forall j,k$. From equation (41), we can calculate the steady state for the inhibitory learning rule, which results in the balance between excitation and inhibition given by *α*:

42 $$\frac{\overline{E}}{\overline{I}}=\alpha .$$

#### Synaptic changes for simple spike patterns and fixed excitatory and inhibitory input levels

From equation (24) and equation (28), we calculated changes in excitatory and inhibitory synapses for simple spike patterns (Extended Data Fig. 1). We considered fixed excitatory and inhibitory inputs and calculated changes in a given excitatory synapse as:

43 $$\begin{array}{rcl}\Delta {w}_{{{{\rm{E}}}}}&=&\left[{A}_{{{{\rm{LTP}}}}}\exp \left(-\frac{\Delta {t}_{{{{\rm{LTP}}}}}}{{\tau }_{+}}\right)E-{A}_{{{{\rm{het}}}}}\exp \left(-\frac{\Delta {t}_{{{{\rm{het}}}}}}{{\tau }_{{{{\rm{y}}}}}}\right){E}^{2}\right.\\ &&\left.-{A}_{{{{\rm{LTD}}}}}\exp \left(-\frac{\Delta {t}_{{{{\rm{LTD}}}}}}{{\tau }_{-}}\right){w}_{0}\right]\exp \left[-{\left(\frac{I}{{I}^{* }}\right)}^{\gamma }\right],\end{array}$$

where Δ*t*_LTP_ is the interval between presynaptic and postsynaptic spikes (pre-before-post); Δ*t*_het_ is the interval between two consecutive postsynaptic spikes; and Δ*t*_LTD_ is the interval between postsynaptic and presynaptic spikes (post-before-pre). In a similar fashion, we calculated changes at a given inhibitory synapse as:

44 $$\Delta {w}_{{{{\rm{I}}}}}={A}_{{{{\rm{ISP}}}}}E\left(E-\alpha I\right)\exp \left(-\frac{\left\vert \Delta t\right\vert }{{\tau }_{{{{\rm{iSTDP}}}}}}\right),$$

where Δ*t* is the interval between presynaptic and postsynaptic spikes, being positive for pre-before-post and negative for post-before-pre spike patterns.

### Inputs

#### Single output neuron (feedforward network)

Presynaptic spike trains for single neurons were implemented as follows. A spike of a presynaptic neuron *j* occurred in a given timestep of duration Δ*t* with probability *p*_*j*_(*t*) if there was no spike elicited during the refractory period beforehand; ${\tau }_{ref}^{E}$ for excitatory and ${\tau }_{ref}^{I}$ for inhibitory inputs, respectively; and zero otherwise. Different simulation paradigms were defined by the input statistics, which are described below.

- **Constant firing rate**. In Figs. 2e,f, 3 and 4, Extended Data Figs. 2d–k and 3 and Supplementary Figs. 2 and 4, presynaptic neurons fired spikes with a constant probability outside the refractory period. For a constant probability *p*_*j*_(*t*) = *p*_*j*_, the mean firing rate, *ν*_*j*_, was therefore:
  45 $${\nu }_{j}=\frac{1}{\Delta t}{p}_{j}{\left(1-{p}_{j}\right)}^{{\tau }_{ref}^{X}/\Delta t}.$$
  In Figs. 2e,f and 3c,d, Extended Data Figs. 2d–k and 3c,d and Supplementary Figs. 2 and 4, the firing rate for external neurons is indicated in the captions and legends. In Fig. 3c,d (colored points) and Fig. 3e–g, as well as Extended Data Fig. 3, the probability of external excitatory spikes was synapse specific, uniformly distributed: 0 < *p*_*j*_ ⩽ 0.002, whereas the probability of external inhibitory spikes was *p*_*j*_ = 0.002, resulting in 0 < *ν*_*j*_⪅18.1 Hz and *ν*_*j*_ ≈ 18.1 Hz, respectively, considering a timestep Δ*t* = 0.1 ms and refractory periods ${\tau }_{{{{\rm{ref}}}}}^{{{{\rm{E}}}}}=5$ ms and ${\tau }_{{{{\rm{ref}}}}}^{{{{\rm{I}}}}}=2.5$ ms. In Fig. 3c,d (gray points), the probability of external excitatory spikes was *p*_*j*_ = 0.001, whereas the probability of external inhibitory spikes was *p*_*j*_ = 0.002, resulting in *ν*_*j*_ ≈ 9 Hz and *ν*_*j*_ ≈ 18.1 Hz, respectively. In Fig. 4 and Supplementary Fig. 4, the probability of external excitatory and inhibitory spikes was *p*_*j*_ = 5 × 10^−4^ and *p*_*j*_ = 10^−3^ for excitatory and inhibitory afferents, resulting in *ν*_*j*_ ≈ 4.87 Hz and *ν*_*j*_ ≈ 9.75 Hz, respectively.
- **Variable firing rate (pathways)**. In Figs. 5 and 6, Extended Data Figs. 4–7 and Supplementary Fig. 3, presynaptic neurons fired spikes according to an inhomogeneous Poisson process.
  For the receptive field plasticity simulations (Fig. 5, Extended Data Figs. 4 and 5 and Supplementary Fig. 3), we simulated eight input pathways. We defined a pathway as a group of 100 excitatory and 25 inhibitory afferents (spike trains of presynaptic neurons) with two components: a constant background firing rate and a fluctuating firing rate taken from an Ornstein–Uhlenbeck (OU) process as described below. The background firing rate for all 800 excitatory and 200 inhibitory afferents was given by a probability of ${p}_{j}^{{{{\rm{bg}}}}}=2\times 1{0}^{-4}$ for excitatory and ${p}_{j}^{{{{\rm{bg}}}}}=4\times 1{0}^{-4}$ for inhibitory afferents, with respective background firing rates of ${\nu }_{j}^{{{{\rm{bg}}}}}\approx 1.98$ Hz and ${\nu }_{j}^{{{{\rm{bg}}}}}\approx 3.96$ Hz for excitatory and inhibitory presynaptic neurons, respectively, considering a timestep Δ*t* = 0.1 ms and refractory periods of ${\tau }_{ref}^{E}=5$ ms and ${\tau }_{ref}^{I}=2.5$ ms. The fluctuating firing rate of the pathway *μ* was created from an OU process. We used an auxiliary variable, *y*_*μ*_(*t*), that followed stochastic dynamics given by:
  46 $$\frac{{{\rm{d}}}{y}_{\mu}(t)}{{{\rm{d}}}t}=-\frac{{y}_{\mu}(t)}{{\tau }_{{{{\rm{OU}}}}}}+{\xi }_{\mu }(t),$$
  where *τ*_OU_ is the time constant of the OU process, and *ξ*_*μ*_(*t*) is a random variable drawn from a Gaussian distribution with zero mean and unitary standard deviation. The fluctuating probability was then defined as:
  47 $${p}_{j}^{\mu }(t)={p}^{* }{\left[{y}_{\mu }(t)\right]}_{+},$$
  where p* = 0.025 is the amplitude of the fluctuations, and [⋅]_+_ is a rectifying function. The probability of a presynaptic afferent *j* belonging to pathway *μ* to spike due to both background and fluctuating firing rate was given by:
  48 $${p}_{j}(t)={p}_{j}^{\mu }(t)+{p}_{j}^{{{{\rm{bg}}}}}.$$
  In Fig. 5 and Extended Data Fig. 5, we implemented two learning windows: first to learn the initial receptive field profile (Fig. 5b and Extended Data Figs. 5a,d; see Extended Data Fig. 4a) and later to learn the new configuration of the receptive field profile (Fig. 5c and Extended Data Fig. 5b,e; see Extended Data Fig. 4b). During both learning periods, which lasted 700 ms, we set the firing rate of all inhibitory neurons to background firing rate (constant) and the excitatory pathways as follows. During the first 500 ms, we set the probability of all excitatory neurons to spike at background levels (constant). During the last 200 ms, we set the probability of all excitatory neurons in each excitatory pathway as *α*_*μ*_*p*_active_, with *μ* representing the pathway index, 0 ⩽ *α*_*μ*_ ⩽ 1 and *p*_active_ = 0.005. In the first learning period (Extended Data Fig. 4a), we used *α*_6_ = 0.8, *α*_5_ = *α*_7_ = 0.6, *α*_4_ = *α*_8_ = 0.4, *α*_3_ = 0.3, *α*_2_ = 0.2 and *α*_1_ = 0.15. In the second learning period (Extended Data Fig. 4b), we used *α*_4_ = 0.8, *α*_3_ = *α*_5_ = 0.6, *α*_2_ = *α*_6_ = 0.4, *α*_1_ = *α*_7_ = 0.3 and *α*_8_ = 0.2.
  To explore the clustering effect on dendritic compartments in Fig. 6 and Extended Data Figs. 6 and 7, we divided the input spikes in pathways to have co-active or independent presynaptic afferents. We used the same implementation as for the receptive field simulations (described above), but we changed the number of afferents per group in both excitatory and inhibitory presynaptic inputs. A dendritic compartment received 32 excitatory and 16 inhibitory afferents. In Fig. 6e,f and Extended Data Figs. 6 and 7, we used two conditions: independent E & I and matching E & I. In both cases, the number of excitatory afferents following the same fluctuating firing rate was increased from 1 (0% co-active group size) to 32 (100% co-active group size), whereas the remaining excitatory afferents had independent fluctuating firing rates. For independent excitatory and inhibitory inputs (independent E & I), all 16 inhibitory afferents followed independent fluctuating firing rates. For matching excitatory and inhibitory inputs (matching E & I), eight inhibitory afferents followed the same fluctuations in firing rate as the co-active excitatory group (of different sizes), whereas the other eight inhibitory afferents were independent.
  Details of the learning period for Supplementary Fig. 3 can be found in the Supplementary Modeling Note.
- **Recurrent network**. The simulation with the recurrent network had two parts: a learning period with both excitatory and inhibitory plasticity active and a recall period without plasticity mechanisms active.
  - **Learning period**. During the beginning of the learning period of *T* = 10 h, we kept the network receiving a minimum of external input to avoid inactivity. The implementation of the external presynaptic spike trains was as follows. In the beginning of the simulation (first 5 min of simulated time), each excitatory neuron of the network received a spike train from one external source with constant probability *p* = 0.01 (timestep Δ*t* = 0.1 ms) to mimic 100 presynaptic afferents firing at 1 Hz. We decreased the probability to *p* = 0.001 for another 5 min of simulated time and then set it to *p* = 0.0001 for the rest of the simulation.
  - **Recall period**. To elicit transient amplification, we selected specific neurons to receive external input based on the resulted weight matrix and the neurons’ baseline firing rate. Before and after stimulation, no external input was implemented, meaning that the network was in a state of self-sustained activity. During the stimulation period, network neurons were stimulated with presynaptic spikes with a constant firing rate with different amplitudes for each of the five conditions (stimulus patterns) shown in Fig. 8. We ordered excitatory and inhibitory neurons according to their baseline firing rate multiplied by total output weight (from maximum to minimum values), ${\nu }_{j}^{{{{\rm{bg}}}}}\mathop{\sum }\nolimits_{i = 1}^{{N}_{{{{\rm{E}}}}}}{w}_{ij}$ for excitatory neurons and ${\nu }_{j}^{{{{\rm{bg}}}}}\mathop{\sum }\nolimits_{i = 1}^{{N}_{{{{\rm{E}}}}}}{w}_{ij}$ for inhibitory neurons, where *N*_E_ is the total number of excitatory neurons in the recurrent network. We assumed that the bigger the baseline firing rate multiplied by the output weight, the bigger the neuron’s influence on the rest of the network. Considering the order of maximal influence to minimal influence, we used the following patterns of stimulation. For stimulus 1, external firing rates were decreased from ${p}_{j}^{{{{\rm{E}}}}}=0.5$ to ${p}_{j}^{{{{\rm{E}}}}}=0$ for excitatory neurons and increased from ${p}_{j}^{{{{\rm{I}}}}}=0$ to ${p}_{j}^{{{{\rm{I}}}}}=0.25$ for inhibitory neurons. For stimuli 2–4, 25% of excitatory and inhibitory neurons (chosen randomly from a uniform distribution) had the same external input as for stimulus 1, whereas the remaining 75% had a random probability ${p}_{j}^{{{{\rm{E}}}}}=[0,0.5]$ and ${p}_{j}^{{{{\rm{I}}}}}=[0,0.4]$ drawn from a uniform distribution. For stimulus ‘R.’, external firing rates had a random probability ${p}_{j}^{{{{\rm{E}}}}}=[0,0.5]$ and ${p}_{j}^{{{{\rm{I}}}}}=[0,0.4]$ for excitatory and inhibitory neurons, respectively. Notice that in the pattern of stimulation that activated excitatory neurons with large and inhibitory neurons with small impact on the network (stimulus 1), amplification was the largest among the stimulus patterns, and, when the pattern of stimulation was random (stimulus ‘R.’), the resulting network dynamics had minimum amplification (Fig. 8a,b).

### Clustering index for dendritic dynamics (Fig. 6e,f)

We defined the clustering index as:

49 $${c}_{{{{\rm{cluster}}}}}=\frac{\left\langle {w}_{{{{\rm{co}}}}-{{{\rm{active}}}}}\right\rangle -\left\langle {w}_{{{{\rm{independent}}}}}\right\rangle }{\left\langle {w}_{{{{\rm{co}}}}-{{{\rm{active}}}}}\right\rangle +\left\langle {w}_{{{{\rm{independent}}}}}\right\rangle },$$

where $\left\langle {w}_{{{{\rm{co}}}}-{{{\rm{active}}}}}\right\rangle$ is the average of the weights from the co-active excitatory group, and $\left\langle {w}_{{{{\rm{independent}}}}}\right\rangle$ is the average of the weights from all independent groups after learning (see individual weight dynamics in Extended Data Fig. 7). When *c*_cluster_ = 1, the excitatory weights from the co-active group survived after learning and independent ones vanished, whereas, for *c*_cluster_ = −1, the opposite happened. Both co-active and independent groups survived after learning when *c*_cluster_ ≈ 0.

### Training an output to draw complex patterns

To confirm whether the dynamics of the recurrent network were capable of generating rich output dynamics, we connected all excitatory neurons of our recurrent network to two linear readouts, *x*^*t*^ and *y*^*t*^, with discrete timestep *t*, given by:

50 $$\left\{\begin{array}{l}{x}^{t}=\mathop{\sum }\nolimits_{j = 1}^{{N}_{{{{\rm{E}}}}}}{a}_{j}{r}_{j}^{t}+{x}_{0}+{\xi }_{x}^{t}\quad \\ {y}^{t}=\mathop{\sum }\nolimits_{j = 1}^{{N}_{{{{\rm{E}}}}}}{b}_{j}{r}_{j}^{t}+{y}_{0}+{\xi }_{y}^{t},\quad \end{array}\right.$$

where ${\xi }_{x}^{t}$ and ${\xi }_{y}^{t}$ are noise sources taken from a uniform distribution in the interval [−0.02, 0.02]. The readouts represented movement in the horizontal and vertical directions of a two-dimensional (2D) plane. The parameters *a*_*j*_, *b*_*j*_, *x*_0_ and *y*_0_ were optimized to minimize the error in both *x* and *y* coordinates:

51 $$\left\{\begin{array}{l}{e}_{x}^{t}={\left({x}^{t}-{\hat{x}}^{t}\right)}^{2}\quad \\ {e}_{y}^{t}={\left({y}^{t}-{\hat{y}}^{t}\right)}^{2},\quad \end{array}\right.$$

where ${e}_{x}^{t}$ and ${e}_{y}^{t}$ are the errors in the horizontal and vertical directions, respectively, and $-1 < {\hat{x}}^{t} < 1$ and $-1 < {\hat{y}}^{t} < 1$ are the coordinates of one of four complex patterns. To calculate ${r}_{j}^{t}$, we filtered the spike trains of the *j*th neuron with a Gaussian filter with standard deviation *σ*_*r*_ = 10 ms:

52 $${r}_{j}^{t}=\mathop{\sum }\limits_{k=-50}^{50}{\widetilde{r}}_{j}(t+k\Delta T,\Delta T)\frac{\exp \left[-\frac{{(k\Delta T)}^{2}}{2{\sigma }_{r}^{2}}\right]}{\mathop{\sum }\nolimits_{l = -50}^{50}\exp \left[-\frac{{(l\Delta T)}^{2}}{2{\sigma }_{r}^{2}}\right]},$$

where ${\widetilde{r}}_{j}(t,\Delta T)$ is the *j*th neuron’s normalized firing rate deviation from baseline (averaged over trials) in the time bin between *t* and *t* + Δ*T* (Δ*T* = 20 ms):

53 $${\widetilde{r}}_{j}(t,\Delta T)=\frac{\left[\frac{1}{1000}\mathop{\sum }\nolimits_{k = 1}^{1000}\int\nolimits_{t}^{t+\Delta T}{S}_{j}({t}^{{\prime} })d{t}^{{\prime} }\right]-{r}_{j}^{{{{\rm{bg}}}}}}{{r}_{j}^{{{{\rm{bg}}}}}},$$

where ${r}_{j}^{{{{\rm{bg}}}}}$ is the j*th* neuron’s baseline firing rate. The timecourse of the simulation was divided into 88 bins, and the period after the stimulus offset was used to train the output weights to draw four complex patterns for the four different stimuli from Fig. 8 that resulted from distinct patterns of stimulation. Each training epoch (single pattern presentation) was simulated with the average firing rate of 1,000 trials and noise. We used the same activity patterns fed to the two readouts, ${r}_{j}^{t}$, to compute the principal components shown in Fig. 8c. Figure 8d shows 10 trajectories for each pattern. We did not perform any benchmark test as this is beyond the scope of this study.

### Spike-based and voltage-based plasticity models

In Fig. 4c, we combined excitatory^9^ and inhibitory^7^ spike-based plasticity rules to show how they can destructively compete when their firing rate setpoints do not match. In Fig. 4d, we combined an excitatory spike-based plasticity rule^9^ with the co-dependent inhibitory synaptic plasticity rule to show how the competition is not present when the plasticity rules dynamics follow fixed points for different quantities—here, ESP imposes a firing rate setpoint while ISP imposes an input currents setpoint. In Extended Data Fig. 2b,c,f,g,j,k, we compared the co-dependent excitatory plasticity rule with spike based^5^ and voltage based^6^ for the frequency-dependent STDP protocol^15^ with additional external inputs. In Supplementary Fig. 3, we implemented spike-based^5,9,10,59^ and voltage-based^6^ models in a receptive field plasticity paradigm. The spike-based and voltage-based plasticity models are described in the Supplementary Modeling Note.

### Simulations and analyses

All simulations were run with Intel Fortran 19.0.1.144. Parameters used in simulations are defined in Supplementary Tables 1–9. Principal component analysis of the recurrent network activity was performed with MATLAB 2020b. Data collection and analysis were not performed blinded to the conditions of the experiments. No data were excluded.

### Reporting summary

Further information on research design is available in the Nature Portfolio Reporting Summary linked to this article.

## Online content

Any methods, additional references, Nature Portfolio reporting summaries, source data, extended data, supplementary information, acknowledgements, peer review information; details of author contributions and competing interests; and statements of data and code availability are available at 10.1038/s41593-024-01597-4.

### Supplementary information

Supplementary Information Supplementary Figs. 1–4, Supplementary Tables 1–9 and Supplementary Modeling Note.

Reporting Summary

## Data Availability

Spike-timing-dependent plasticity data (ref. ^15^ and ref. ^16^) are publicly available from http://plasticity.muhc.mcgill.ca/page8.html.

## Code availability

Relevant code for simulations reported in this study is available at https://github.com/ejagnes/codependent_plasticity.
