# Supplementary material for: Co-dependent excitatory and inhibitory plasticity accounts for quick, stable and long-lasting memories in biological networks
Source: Nat Neurosci. 2024 Mar 20;27(5):964–74. doi: 10.1038/s41593-024-01597-4 (PMC11089004; doi:10.1038/s41593-024-01597-4)
Supplement: Supplementary file 1 — Supplementary Figs. 1–4, Supplementary Tables 1–9 and Supplementary Modeling Note. [file 41593_2024_1597_MOESM1_ESM.pdf]

nature neuroscience

Article

https://doi.org/10.1038/s41593-024-01597-4

Co-dependent excitatory and inhibitory plasticity accounts for quick, stable and long-lasting memories in biological networks

In the format provided by the authors and unedited

Supplementary Information

Supplementary Figures

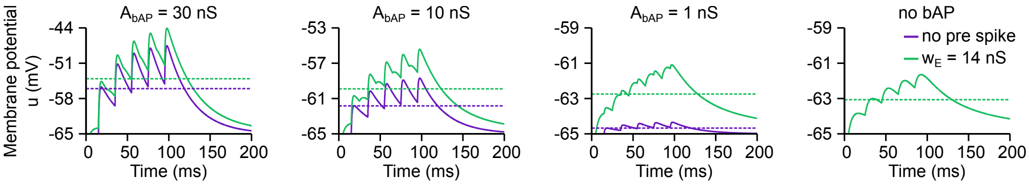

Supplementary Fig. 1 | Postsynaptic neuron's membrane potential dynamics with backpropagating action potential. Time evolution of the postsynaptic neuron's membrane potential when a backpropagating action potential was added to the simulation. We simulated 5 postsynaptic spikes at 50 Hz without presynaptic activation (purple) or followed by 5 presynaptic spikes with a delay Δt = +10 ms (pre-before-post; green). The maximum conductance per postsynaptic spike is indicated on top of each plot (no backpropagating action potential on the plot at the right). Dashed lines indicate the average membrane potential during the 200 ms simulation, which was used to calculate the depolarisation levels for Fig. 2b.

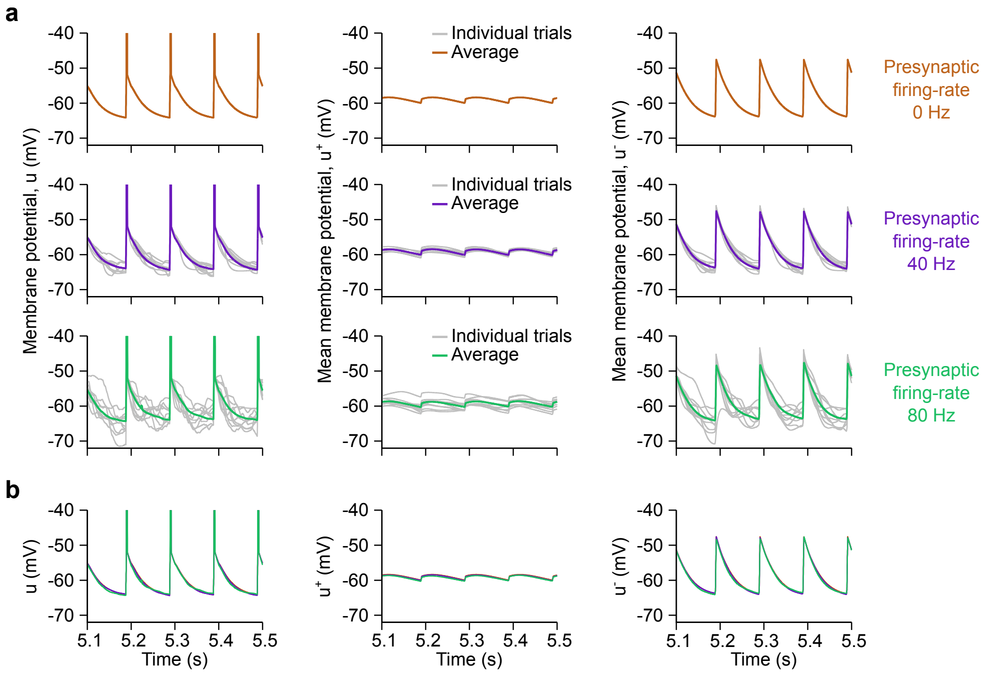

Supplementary Fig. 2 | Postsynaptic neuron's membrane potential dynamics for the frequency-dependent STDP protocol with extra presynaptic inputs (Extended Data Fig. 2g,k) for pre-before-post (Δt = +10 ms) and 10 Hz pairing. a, Dynamics of the membrane potential (left) and its trace with small (middle) and large (right) time constants used in the simulations with the voltage-dependent plasticity rule (Extended Data Fig. 2g,k). Each row corresponds to simulations with different presynaptic firing-rates (indicated on the figure). Gray lines are individual simulations and thick coloured lines are their averages. **b**, Superimposed averages from panel a.

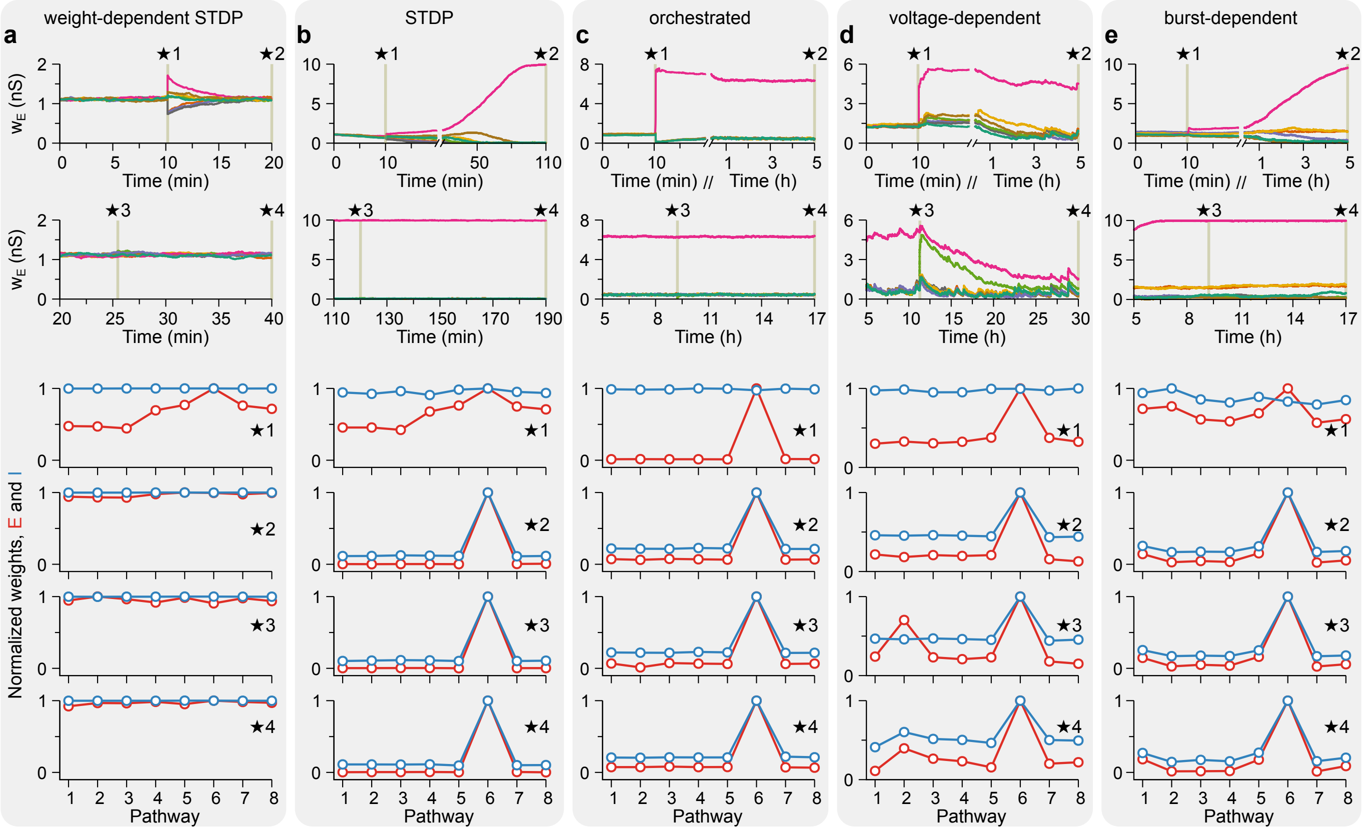

Supplementary Fig. 3 | Receptive field plasticity with alternative plasticity learning rules. a, Time course of the mean excitatory weights for each of the 8 input pathways (top), coloured coded as in Fig. 5. Mean excitatory (red circles) and inhibitory weights (blue circles) at different time points (indicated by the ★ symbol at the top plots). Excitatory plasticity follows a weight-dependent spike-based learning rule while inhibitory plasticity follows a spike-based learning rule. **b**, Same as panel a, with the exception that excitatory plasticity follows a spike-based learning rule without weight dependence. **c**, Same as panel a, but excitatory plasticity follows an orchestrated spike-based learning rule with weight consolidation dynamics. **d**, Same as panel a, but excitatory plasticity follows a voltage-based learning rule. **e**, Same as panel a, but excitatory plasticity follows a burst-dependent spike-based learning rule. All plasticity rules are described in the Supplementary Modelling note.

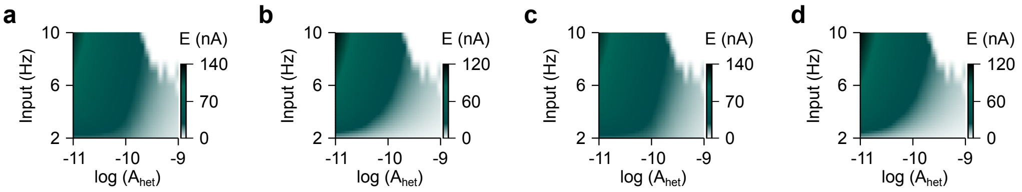

Supplementary Fig. 4 | Final excitatory currents of a postsynaptic neuron as a function of heterosynaptic plasticity and input firing-rate. a, Simulation with weak LTD and weak inhibitory gating. **b**, Simulation with strong LTD and weak inhibitory gating. **c**, Simulation with weak LTD and strong inhibitory gating. **d**, Simulation with strong LTD and strong inhibitory gating. High heterosynaptic learning-rates caused vanishing of weights when inputs have higher firing-rate.

Supplementary Tables

**Parameter**	**Symbol**	**Value**	**Figs.**
Membrane time constant	τ_{m}	30 ms	2-8; ED 2, 3, 5-10; S 1-4
Membrane resistance	R	100 MΩ	2; ED 2; S 3
Membrane capacitance	C	30 nF	not explicitly used
Resting potential	u_{rest}	−65 mV	2-8; ED 2, 3, 5-10; S 1-4
AHP reversal potential	E_{AHP}	−80 mV	2-8; ED 2, 3, 5-10; S 1-4
AMPA reversal potential	E_{AMPA}	0 mV	2-8; ED 2, 3, 5-10; S 1-4
$\mathrm{GABA_A}$ reversal potential	$E_{\mathrm{GABA_A}}$	−80 mV	2-8; ED 2, 3, 5-10; S 1-4
NMDA reversal potential	E_{NMDA}	0 mV	2-8; ED 2, 3, 5-10; S 1-4
AHP time constant	τ_{AHP}	100 ms	2-8; ED 2, 3, 5-10; S 1-4
bAP conductance time constant	τ_{bAP}	1 ms	2b; S 1
AMPA time constant	τ_{AMPA}	5 ms	2-8; ED 2, 3, 5-10; S 1-4
$\mathrm{GABA_A}$ time constant	$\tau_{\mathrm{GABA_A}}$	10 ms	2-8; ED 2, 3, 5-10; S 1-4
NMDA time constant	τ_{NMDA}	150 ms	2-8; ED 2, 3, 5-10; S 1-4
NMDA parameter 1	a_{NMDA}	0.15	2-8; ED 2, 3, 5-10; S 1-4
NMDA parameter 2	b_{NMDA}	$-0.08\ \mathrm{mV}^{-1}$	2-8; ED 2, 3, 5-10; S 1-4
Fixed spiking threshold	u_{th}	−50 mV	2c-j, 3-6; ED 2, 3, 5-7; S 3, 4
		N/A	2b; S 1
AHP conductance amplitude (postsynaptic neuron)	A_{AHP}	N/A	2b; ED 2c,g,k; S 1, 2, 3d
		5 nS	2c,e,f; ED 2a,d,e,h,i
		50 nS	2h-j, 3-6; ED 2,3,5-7; S 2a-c,e, 4
AHP conductance amplitude (excitatory neurons)	A_{AHP}	10 nS	7,8; ED 8-10
AHP conductance amplitude (inhibitory neurons)	A_{AHP}	5 nS	7,8; ED 8-10
bAP conductance amplitude	A_{bAP}	variable	2b; S 1
Spiking threshold reset	u^{*}_{th}	10 mV	7, 8; ED 8-10
Spiking threshold steady-state	u^{0}_{th}	−50 mV	7, 8; ED 8-10
Spiking threshold time constant for excitatory neurons	τ_{th}	10 ms	7, 8; ED 8-10
Spiking threshold time constant for inhibitory neurons	τ_{th}	5 ms	7, 8; ED 8-10
Reset potential	u_{reset}	−60 mV	2c-j, 3-8; ED 2a,b,d-f,h-j, 3, 5-10; S 3a-c,e, 4
		−52 mV	ED 2c,g,k; S 2, 3d
Refractory period	τ_{ref}	5 ms	2c-j,3-6; ED 2a,b,d-f,h-j, 3, 5-7; S 3a-c,e, 4
Action potential peak	u_{AP}	30 mV	ED 2c,g,k; S 2, 3d
Action potential duration	τ_{AP}	2 ms	ED 2c,g,k; S 2, 3d
Voltage attenuation between soma and dendritic compartment	d_{*}	250 μm	6; ED 6, 7
Simulation time step	Δt	0.1 ms	2-8; ED 2-10; S 1-4

Supplementary Table 1 | Simulation parameters for the neuron model. Abbreviations: AHP=After-Hyperpolarisation; bAP=Backpropagating Action Potential; ED=Extended Data; S=Supplementary.

**Parameter**	**Symbol**	**Value**	**Figs.**
Excitatory current filter time constant	τ_E	50 ms	2b,c,e,f; ED 2a,d,e,h,i
(co-dependent ESP)		250 s	2h-j
Inhibitory current filter time constant	τ_I	500 ms	2e,f; ED 2d,h
		N/A	2b,c,h-j; ED 2a,e,i
Excitatory characteristic length (co-dependent ESP)	σ	N/A	2b-f; ED 2a,d,e,h,i
		3.16 μm	2h-i
LTP learning rate (co-dependent ESP)	A_LTP	0.01 mV^{-1}	2b
		2.5×10^{-3} mV^{-1}	2c,e,f; ED 2a,d,e,h,i
		0.5 mV^{-1}	2h-j
LTD learning rate	A_LTD	5×10^{-3}	2b
(co-dependent ESP)		1.2×10^{-2}	2c,e,f; ED 2a,d,e,h,i
		0.02	2h-j
Heterosynaptic plasticity learning rate	A_het	3.5×10^{-4} mV^{-2}	2b
(co-dependent ESP)		10^{-5} mV^{-2}	2c,e,f; ED 2a,d,e,h,i
		0.625 mV^{-2}	2h-j
Inhibitory gating term (co-dependent ESP)	I^*	N/A	2b,c,h-j; ED 2a,e,i;
		10 mV	2e,f; ED 2d,h
Inhibitory gating power (co-dependent ESP)	γ	N/A	2b,c,h-j; ED 2a,e,i
		1	2e,f; ED 2d,h
Presynaptic trace time constant (co-dependent ESP)	τ_+	16.8 ms	2; ED 2a,d,e,h,i
Postsynaptic trace time constant (co-dependent ESP)	$\tau_{y\mathrm{post}}$	100 ms	2; ED 2a,d,e,h,i
Postsynaptic trace time constant (co-dependent ESP)	τ_-	33.7 ms	2; ED 2a,d,e,h,i
Initial excitatory weight	$w_\mathrm{E}(t=0)$	variable	2b
		0.12	2c,e,f,h; ED 2a,d,e,h,i
Initial excitatory weight ('strong LTP' synapse)	$w_\mathrm{E}(t=0)$	0.12	2i,j
Initial excitatory weight ('weak LTP' synapse)	$w_\mathrm{E}(t=0)$	0.018	2i,j
Excitatory weight (fixed)	w_E^*	0.108	2e,f; ED 2d,e,h,i
Inhibitory weight (fixed)	w_I	0.6	2e,f; ED 2d,e,h,i

Supplementary Table 2 | Simulation parameters for the excitatory synaptic plasticity models in Fig. 2 and Extended Data Fig. 2a,d,e,h,i. Abbreviations: LTP=Long-Term Potentiation; LTD=Long-Term Depression; ESP=Excitatory Synaptic Plasticity; ISP=Inhibitory Synaptic Plasticity; ED=Extended Data. Synaptic weights are unit-free in our implementation; to convert synaptic weights to nS, it is necessary to multiply them by the neuron's leak conductance, 10 nS (inverse of membrane resistance).

Parameter	Symbol	Value	Figs.
Excitatory current filter time constant (co-dependent ESP)	τ_{E}	variable	3
		10 ms	ED 3b-f; S 4
Inhibitory current filter time constant (co-dependent ESP)	τ_{I}	N/A ms	3; ED 3b-g
		100 ms	S 4a,b
		2000 ms	S 4c,d
Excitatory characteristic length (co-dependent ESP)	σ	variable	3; ED 3b-g
		N/A	S 4
LTP learning rate (co-dependent ESP)	A_{LTP}	2×10^{-5} mV^{-1}	3; ED 3b-f
		10^{-5} mV^{-1}	S 4
LTD learning rate (co-dependent ESP)	A_{LTD}	$\times 10^{-2}$	3; ED 3b-f
		$\times 10^{-3}$	S 4a,c
		$\times 10^{-2}$	S 4b,d
Heterosynaptic plasticity learning rate (co-dependent ESP)	A_{het}	5×10^{-10} mV^{-2}	3c,d,f,g; ED 3b-f
		variable	3e
		variable	S 4
Inhibitory gating term (co-dependent ESP)	I^{*}	N/A	3; ED 3
		60 mV	S 4
Inhibitory gating power (co-dependent ESP)	γ	N/A	3; ED 3
		1	S 4
Presynaptic trace time constant (co-dependent ESP)	τ_{+}	16.8 ms	3; ED 3b-f; S 4
Postsynaptic trace time constant (co-dependent ESP)	$\tau_{y\mathrm{post}}$	100 ms	3; ED 3b-f; S 4
Postsynaptic trace time constant (co-dependent ESP)	τ_{-}	33.7 ms	3; ED 3b-f; S 4
Initial excitatory weights	$w_{\mathrm{E}}(t = 0)$	0.12	3c-e,g; ED 3b-f; S 4
		variable	3f
		0.5	S 4
Inhibitory weights (fixed)	w_{I}	1	3c-f; ED 3b-f
		variable	3g
		3	S 4

Supplementary Table 3 | Simulation parameters for the excitatory synaptic plasticity models in Fig. 3, Extended Data Fig. 3, and Supplementary Fig. 4. Abbreviations: LTP=Long-Term Potentiation; LTD=Long-Term Depression; ESP=Excitatory Synaptic Plasticity; ISP=Inhibitory Synaptic Plasticity; ED=Extended Data; S=Supplementary. Synaptic weights are unit-free in our implementation; to convert synaptic weights to nS, it is necessary to multiply them by the neuron's leak conductance, 10 nS (inverse of membrane resistance).

Parameter	Symbol	Value	Figs.
Learning rate (spike-based ISP)	A_{ISP}	10^{-3}	4c
LTD term (spike-based ISP)	α	0.228	4c
Pre- and postsynaptic traces time constant (spike-based ISP)	τ_{iSTDP}	20 ms	4c
Neurotransmitter-induced plasticity learning rate (spike-based ESP)	A_{pre}	$\{9.4, 15, 9.4, 5\} \times 10^{-3}$	4c,d
LTP learning rate (spike-based ESP)	A_{LTP}	10^{-3}	4c,d
LTD learning rate (spike-based ESP)	A_{LTD}	4.5×10^{-2}	4c,d
Heterosynaptic plasticity learning rate (spike-based ESP)	A_{het}	0	4c,d
Presynaptic trace time constant (spike-based ESP)	τ_{+}	16.8 ms	4c,d
Postsynaptic trace time constant (spike-based ESP)	τ_{y}	100 ms	4c,d
Postsynaptic trace time constant (spike-based ESP)	τ_{-}	33.7 ms	4c,d
Excitatory current filter time constant (co-dependent ISP)	τ_{E}	100 ms	4d
Inhibitory current filter time constant (co-dependent ISP)	τ_{I}	100 ms	4d
Excitatory characteristic length (co-dependent ISP)	σ	N/A	4d
Learning rate (co-dependent ISP)	A_{ISP}	10^{-8} mV^{-2}	4d
Inhibitory balance set-point (co-dependent ISP)	α	1.4	4d
Pre- and postsynaptic traces time constant (co-dependent ISP)	τ_{iSTDP}	20 ms	4d
Initial excitatory weights	$w_{\mathrm{E}}(t = 0)$	0.12	4c,d
Initial inhibitory weights	$w_{\mathrm{I}}(t = 0)$	0.9	4c,d

Supplementary Table 4 | Simulation parameters for the excitatory synaptic plasticity models in Fig. 4. Abbreviations: LTP=Long-Term Potentiation; LTD=Long-Term Depression; ESP=Excitatory Synaptic Plasticity; ISP=Inhibitory Synaptic Plasticity. Synaptic weights are unit-free in our implementation; to convert synaptic weights to nS, it is necessary to multiply them by the neuron's leak conductance, 10 nS (inverse of membrane resistance).

Parameter	Symbol	Value	Figs.
Excitatory current filter time constant	τ_E	10 ms	5; ED 5
Inhibitory current filter time constant	τ_I	100 ms	5; ED 5
Excitatory characteristic length	σ	N/A	5; ED 5
LTP learning rate (co-dependent ESP)	A_{LTP}	1.7×10^{-4} mV^{-1}	5; ED 5
Heterosynaptic plasticity learning rate (co-dependent ESP)	A_{het}	3×10^{-9} mV^{-2}	5; ED 5
LTD learning rate (co-dependent ESP)	A_{LTD}	0.17	5; ED 5
Inhibitory gating term (co-dependent ESP)	I^*	150 mV	5; ED 5a-c
		400 mV	ED 5d-f
Inhibitory gating power (co-dependent ESP)	γ	3	5; ED 5
Inhibitory threshold (co-dependent ESP)	I_{th}	170 mV	5; ED 5a-c
		10^5 mV	ED 5d-f
Presynaptic trace time constant (co-dependent ESP)	τ_+	16.8 ms	5; ED 5
Postsynaptic trace time constant (co-dependent ESP)	τ_{ypost}	100 ms	5; ED 5
Postsynaptic trace time constant (co-dependent ESP)	τ_-	33.7 ms	5; ED 5
Learning rate (co-dependent ISP)	A_{ISP}	1.5×10^{-9} mV^{-2}	5; ED 5d-f
		$50 \times 1.5 \times 10^{-9}$ mV^{-2}	ED 5a-c
Balance set-point (co-dependent ISP)	α	0.93	5; ED 5
Pre- and postsynaptic traces time constant (co-dependent ISP)	τ_{iSTDP}	20 ms	5; ED 5
Initial excitatory weights	$w_E(t = 0)$	0.12	5; ED 5
Initial inhibitory weights	$w_I(t = 0)$	0.9	5; ED 5
Time constant for Ornstein-Uhlenbeck process	τ_{OU}	50 ms	5; ED 5

Supplementary Table 5 | Simulation parameters for the co-dependent synaptic plasticity model in Fig. 5 and Extended Data Fig. 5. Abbreviations: LTP=Long-Term Potentiation; LTD=Long-Term Depression; ESP=Excitatory Synaptic Plasticity; ISP=Inhibitory Synaptic Plasticity; ED=Extended Data. Synaptic weights are unit-free in our implementation; to convert synaptic weights to nS, it is necessary to multiply them by the neuron's leak conductance, 10 nS (inverse of membrane resistance).

Parameter	Symbol	Value	Figs.
Excitatory current filter time constant	τ_{E}	10 ms	6; ED 7
Inhibitory current filter time constant	τ_{I}	100 ms	6; ED 7
Excitatory characteristic length	σ	N/A	6; ED 7
LTP learning rate (co-dependent ESP)	A_{LTP}	3×10^{-5} mV^{-1}	6; ED 7
Heterosynaptic plasticity learning rate (co-dependent ESP)	A_{het}	6×10^{-9} mV^{-2}	6; ED 7
LTD learning rate (co-dependent ESP)	A_{LTD}	1.5×10^{-3}	6; ED 7
Inhibitory gating term for co-dependent ESP	I^{*}	50 mV	6; ED 7
Inhibitory gating power (co-dependent ESP)	γ	1	6; ED 7
Inhibitory threshold (co-dependent ESP)	I_{th}	N/A	6; ED 7
Presynaptic trace time constant (co-dependent ESP)	τ_{+}	16.8 ms	6; ED 7
Postsynaptic trace time constant (co-dependent ESP)	$\tau_{y\mathrm{post}}$	100 ms	6; ED 7
Postsynaptic trace time constant (co-dependent ESP)	τ_{-}	33.7 ms	6; ED 7
Learning rate (co-dependent ISP)	A_{ISP}	10^{-7} mV^{-2}	6; ED 7
Balance set-point (co-dependent ISP)	α	1.75	6; ED 7
Pre- and postsynaptic traces time constant (co-dependent ISP)	τ_{iSTDP}	20 ms	6; ED 7
Initial excitatory weights	$w_{\mathrm{E}}(t=0)$	0.2	6; ED 7
Initial inhibitory weights	$w_{\mathrm{I}}(t=0)$	0.5	6; ED 7
Time constant for Ornstein-Uhlenbeck process	τ_{OU}	50 ms	6; ED 7

Supplementary Table 6 | Simulation parameters for the co-dependent synaptic plasticity model in Fig. 6 and Extended Data Fig. 7. Abbreviations: LTP=Long-Term Potentiation; LTD=Long-Term Depression; ESP=Excitatory Synaptic Plasticity; ISP=Inhibitory Synaptic Plasticity; ED=Extended Data. Synaptic weights are unit-free in our implementation; to convert synaptic weights to nS, it is necessary to multiply them by the neuron's leak conductance, 10 nS (inverse of membrane resistance).

Parameter	Symbol	Value	Figs.
Excitatory current filter time constant	τ_E	10 ms	7; ED 8,9
Inhibitory current filter time constant	τ_I	100 ms	7; ED 8,9
Excitatory characteristic length	σ	N/A	7; ED 8,9
LTP learning rate (co-dependent ESP)	A_{LTP}	3×10^{-4} mV^{-1}	7b,c,e,f-h,j,k; ED 8 (middle row) ED 9 (middle row)
		7.5×10^{-5} mV^{-1}	ED 8 (top row) ED 9 (top row)
		6×10^{-4} mV^{-1}	ED 8 (bottom row) ED 9 (bottom row)
		8.7×10^{-5} mV^{-1}	7i (for $\alpha = 1$)
		1.2×10^{-4} mV^{-1}	7i (for $\alpha = 1.1$)
		9×10^{-4} mV^{-1}	7i (for $\alpha = 1.3$)
LTD learning rate for co-dependent ESP	A_{LTD}	3×10^{-5}	7; ED 8,9
Heterosynaptic plasticity learning rate for co-dependent ESP	A_{het}	1.5×10^{-8} mV^{-2}	7; ED 8,9
Inhibitory gating term for co-dependent ESP	I^*	200 mV	7; ED 8,9
Inhibitory threshold (co-dependent ESP)	I_{th}	N/A	7; ED 8,9
Presynaptic trace time constant for co-dependent ESP	τ_+	16.8 ms	7; ED 8,9
Postsynaptic trace time constant for co-dependent ESP	τ_{ypost}	100 ms	7; ED 8,9
Postsynaptic trace time constant for co-dependent ESP	τ_-	33.7 ms	7; ED 8,9
Inhibitory plasticity learning rate	A_{ISP}	10^{-8} mV^{-2}	7; ED 8,9
Inhibitory balance set-point	α	1.2	7; ED 8,9
		0.9	7i; ED 8,9
		1.4	7i; ED 8,9
		0.95; 1.1; 1.3	7i
Pre- and postsynaptic traces time constant (co-dependent ISP)	τ_{iSTDP}	20 ms	7; ED 8,9
Initial excitatory-to-excitatory weights; mean ± SD	$w_{EE}(t = 0)$	0.25 ± 0.043	7; ED 8, 9
Initial inhibitory-to-excitatory weights; mean ± SD	$w_{EI}(t = 0)$	0.8 ± 0.08	7; ED 8, 9
Excitatory-to-inhibitory weights (fixed); mean ± SD	w_{IE}	0.3 ± 0.03	7; ED 8,9
Inhibitory-to-inhibitory weights (fixed); mean ± SD	w_{II}	0.3 ± 0.03	7; ED 8,9
External excitatory weights (onto excitatory neurons)	w_{ext}	0.1	7; ED 8,9

Supplementary Table 7 | Simulation parameters for the co-dependent synaptic plasticity model in Fig. 7, as well as Extended Data Figs. 8 and 9. Abbreviations: LTP=Long-Term Potentiation; LTD=Long-Term Depression; ESP=Excitatory Synaptic Plasticity; ISP=Inhibitory Synaptic Plasticity; ED=Extended Data; SD=Standard Deviation. Synaptic weights are unit-free in our implementation; to convert synaptic weights to nS, it is necessary to multiply them by the neuron's leak conductance, 10 nS (inverse of membrane resistance).

Parameter	Symbol	Value	Figs.
Neurotransmitter induced plasticity learning rate (spike-based ESP)	A_{pre}	0	ED 2b,f,j
LTP learning rate (spike-based ESP)	A_{LTP}	10^{-3}	ED 2b,f,j
LTD learning rate (spike-based ESP)	A_{LTD}	4.5×10^{-2}	ED 2b,f,j
Heterosynaptic plasticity learning rate (spike-based ESP)	A_{het}	0	ED 2b,f,j
Presynaptic trace time constant (spike-based ESP)	τ_+	16.8 ms	ED 2b,f,j
Postsynaptic trace time constant (spike-based ESP)	τ_y	100 ms	ED 2b,f,j
Postsynaptic trace time constant (spike-based ESP)	τ_-	33.7 ms	ED 2b,f,j
LTP learning rate (voltage-based ESP)	A_{LTP}	10^{-6} mV^{-2}ms^{-1}	ED 2c,g,k
LTD learning rate (voltage-based ESP)	A_{LTD}	6×10^{-5} mV^{-1}	ED 2c,g,k
Voltage threshold for LTD	θ_-	−52 mV	ED 2c,g,k
Voltage threshold for LTP	θ_+	−55 mV	ED 2c,g,k
Voltage reference for LTD	u^2_{ref}	N/A	ED 2c,g,k
Presynaptic trace time constant (spike-based ESP)	τ_x	15 ms	ED 2c,g,k
Voltage trace time constant for variable u^-	τ_-	10 ms	ED 2c,g,k
Voltage trace time constant for variable u^+	τ_+	150 ms	ED 2c,g,k
Voltage trace time constant for $\overline{\overline{u^-}}$	τ_{u}	N/A	ED 2c,g,k
Initial excitatory weight (plastic synapse)	$w_{\mathrm{E}}(t = 0)$	0.12	ED 2b,c,f,g,j,k
Excitatory weight (fixed)	w^*_{E}	0.108	ED 2b,c,f,g,j,k
Inhibitory weight (fixed)	w_{I}	0.6	ED 2b,c,f,g,j,k

Supplementary Table 8 | Simulation parameters for the spike- and voltage-based plasticity models in Extended Data Fig. 2b,c,f,g,j,k. Abbreviations: LTP=Long-Term Potentiation; LTD=Long-Term Depression; ESP=Excitatory Synaptic Plasticity; ISP=Inhibitory Synaptic Plasticity; ED=Extended Data. Synaptic weights are unit-free in our implementation; to convert synaptic weights to nS, it is necessary to multiply them by the neuron's leak conductance, 10 nS (inverse of membrane resistance).

Parameter	Symbol	Value	Figs.
LTP learning rate	A_{LTP}	10^{-3}	S 3a-c
		1.4×10^{-6}	S 3d
LTD learning rate	A_{LTD}	4.15×10^{-3}	S 3a
		4.98×10^{-4}	S 3b
		10^{-3}	S 3c
		1.25×10^{-5}	S 3d
STDP weight power (Pair-based STDP)	γ_{E}	1	S 3a
		0	S 3b
Heterosynaptic plasticity learning rate	A_{het}	5×10^{-3}	S 3c
Neurotransmitter-induced plasticity learning rate	A_{pre}	2×10^{-5}	S 3c
Burst-plasticity learning rate	A_{burst}	10^{-3}	S 3e
Presynaptic trace time constant	τ_{+}	16.8 ms	S 3a,b
		20 ms	S 3c
		15 ms	S 3d
Presynaptic trace time constant	τ_{pre}	50 ms	S 3e
Postsynaptic trace time constant	τ_{-}	33.7 ms	S 3a,b
		20 ms	S 3c
Postsynaptic trace time constant	τ_{z}	100 ms	S 3c
Consolidation time constant	τ_{w}	100 ms	S 3c
Voltage trace time constant for u^{-}	τ_{-}	10 ms	S 3d
Voltage trace time constant for u^{+}	τ_{+}	150 ms	S 3d
Voltage trace time constant for $\overline{u^{-}}$	τ_{u}	1000 ms	S 3d
Burst trace time constant	τ_{avg}	15 s	S 3e
Burst detector threshold	b_{th}	16 ms	S 3e
Weight consolidation parameter 1	P	20	S 3c
Weight consolidation parameter 2	w_{P}	0.6	S 3c
Voltage threshold for LTD	θ_{-}	−65 mV	S 3d
Voltage threshold for LTP	θ_{+}	−55 mV	S 3d
Voltage reference for LTD	u^{2}_{ref}	60 mV^2	S 3d
Learning rate (spike-based ISP)	A_{ISP}	10^{-5}	S 3a
		10^{-3}	S 3b-e
LTD term (spike-based ISP)	α	0.2	S 3a,b,d,e
		0.04	S 3c
Pre- and postsynaptic traces time constant (spike-based ISP)	τ_{iSTDP}	20 ms	S 3
Initial excitatory weights	$w_{\mathrm{E}}(t=0)$	0.12	S 3
Initial inhibitory weights	$w_{\mathrm{I}}(t=0)$	0.9	S 3

Supplementary Table 9 | Simulation parameters for the synaptic plasticity models in Supplementary Fig. 3. Abbreviations: LTP=Long-Term Potentiation; LTD=Long-Term Depression; STDP=Spike-Timing-Dependent Plasticity; ESP=Excitatory Synaptic Plasticity; ISP=Inhibitory Synaptic Plasticity; S=Supplementary. Synaptic weights are unit-free in our implementation; to convert synaptic weights to nS, it is necessary to multiply them by the neuron's leak conductance, 10 nS (inverse of membrane resistance).

Supplementary Modelling Note

Modifications of the co-dependent plasticity model to approximate previous models. For a proper model comparison, we simplify here the co-dependent plasticity rules to show their relationships to previous models.

co-dependent excitatory plasticity model. For convenience we rewrite Eq. 24 from the main text here, to be modified below,

$$\frac{\mathrm{d}w_j(t)}{\mathrm{d}t} = \Big\{\Big[A_{\mathrm{LTP}} x_j^+(t) E_j(t) - A_{\mathrm{het}} y_{\mathrm{post}}^E(t) (E_j(t))^2\Big] S_{\mathrm{post}}(t) - A_{\mathrm{LTD}} y_{\mathrm{post}}^-(t) S_j(t) w_j(t)\Big\} \exp\left[-\left(\frac{I(t)}{I^*}\right)^{\gamma}\right]. \quad \text{(Supplementary Eq. 1)}$$

First, by setting $E_j(t) = 1$, $A_{\mathrm{het}} = 0$, and $I^* \to \infty$, the equation above becomes the weight-dependent spike-timing-dependent plasticity[1],

$$\frac{\mathrm{d}w_j(t)}{\mathrm{d}t} = A_{\mathrm{LTP}} x_j^+(t) S_{\mathrm{post}}(t) - A_{\mathrm{LTD}} y_{\mathrm{post}}^-(t) S_j(t) w_j(t). \quad \text{(Supplementary Eq. 2)}$$

Second, we can approximate the postsynaptic firing-rate as a quantity proportional to the excitatory inputs, so that $E_j(t) \propto y_{\mathrm{post}}^+(t)$. Together with this approximation, we set $A_{\mathrm{het}} = 0$ and $I^* \to \infty$, arriving at

$$\frac{\mathrm{d}w_j(t)}{\mathrm{d}t} = A_{\mathrm{LTP}} x_j^+(t) y_{\mathrm{post}}^+(t) S_{\mathrm{post}}(t) - A_{\mathrm{LTD}} y_{\mathrm{post}}^-(t) S_j(t) w_j(t), \quad \text{(Supplementary Eq. 3)}$$

which is equivalent to the minimal triplet STDP model from Pfister and Gerstner[2] with weight-dependent LTD.

co-dependent inhibitory plasticity model. We repeat here our analysis for the co-dependent inhibitory plasticity model, which is defined in Eq. 28 from the main text,

$$\frac{\mathrm{d}w_j(t)}{\mathrm{d}t} = A_{\mathrm{ISP}} E_j(t) [E_j(t) - \alpha I(t)] [y_{\mathrm{post}}(t) S_j(t) + x_j(t) S_{\mathrm{post}}(t)]. \quad \text{(Supplementary Eq. 4)}$$

As before, we set $E_j(t) = 1$ for our approximations. Additionally, taking into consideration that the stronger the inhibitory inputs the lower the postsynaptic firing-rate, we assume an inversely proportional relationship between the two, such that $y_{\mathrm{post}}(t) = \theta_{\mathrm{I}}/I(t)$. Changing parameters accordingly and using these two approximations, we can re-write the equation above as

$$\frac{\mathrm{d}w_j(t)}{\mathrm{d}t} = A_{\mathrm{ISP}} \Big[y_{\mathrm{post}}(t) - \alpha\Big] S_j(t) + A_{\mathrm{ISP}} x_j(t) S_{\mathrm{post}}(t) \left[1 - \frac{\theta_{\mathrm{I}}}{y_{\mathrm{post}}(t)}\right]. \quad \text{(Supplementary Eq. 5)}$$

With an additional approximation, in which we set $1 - \theta_{\mathrm{I}}/y_{\mathrm{post}} \approx 1$, we find the symmetric spike-based inhibitory plasticity rule from Vogels *et al.*[3],

$$\frac{\mathrm{d}w_j(t)}{\mathrm{d}t} = A_{\mathrm{ISP}} \Big[y_{\mathrm{post}}(t) - \alpha\Big] S_j(t) + A_{\mathrm{ISP}} x_j(t) S_{\mathrm{post}}(t). \quad \text{(Supplementary Eq. 6)}$$

Postsynaptic firing-rate as a function of excitatory currents and inhibitory inputs. Here, we describe the fitting of the parameters to describe the postsynaptic firing-rate as a function of excitatory currents and inhibitory inputs. We assume a linear relationship between the postsynaptic firing-rate, excitatory currents, and inhibitory inputs (Eq. 36 from main text, rewritten here for convenience),

$$\nu_{\mathrm{post}} = \nu^* \left(\frac{1}{E^*} \sum_{j \in E} \widetilde{E}_j + 1\right) - \frac{\langle \nu_{\mathrm{I}} \rangle \langle w_{\mathrm{I}} \rangle}{w_{\mathrm{I}}^*}. \quad \text{(Supplementary Eq. 7)}$$

To find the parameters ν^*, E^*, and w_{I}^*, we simulated a postsynaptic neuron receiving 800 excitatory connections, each presynaptic neuron with a constant firing-rate drawn from a uniform distribution between 0 and 18 Hz (mean 9 Hz), and 200 inhibitory connections, each inhibitory neuron firing at 18 Hz. All excitatory and inhibitory weights have the same value for a single simulation (without plasticity), and we swept through different weights to fit the parameters (Extended Data Fig. 3a), resulting in a fit with $\nu^* = 21.3$ Hz, $E^* = 3.63$ nA, and $w_{\mathrm{I}}^* = 0.54$.

Fixed-point for the dynamics of the total NMDA currents. Here, we describe the calculation of the fixed-point for the dynamics of the total NMDA currents. Considering no inhibitory control, no LTD ($A_{\mathrm{LTD}} = 0$), and weak correlations,

we arrive at the following dynamics for the average change in excitatory weights,

$$\left\langle \frac{\mathrm{d}w_j(t)}{\mathrm{d}t} \right\rangle_t = A_{\mathrm{LTP}} \nu_j \nu_{\mathrm{post}} \tau_+ E_j - A_{\mathrm{het}} \nu_{\mathrm{post}}^2 \tau_{y\mathrm{post}} E_j^2. \qquad \text{(Supplementary Eq. 8)}$$

To find the fixed-points, we put Supplementary Eq. 8 to zero. To arrive at an analytical expression, we substitute ν_{post} by Supplementary Eq. 7 and sum over j. Finally, we take into consideration that $\sum_{j\in E} E_j = N_{\mathrm{E}} \sum_{j\in E} \widetilde{E}_j$ (Eq. 23 from the main text). With these simplifications and assumptions, we arrive at

$$A_{\mathrm{LTP}} \tau_+ \sum_{j\in E} \nu_j - A_{\mathrm{het}} \tau_{y\mathrm{post}} \left[\nu^* \left(\frac{1}{E^*} \sum_{j\in E} \widetilde{E}_j + 1 \right) - \frac{\langle \nu_{\mathrm{I}} \rangle \langle w_{\mathrm{I}} \rangle}{w_{\mathrm{I}}^*} \right] N_{\mathrm{E}} \sum_{j\in E} \widetilde{E}_j = 0$$

$$\left(\sum_{j\in E} \widetilde{E}_j \right)^2 - E^* \left(1 - \frac{\langle \nu_{\mathrm{I}} \rangle \langle w_{\mathrm{I}} \rangle}{\nu^* w_{\mathrm{I}}^*} \right) \sum_{j\in E} \widetilde{E}_j - \frac{A_{\mathrm{LTP}} \tau_+ E^*}{A_{\mathrm{het}} \tau_{y\mathrm{post}} \nu^*} \frac{1}{N_{\mathrm{E}}} \sum_{j\in E} \nu_j = 0$$

$$\left(\sum_{j\in E} \widetilde{E}_j \right)^2 - E^* \left(1 - \frac{\langle \nu_{\mathrm{I}} \rangle \langle w_{\mathrm{I}} \rangle}{\nu^* w_{\mathrm{I}}^*} \right) \sum_{j\in E} \widetilde{E}_j - \frac{A_{\mathrm{LTP}} \tau_+ E^*}{A_{\mathrm{het}} \tau_{y\mathrm{post}} \nu^*} \langle \nu_j \rangle = 0, \qquad \text{(Supplementary Eq. 9)}$$

which results in

$$\sum_{j\in E} \widetilde{E}_j = \frac{E^*}{2} \left(1 - \frac{\langle \nu_{\mathrm{I}} \rangle \langle w_{\mathrm{I}} \rangle}{\nu^* w_{\mathrm{I}}^*} \right) \pm \sqrt{ \left[\frac{E^*}{2} \left(1 - \frac{\langle \nu_{\mathrm{I}} \rangle \langle w_{\mathrm{I}} \rangle}{\nu^* w_{\mathrm{I}}^*} \right) \right]^2 + \frac{A_{\mathrm{LTP}} \langle \nu_j \rangle \tau_+ E^*}{A_{\mathrm{het}} \tau_y \nu^*} }. \qquad \text{(Supplementary Eq. 10)}$$

Description of spike-based and voltage-based models. Here, we describe spike- and voltage-based plasticity learning rules.

Paired-based spike-timing-dependent plasticity model for excitatory-to-excitatory synapses. A purely spike-based learning rule that governs changes to the j*th* presynaptic weight, $w_j(t)$, according to pre-before-post and post-before-pre spike pairs only, can be written as

$$\frac{\mathrm{d}w_j(t)}{\mathrm{d}t} = A_{\mathrm{LTP}} x_j^+(t) S_{\mathrm{post}}(t) - A_{\mathrm{LTD}} y_{\mathrm{post}}^-(t) S_j(t) \left[w_j(t) \right]^{\gamma_{\mathrm{E}}}, \qquad \text{(Supplementary Eq. 11)}$$

where A_{LTP} and A_{LTD} are the learning rates for LTP and LTD, respectively, $x_j^+(t)$ and $y_{\mathrm{post}}^-(t)$ are low-pass filters of spike trains of pre- and postsynaptic neurons as defined by Eqs. 25 and 27 from the main text, respectively, and $S_{\mathrm{post}}(t)$ and $S_j(t)$ are the post- and presynaptic spike trains as defined by Eqs. 6 and 8 from the main text, respectively. The parameter γ_{E} defines whether the paired-based STDP model is weight-dependent[1] ($\gamma_{\mathrm{E}} = 1$) or not[4] ($\gamma_{\mathrm{E}} = 0$).

Triplet and orchestrated spike-based excitatory plasticity rule. The weight of the j*th* synapse changed according to

$$\begin{aligned} \frac{\mathrm{d}w_j(t)}{\mathrm{d}t} &= A_{\mathrm{pre}} S_j(t) + A_{\mathrm{LTP}} x_j^+(t) y_{\mathrm{post}}^+(t) S_{\mathrm{post}}(t) \\ &\quad - A_{\mathrm{LTD}} y_{\mathrm{post}}^-(t) S_j(t) - A_{\mathrm{het}} [z_{\mathrm{post}}(t)]^3 S_{\mathrm{post}}(t) \left[w_j(t) - \widetilde{w}_j(t) \right], \end{aligned} \qquad \text{(Supplementary Eq. 12)}$$

where A_{pre}, A_{LTP}, A_{LTD}, and A_{het}, are the learning rates for the neurotransmitter-induced plasticity, long-term potentiation, long-term depression, and heterosynaptic plasticity, respectively. The spike trains of post- and presynaptic neurons, $S_{\mathrm{post}}(t)$ and $S_j(t)$ are defined by Eq. 6 and Eq. 8 from the main text, respectively. The traces $x_j^+(t)$, $y_{\mathrm{post}}^+(t)$, and $y_{\mathrm{post}}^-(t)$ followed the same dynamics described in Eq. 25 from the main text for $x_j^+(t)$, and Eq. 27 from the main text for both $y_{\mathrm{post}}^+(t)$ and y_{post}^-, with time constants τ_+, τ_y, and τ_-, respectively. The trace $z_{\mathrm{post}}(t)$ is similar, following the dynamics described by Eq. 27 from the main text with time constant τ_z. Additionally, a consolidation dynamics as described by Zenke *et al.*[5] was implemented when the heterosynaptic term was non-zero, $A_{\mathrm{het}} \neq 0$, through the variable $\widetilde{w}_j(t)$, whose dynamics is defined as

$$\tau_w \frac{\mathrm{d}\widetilde{w}_j}{\mathrm{d}t} = w_j(t) - \widetilde{w}_j(t) - P \widetilde{w}_j(t) \left[\frac{w_{\mathrm{P}}}{2} - \widetilde{w}_j(t) \right] \left[w_{\mathrm{P}} - \widetilde{w}_j(t) \right], \qquad \text{(Supplementary Eq. 13)}$$

where τ_w is the timescale of the consolidation dynamics, while P and w_{P} are parameters controlling the strength and upper stable fixed-point of a double well potential dynamics[5].

To create a stable-fixed point for the postsynaptic firing-rate in Fig. 4, we set $A_{\mathrm{het}} = 0$, and thus the stable firing-rate

set point was given by

$$\nu_0^{\mathrm{E}} = \frac{1}{2A_{\mathrm{LTP}}\tau_+\tau_y}\left(A_{\mathrm{LTD}}\tau_- - \sqrt{A_{\mathrm{LTD}}^2\tau_-^2 - 4A_{\mathrm{pre}}A_{\mathrm{LTP}}\tau_+\tau_y}\right). \quad \text{(Supplementary Eq. 14)}$$

To change the firing-rate set-point during the simulation in Fig. 4c,d, we changed A_{pre}, keeping all the other parameters fixed.

Burst-dependent spike-based excitatory plasticity rule. The dynamics of the weight of the j*th* synapse followed

$$\frac{\mathrm{d}w_j}{\mathrm{d}t}(t) = A_{\mathrm{burst}}\left[S_{\mathrm{burst}}(t) - \frac{y_{\mathrm{burst}}(t)}{y_{\mathrm{event}}(t)}S_{\mathrm{event}}(t)\right]x_j(t), \quad \text{(Supplementary Eq. 15)}$$

where A_{burst} is the learning rate for the plasticity rule and $x_j(t)$ is the trace of the presynaptic spike trains as defined by Eq. 25 from the main text with characteristic time τ_{pre}. The variable $S_{\mathrm{burst}}(t)$ is defined as

$$S_{\mathrm{burst}}(t) = \sum_k b_{k,\mathrm{post}}^*\delta\left(t - t_{k,\mathrm{post}}^*\right), \quad \text{(Supplementary Eq. 16)}$$

where $t_{k,\mathrm{post}}^*$ is the timing of the k*th* postsynaptic spike and $b_{k,\mathrm{post}}^*$ is 1 if $t_{k,\mathrm{post}}^*$ is the second spike in a burst and zero otherwise,

$$b_{k,\mathrm{post}}^* = \begin{cases}1, & \text{if } t_{k,\mathrm{post}} - t_{k-1,\mathrm{post}} \leqslant b_{\mathrm{th}}\\ 0, & \text{otherwise,}\end{cases} \quad \text{(Supplementary Eq. 17)}$$

where b_{th} is a threshold that defines a burst. The variable $y_{\mathrm{burst}}(t)$ is a trace of the postsynaptic bursts, described by following dynamics,

$$\frac{\mathrm{d}y_{\mathrm{burst}}(t)}{\mathrm{d}t} = -\frac{y_{\mathrm{burst}}(t)}{\tau_{\mathrm{avg}}} + S_{\mathrm{burst}}(t), \quad \text{(Supplementary Eq. 18)}$$

with characteristic time τ_{avg}. The variable $S_{\mathrm{event}}(t)$ is the timing of an event, defined as the time of a single spike or the time of the first spike of a burst,

$$S_{\mathrm{event}}(t) = \sum_k e_{k,\mathrm{post}}^*\delta\left(t - t_{k,\mathrm{post}}^*\right), \quad \text{(Supplementary Eq. 19)}$$

where $e_{k,\mathrm{post}}^*$ is 1 if $t_{k,\mathrm{post}}^*$ is a single spike or the first spike in a burst and zero otherwise,

$$e_{k,\mathrm{post}}^* = \begin{cases}1, & \text{if } t_{k,\mathrm{post}} - t_{k-1,\mathrm{post}} > b_{\mathrm{th}}\\ 0, & \text{otherwise.}\end{cases} \quad \text{(Supplementary Eq. 20)}$$

The variable $y_{\mathrm{event}}(t)$ is a trace of the postsynaptic events, described by following dynamics,

$$\frac{\mathrm{d}y_{\mathrm{event}}(t)}{\mathrm{d}t} = -\frac{y_{\mathrm{event}}(t)}{\tau_{\mathrm{avg}}} + S_{\mathrm{event}}(t). \quad \text{(Supplementary Eq. 21)}$$

Voltage-based excitatory plasticity rule. The excitatory weight of the j*th* connection changed according to

$$\frac{\mathrm{d}w_j(t)}{\mathrm{d}t} = A_{\mathrm{LTP}}x_j^+(t)\left[u(t) - \theta_+\right]_+\left[u^+(t) - \theta_-\right]_+ - A_{\mathrm{LTD}}\frac{\overline{u_2^-}(t)}{u_{\mathrm{ref}}^2}\left[u^-(t) - \theta_-\right]_+ S_j(t), \quad \text{(Supplementary Eq. 22)}$$

where A_{LTP} and A_{LTD} are the learning rates for LTP and LTD, respectively, $x_j^+(t)$ is the trace of the presynaptic spike train (dynamics according to Eq. 25 from the main text) with characteristic time τ_+, θ_+ is the voltage threshold for LTP, θ_- is the voltage threshold (for the voltage low pass-filter) for LTP and LTD, $u(t)$ is the membrane potential of the postsynaptic neuron, and $S_j(t)$ is the presynaptic spike train according to Eq. 8 from the main text. The voltage-based learning rule depends on the low-pass filter of the postsynaptic neuron's membrane potential, which creates a dependency on the recent history of membrane potential. There are three low-pass filters in the voltage-based model: one for LTP, $u^+(t)$, and two for LTD, $u^-(t)$ and $\overline{u^-}(t)$, with dynamics given by

$$\tau_+\frac{\mathrm{d}u^+(t)}{\mathrm{d}t} = -u^+(t) + u(t), \quad \text{(Supplementary Eq. 23)}$$

$$\tau_- \frac{\mathrm{d}u^-(t)}{\mathrm{d}t} = -u^-(t) + u(t), \qquad \text{(Supplementary Eq. 24)}$$

and

$$\tau_\mathrm{u} \frac{\mathrm{d}\overline{u^-}(t)}{\mathrm{d}t} = -\overline{u^-}(t) + [u(t) - u_\mathrm{rest}]^2\,, \qquad \text{(Supplementary Eq. 25)}$$

where τ_+ and τ_- are the time constants for the voltage low-pass filters for LTP and LTD, respectively, and τ_u is the time constant for the low-pass filter for the membrane potential deviations from the resting membrane potential.

To account for the plasticity inducing effects of the action potential, we modified the LIF neuron model so that it had a short depolarised period corresponding to the action potential based on previous description from ModelDB[6]. Following the implementation from the subsection *Point neuron - Fixed spiking threshold* (from the Methods of the main text), when the membrane potential crossed the threshold, u_th, from below, the membrane potential was instantaneously set to $u_{AP} = 30$ mV, and it was then clamped at this voltage for the duration of the action potential $\tau_\mathrm{AP} = 2$ ms. After the duration of the action potential, the membrane potential was instantaneously reset to $u^\mathrm{volt}_\mathrm{reset} = -52$ mV. In Extended Data Fig. 2c,g,k, we did not implement the low-pass filter $\overline{u_2^-}(t)$, i.e., $\left(\overline{u_2^-}(t)/u^2_\mathrm{ref}\right) = 1, \forall t$, according to refs. 6 and 7.

Spike-based inhibitory plasticity rule. We implemented the inhibitory plasticity model from Vogels *et al.*[3], which follows a Hebbian-like shape that imposes a postsynaptic firing-rate set-point. The strength of the j*th* connection to the postsynaptic neuron evolved as

$$\frac{\mathrm{d}w_j(t)}{\mathrm{d}t} = A_\mathrm{ISP}\left\{x_j(t)S_\mathrm{post}(t) + \left[y_\mathrm{post}(t) - \alpha\right]S_j(t)\right\}, \qquad \text{(Supplementary Eq. 26)}$$

where A_ISP is the learning rate, $x_j(t)$ and $y_\mathrm{post}(t)$ are traces of pre- and postsynaptic spike trains, respectively, following the same dynamics described in Eqs. 29 and 30 from the main text, with the same time constant τ_iSTDP for both traces. The spike trains of post- and presynaptic neurons, $S_\mathrm{post}(t)$ and $S_j(t)$, are described by Eqs. 6 and 8, respectively. This learning rule imposes in a stable firing-rate set-point for postsynaptic neurons given by[3]

$$\nu_0^\mathrm{I} = \frac{\alpha}{2\tau_\mathrm{iSTDP}}. \qquad \text{(Supplementary Eq. 27)}$$

Backpropagating action potential and voltage-dependent STDP. To test the influence of a backpropagating action potential (bAP) in the voltage-dependent STDP protocol simulation (Fig. 2b), we modified the postsynaptic neuron model (Eq. 3 from the main text), according to

$$\begin{aligned}\tau_\mathrm{m}\frac{\mathrm{d}u(t)}{\mathrm{d}t} =& -[u(t) - u_\mathrm{rest}] - g_\mathrm{AMPA}(t)\,[u(t) - E_\mathrm{AMPA}] \\ &- g_\mathrm{NMDA}(t)H_\mathrm{NMDA}(u(t))\,[u(t) - E_\mathrm{NMDA}] - g_\mathrm{bAP}(t)\,[u(t) - E_\mathrm{bAP}]\,,\end{aligned} \qquad \text{(Supplementary Eq. 28)}$$

where all variables and parameters except $g_\mathrm{bAP}(t)$ and E_bAP are explained in the Methods from the main text. The variable $g_\mathrm{bAP}(t)$ represents the conductance of the channels that open due to a backpropagating action potential, with dynamics

$$\frac{\mathrm{d}g_\mathrm{bAP}(t)}{\mathrm{d}t} = -\frac{g_\mathrm{bAP}(t)}{\tau_\mathrm{bAP}} + A_\mathrm{bAP}S_\mathrm{post}(t), \qquad \text{(Supplementary Eq. 29)}$$

where τ_bAP is the characteristic time of the channels, $S_\mathrm{post}(t)$ is the postsynaptic spike train (defined in Eq. 6 from the main text), and A_bAP is the amplitude of the increase in the channels' conductance due to a postsynaptic spike. Supplementary Fig. 1 shows four examples of the dynamics of the postsynaptic neuron's membrane potential for different amplitudes of the bAP, with and without excitatory input spike 10 ms before the postsynaptic spike. We performed a parameter sweep in the parameters $w_\mathrm{E}(t = 0) = [2 \times 10^{-2}, 20]$ nS, $I_\mathrm{ext} = [0, 0.1]$ nA, and $A_\mathrm{bAP} = [0, 30]$ nS, and calculated the average depolarisation during the 200 ms interval starting at the first presynaptic spike in each burst (Supplementary Fig. 1).

Average voltage in the frequency-dependent STDP protocol. To highlight the explicit dependency of our model on the activation of neighbouring excitatory and inhibitory synapses, we simulated the frequency-dependent STDP protocol from Sjöström *et al.*[8] with the addition of Poisson activity of one excitatory and one inhibitory synapse nearby the one being stimulated (Fig. 2e,f and Extended Data Fig. 2d-k). We chose the firing-rate and weight of the neighbouring synapses such that the postsynaptic neuron's membrane potential would remain, on average, at rest. Supplementary Fig. 2a shows the membrane potential for three examples of such simulations with different presynaptic firing-rates and the evolution of the low-pass filters of the membrane potential used in the voltage-based

excitatory learning rule (Supplementary Eq. 23 and Supplementary Eq. 24). When receiving external inputs, the postsynaptic neuron's membrane potential fluctuates around the same values of the simulation without external inputs. The average of the membrane potential over trials remained mostly unchanged (Supplementary Fig. 2b).

Importantly, an increase in the firing-rate of presynaptic neuron j, ν_j, results in an increase in the co-dependent excitatory plasticity model's variable $E_j(t)$, even for the same (constant) postsynaptic membrane potential. To show this, we calculated the average NMDA excitatory current of the jth synapse based on Eq. 17 from the main text, which can be written as (considering that the postsynaptic neuron's membrane potential is at rest, u_{rest})

$$\begin{aligned}\left\langle \widetilde{E}_j(t) \right\rangle_t &= \langle g_{\text{NMDA}}(t) \rangle_t H_{\text{NMDA}}(u_{\text{rest}})(u_{\text{rest}} - E_{\text{NMDA}}) \\ &= \nu_j \tau_{\text{NMDA}} w_j H_{\text{NMDA}}(u_{\text{rest}})(u_{\text{rest}} - E_{\text{NMDA}}),\end{aligned} \qquad \text{(Supplementary Eq. 30)}$$

where w_j is the jth synapse's efficacy, and all other parameters are described in the Methods section of the main text. Thus, higher presynaptic firing-rate (through variable ν_j) increases NMDA currents, which results in an increase in the variable $E_j(t)$, potentially leading to stronger LTP.

Receptive field plasticity model comparison. Here we describe the simulations shown in Supplementary Fig. 3 in which we use different models for the paradigm of receptive field plasticity.

Weight-dependent spike-timing-dependent plasticity model. In Supplementary Fig. 3a, we implemented a weight-dependent spike-based learning rule for excitatory connections (Supplementary Eq. 11 with $\gamma_{\text{E}} = 1$), and the spike-based inhibitory plasticity rule for inhibitory connections (Supplementary Eq. 26). The weight-dependent spike-based learning rule (Supplementary Eq. 11 with $\gamma_{\text{E}} = 1$) has a fixed-point for the excitatory weights given by,

$$\langle w \rangle = \frac{A_{\text{LTP}} \tau_+}{A_{\text{LTD}} \tau_-}. \qquad \text{(Supplementary Eq. 31)}$$

We set the parameters such that $\langle w \rangle \approx 1.2$ nS. After excitatory and inhibitory weights stabilised, we hand-tuned the excitatory weights in the shape of a receptive field (★1 in Supplementary Fig. 3a). The modified excitatory weights returned to the fixed-point without retaining the receptive field's shape. After 26 minutes of simulation time, we implemented a similar input pattern from Extended Data Fig. 4a, but only with a single input pathway activated for 10 seconds, which failed to create a receptive field profile (★3 in Supplementary Fig. 3a).

Weight-independent spike-timing-dependent plasticity model. In Supplementary Fig. 3b, we implemented a classic weight-independent spike-based learning rule for excitatory connections (Supplementary Eq. 11 with $\gamma_{\text{E}} = 0$), and the spike-based inhibitory plasticity rule for inhibitory connections (Supplementary Eq. 26). After 10 minutes of simulated time, we hand-tuned the excitatory weights in the shape of a receptive field (★1 in Supplementary Fig. 3b), which resulted in the connections from the preferred pathway reaching the maximum allowed values while all other connections vanished, creating a winner-take all receptive field profiled which was stable for hours. Reshaping of the receptive field was not possible when we increased the input onto a single different pathway while decreasing inhibitory inputs for 5 seconds (★3 in Supplementary Fig. 3b).

Orchestrated spike-timing-dependent plasticity model. In Supplementary Fig. 3c, we implemented the orchestrated spike-based plasticity model[5] (Supplementary Eq. 12), and the spike-based inhibitory plasticity rule for inhibitory connections (Supplementary Eq. 26). After 10 minutes of simulated time, we implemented a similar input pattern from Extended Data Fig. 4a, but with only a single input pathway activated for 6 seconds, which resulted in the connections from the preferred pathway becoming stronger than all other connections, creating a receptive field profile which was stable for hours. Reshaping of the receptive field was not possible when we increased the input onto a single different pathway while decreasing inhibitory inputs for 6 seconds (★3 in Supplementary Fig. 3c).

Voltage-dependent plasticity model. In Supplementary Fig. 3d, we implemented a voltage-dependent plasticity model[7] (Supplementary Eq. 26), and the spike-based inhibitory plasticity rule for inhibitory connections (Supplementary Eq. 26). After 10 minutes of simulated time, we implemented a similar input pattern from Extended Data Fig. 4a, but with only a single input pathway activated for 0.2 seconds, which resulted in the connections from the preferred pathway becoming stronger than all other connections, creating a receptive field profile that was stable for hours. After 11 hours, we increased the input onto a different pathway while decreasing inhibitory inputs (★3 in Supplementary Fig. 3d), which strengthened the connections belonging to the most recently activated pathway. Two pathways remained with weights stronger than the other connections for hours, eventually weakening but retaining a receptive field profile with two preferred pathways.

Burst-dependent spike-based plasticity model. In Supplementary Fig. 3e, we implemented the burst-dependent spike-based plasticity model[9] (Supplementary Eq. 15), and the spike-based inhibitory plasticity rule for inhibitory connections (Supplementary Eq. 26). After 10 minutes of simulated time, we implemented a specific input pattern to induce short postsynaptic bursts during the activation of the preferred pathway. During the learning period (2 seconds), the active pathway had short intervals of 10-ms with high firing-rates, interleaved by a 60-ms intervals with background firing-rate, creating a receptive field profile which was stable for hours. After 11 hours of simulated time, we increased

the input onto a different pathway for 5 seconds, following the same 10-ms high firing-rate with a 60-ms interval ($\star$3 in Supplementary Fig. 3d), which did not elicit strong changes in excitatory weights. In the 10-ms intervals of high firing-rate during the learning period, presynaptic neurons had their probability of spiking set to $p_j = 0.02$.

SUPPLEMENTARY REFERENCES

[1] M. C. W. Van Rossum, G. Q. Bi, and G. G. Turrigiano, Stable hebbian learning from spike timing-dependent plasticity, Journal of Neuroscience **20**, 8812 (2000).

[2] J.-P. Pfister and W. Gerstner, Triplets of spikes in a model of spike timing-dependent plasticity, Journal of Neuroscience **26**, 9673 (2006).

[3] T. P. Vogels, H. Sprekeler, F. Zenke, C. Clopath, and W. Gerstner, Inhibitory plasticity balances excitation and inhibition in sensory pathways and memory networks, Science **334**, 1569 (2011).

[4] S. Song, K. D. Miller, and L. F. Abbott, Competitive hebbian learning through spike-timing-dependent synaptic plasticity, Nature Neuroscience **3**, 919 (2000).

[5] F. Zenke, E. J. Agnes, and W. Gerstner, Diverse synaptic plasticity mechanisms orchestrated to form and retrieve memories in spiking neural networks, Nature Communications **6**, 6922 (2015).

[6] Modeldb accession number 144566, `http://modeldb.yale.edu/144566`.

[7] C. Clopath, L. Büsing, E. Vasilaki, and W. Gerstner, Connectivity reflects coding: a model of voltage-based stdp with homeostasis, Nature Neuroscience **13**, 344 (2010).

[8] P. J. Sjöström, G. G. Turrigiano, and S. B. Nelson, Rate, timing, and cooperativity jointly determine cortical synaptic plasticity, Neuron **32**, 1149 (2001).

[9] A. Payeur, J. Guerguiev, F. Zenke, B. A. Richards, and R. Naud, Burst-dependent synaptic plasticity can coordinate learning in hierarchical circuits, Nature Neuroscience **24**, 1010 (2021).
