# Supplementary material for: Co-dependent excitatory and inhibitory plasticity accounts for quick, stable and long-lasting memories in biological networks
Source: Nat Neurosci. 2024 Mar 20;27(5):964–74. doi: 10.1038/s41593-024-01597-4 (PMC11089004; doi:10.1038/s41593-024-01597-4)
Supplement: Supplementary file 2 — Reporting Summary [file 41593_2024_1597_MOESM2_ESM.pdf]

nature portfolio

Corresponding author(s): Everton J. Agnes

Last updated by author(s): Feb 28, 2024

Reporting Summary

Nature Portfolio wishes to improve the reproducibility of the work that we publish. This form provides structure for consistency and transparency in reporting. For further information on Nature Portfolio policies, see our Editorial Policies and the Editorial Policy Checklist.

Statistics

For all statistical analyses, confirm that the following items are present in the figure legend, table legend, main text, or Methods section.

n/a	Confirmed	
☒	☐	The exact sample size (*n*) for each experimental group/condition, given as a discrete number and unit of measurement
☒	☐	A statement on whether measurements were taken from distinct samples or whether the same sample was measured repeatedly
☒	☐	The statistical test(s) used AND whether they are one- or two-sided<br>*Only common tests should be described solely by name; describe more complex techniques in the Methods section.*
☒	☐	A description of all covariates tested
☒	☐	A description of any assumptions or corrections, such as tests of normality and adjustment for multiple comparisons
☐	☒	A full description of the statistical parameters including central tendency (e.g. means) or other basic estimates (e.g. regression coefficient) AND variation (e.g. standard deviation) or associated estimates of uncertainty (e.g. confidence intervals)
☒	☐	For null hypothesis testing, the test statistic (e.g. *F*, *t*, *r*) with confidence intervals, effect sizes, degrees of freedom and *P* value noted<br>*Give P values as exact values whenever suitable.*
☒	☐	For Bayesian analysis, information on the choice of priors and Markov chain Monte Carlo settings
☒	☐	For hierarchical and complex designs, identification of the appropriate level for tests and full reporting of outcomes
☒	☐	Estimates of effect sizes (e.g. Cohen's *d*, Pearson's *r*), indicating how they were calculated

Our web collection on statistics for biologists contains articles on many of the points above.

Software and code

Policy information about availability of computer code

Data collection	All simulations were performed using Fortran, compiled with Intel Fortran 19.0.1.144. Relevant code is available at https://github.com/ejagnes/codependent_plasticity/. Spike-timing-dependent plasticity data (refs. 15 and 16) is publicly available from http://plasticity.muhc.mcgill.ca/page8.html. Receptive-field plasticity data (ref. 21) was obtained from the authors. Spike-timing-dependent plasticity data of neighbouring excitatory synapses (ref. 24) and coupling strength vs electrotonic distance data (ref. 45) were extracted using open source software (https://automeris.io/WebPlotDigitizer/).
Data analysis	Principal Component Analysis of the recurrent network activity was done with Matlab 2020b.

For manuscripts utilizing custom algorithms or software that are central to the research but not yet described in published literature, software must be made available to editors and reviewers. We strongly encourage code deposition in a community repository (e.g. GitHub). See the Nature Portfolio guidelines for submitting code & software for further information.

Data

Policy information about availability of data

All manuscripts must include a data availability statement. This statement should provide the following information, where applicable:

- Accession codes, unique identifiers, or web links for publicly available datasets
- A description of any restrictions on data availability
- For clinical datasets or third party data, please ensure that the statement adheres to our policy

Spike-timing-dependent plasticity data (refs. 15 and 16) is publicly available from http://plasticity.muhc.mcgill.ca/page8.html.

Research involving human participants, their data, or biological material

Policy information about studies with human participants or human data. See also policy information about sex, gender (identity/presentation), and sexual orientation and race, ethnicity and racism.

Reporting on sex and gender	Our study did not include human research participants.
Reporting on race, ethnicity, or other socially relevant groupings	Our study did not include human research participants.
Population characteristics	Our study did not include human research participants.
Recruitment	Our study did not include human research participants.
Ethics oversight	Our study did not include human research participants.

Note that full information on the approval of the study protocol must also be provided in the manuscript.

Field-specific reporting

Please select the one below that is the best fit for your research. If you are not sure, read the appropriate sections before making your selection.

☒ Life sciences ☐ Behavioural & social sciences ☐ Ecological, evolutionary & environmental sciences

For a reference copy of the document with all sections, see nature.com/documents/nr-reporting-summary-flat.pdf

Life sciences study design

All studies must disclose on these points even when the disclosure is negative.

Sample size	No statistical test was used in our work and no sample size calculation was performed. We ran 10 simulations of a single postsynaptic neuron receiving homogeneous Poisson spike trains for a given combination of model parameters, each simulation with a different sequence of spike trains (Fig. 3e-g). We simulated four combinations of two parameters of the excitatory plasticity model to confirm the independence of the steady-state solution (total NMDA current) from these two parameters. We ran 1000 simulations of the recurrent network during recall (Fig. 8 and Extended Data Fig. 10) to compensate for the the trial-to-trial variability of individual spike trains. This number was chosen to be sufficiently large to accurately describe the mean instantaneous activity of each neuron per stimulus pattern.
Data exclusions	No data were excluded.
Replication	Though technically this is not applicable to mathematical models, it's worth noting that we checked robustness of the model by running multiple simulations with different random seeds. The relevant code used for the simulations reported in our study is available for the purpose of replication.
Randomization	Randomization was not relevant to our study because we only ran simulations (no experimental data was collected), whose results are unaffected by execution order.
Blinding	Blinding was not relevant to our study because it did not involve group allocation.

Reporting for specific materials, systems and methods

We require information from authors about some types of materials, experimental systems and methods used in many studies. Here, indicate whether each material, system or method listed is relevant to your study. If you are not sure if a list item applies to your research, read the appropriate section before selecting a response.

Materials & experimental systems

n/a	Involved in the study
☒	☐ Antibodies
☒	☐ Eukaryotic cell lines
☒	☐ Palaeontology and archaeology
☒	☐ Animals and other organisms
☒	☐ Clinical data
☒	☐ Dual use research of concern
☒	☐ Plants

Methods

n/a	Involved in the study
☒	☐ ChIP-seq
☒	☐ Flow cytometry
☒	☐ MRI-based neuroimaging
